# Nexus between genome-wide copy number variations and autism spectrum disorder in Northeast Han Chinese population

**DOI:** 10.1186/s12888-023-04565-7

**Published:** 2023-02-07

**Authors:** Shuang Qiu, Yingjia Qiu, Yong Li, Xiaojuan Zhu, Yunkai Liu, Yichun Qiao, Yi Cheng, Yawen Liu

**Affiliations:** 1 Department of Epidemiology and Biostatistics, School of Public Health, Jilin University, Changchun, 130021 Jilin China; 2 Department of Laboratory Medicine, Jilin University Hospital, Changchun, 130000 Jilin China; 3 China-Japan Union Hospital, Jilin University, Changchun, 130033 Jilin China; 4 The Key Laboratory of Molecular Epigenetics of Ministry of Education, Institute of Cytology and Genetics, Northeast Normal University, Changchun, 130021 Jilin China; 5 Department of Cardiovascular Diseases, the First Hospital of Jilin University, Changchun, 130021 Jilin China; 6 Key Laboratory for Cardiovascular Mechanism of Traditional Chinese Medicine, Changchun, 130021 Jilin China; 7 Institute of Translational Medicine, the First Hospital of Jilin University, Changchun, 130021 Jilin China

**Keywords:** Autism spectrum disorder, Copy number variations, MicroRNAs, Array-based comparative genomic hybridization

## Abstract

**Background:**

Autism spectrum disorder (ASD) is a common neurodevelopmental disorder, with an increasing prevalence worldwide. Copy number variation (CNV), as one of genetic factors, is involved in ASD etiology. However, there exist substantial differences in terms of location and frequency of some CNVs in the general Asian population. Whole-genome studies of CNVs in Northeast Han Chinese samples are still lacking, necessitating our ongoing work to investigate the characteristics of CNVs in a Northeast Han Chinese population with clinically diagnosed ASD.

**Methods:**

We performed a genome-wide CNVs screening in Northeast Han Chinese individuals with ASD using array-based comparative genomic hybridization.

**Results:**

We found that 22 kinds of CNVs (6 deletions and 16 duplications) were potentially pathogenic. These CNVs were distributed in chromosome 1p36.33, 1p36.31, 1q42.13, 2p23.1-p22.3, 5p15.33, 5p15.33-p15.2, 7p22.3, 7p22.3-p22.2, 7q22.1-q22.2, 10q23.2-q23.31, 10q26.2-q26.3, 11p15.5, 11q25, 12p12.1-p11.23, 14q11.2, 15q13.3, 16p13.3, 16q21, 22q13.31-q13.33, and Xq12-q13.1. Additionally, we found 20 potential pathogenic genes of ASD in our population, including eight protein coding genes (six duplications [*DRD4*, *HRAS*, *OPHN1*, *SHANK3*, *SLC6A3*, and *TSC2*] and two deletions [*CHRNA7* and *PTEN*]) and 12 microRNAs-coding genes (ten duplications [*MIR202*, *MIR210*, *MIR3178*, *MIR339*, *MIR4516*, *MIR4717*, *MIR483*, *MIR675*, *MIR6821*, and *MIR940*] and two deletions [*MIR107* and *MIR558*]).

**Conclusion:**

We identified CNVs and genes implicated in ASD risks, conferring perception to further reveal ASD etiology.

**Supplementary Information:**

The online version contains supplementary material available at 10.1186/s12888-023-04565-7.

## Background

Autism spectrum disorder (ASD) is a common neurodevelopmental disorder with an increasing prevalence worldwide [1, 2]. ASD manifests the wide range of symptoms and severity in perceivability and socialization with others, such as limited and repetitive patterns of behavior. Both genetic and environmental factors are involved in ASD pathogenesis. Environmental factors, including viral infections, medications during pregnancy, and air pollutants, may contribute to ASD risks [3]. Compared with environmental factors, genetic factors appear to be a prerequisite for ASD development: genetic changes (mutations) may increase ASD risks; additionally, genes, such as *CHD8* [4], *CNTNAP2* [5], *DCC* [6], neurexin genes [7], *SHANK1* [8], *SHANK2* [9], *SHANK3* [10], and *WNT2* [11] may affect brain development or brain-cells communication. ASD heritability has been estimated to be 50%, reflecting that genetic factors afford main components in ASD etiology [12].

ASD begins in early childhood. Children with ASD usually show symptoms of autism within the first year, and regress during a period between one and two years of age. Although there is no specific medication for ASD patients [13], early treatment can confer the lives of children with ASD beneficially. Gene-based test provides an impressive opportunity to identify potential infants with ASD [8].

Accumulating whole-genome, association, and linkage studies have strongly documented the roles of genes in ASD [14–16]. Copy-number variants (CNVs) are defined as deletions and duplications of DNA segments in the genome greater than one kilobase (Kb) [17, 18]. De novo CNV events have been found to be implicated in the etiology of depression, schizophrenia, bipolar disorder, attention deficit hyperactivity disorder, and ASD [19–22]. Array-based comparative genomic hybridization (aCGH) technology has proven to be a rapid method to detect the association between CNVs and ASD risks [23, 24]. Large simplex ASD cohort studies show that the rate of rare de novo CNVs is significantly higher in affected siblings (5.8–7.9%) than that in unaffected siblings (1.7–1.9%) [25, 26]. CNVs at 1q21.1, 2p16.3, 3q29, 7q11.23, 15q11.2–13.1, 16p11.2, 17p11.2, 17q12, and 22q11.2 are associated with ASD risks [24, 27]. Moreover, CNVs in *NRXN1*, *SETD5*, *HDAC9*, and *PARK2* are found to be associated with ASD risks [28–30]. However, there exist substantial differences in terms of location and frequency of some CNVs in the general Asian population [31]. In this paper, we investigated in CNVs in Northeast Han Chinese individuals with ASD.

## Methods

### Study subjects

We enrolled 16 individuals with ASD aged 2 to 7 years from the Chunguang Rehabilitation hospital in Jilin Province, after cases with fragile X syndrome, Rett syndrome, chromosomal abnormalities, or any neurological or psychiatric disorders were excluded. The individuals with ASD were diagnosed by Pediatric Neurology and Neurorehabilitation doctors using the Diagnostic and Statistical Manual of Mental Disorders (5^th^ edition) [32]. All the individuals with ASD were northeast Han Chinese.

### DNA extraction and Detection of CNVs

Genomic DNA was extracted from peripheral blood samples using DNA extraction kits, according to the manufacturer’s instructions (DP319 TIANamp Blood DNA Kit, TIANGEN BiotechCo. Ltd, Beijing, China) [33]. We used Nano Drop (Cat#ND-1000, ThermoFisher, Waltham, MA, US) and 1% agarose gel electrophoresis to check the quantity and quality of the isolated DNA. We used aCGH for genome-wide CNVs screening (Agilent SurePrint G3 Human CGH 60 K). Male and female DNA samples were hybridized with male and female reference DNA samples (G1471, G1521, Promega), respectively.

### aCGH data analysis

We converted the raw data using FEATURE EXTRACTION software 10.7 and analyzed CNVs using Agilent CytoGenomics software 4.0.3.12 (Agilent technologies, Santa Clara, CA, US). The human genome assembly NCBI36/hg18 was used as a reference. The analysis settings for CNVs calling were Aberration Detection Method 2 algorithm, centralization threshold 6, bin size 10, and minimum number of adjacent probes 3. Thresholds were set via log2-ratio (log_2_^R^) (for detecting duplications, log_2_^R^ ≥ 0.25; for detecting deletions, log_2_^R^ ≤ -0.25).

### Identification of potential pathogenic CNVs of ASD

We calculated the frequency of each overlapping or non-overlapping CNV in DNA samples from our subjects. CNVs with same overlapping sequence were defined as one kind of CNV, and a non-overlapping CNV was also sorted as one kind of CNV. The circular plot of CNVs distribution in chromosome was visualized using circlize package in R3.6.2 software [34]. We converted our bed file from exon coordinates for human build NCBI36 (hg18) into GRCh37 (hg19) using UCSC LiftOver tool (http://genome.ucsc.edu/cgi-bin/hgLiftOver). The classification of CNVs was based on Database of Genomic Variants (DGV, http://dgv.tcag.ca/dgv/app/home), Database of Genomic Structural Variation (dbVar, https://www.ncbi.nlm.nih.gov/dbvar), Clinical Genome Resources (ClinGen, https://clinicalgenome.org/), and Online Mendelian Inheritance in Man (OMIM, https://www.ncbi.nlm.nih.gov/omim). CNVs were classified as benign, likely benign, a variant of unknown significance (VOUS), likely pathogenic, and pathogenic using AnnotSV program (https://lbgi.fr/AnnotSV/) according to American College of Medical Genetics guideline [35].

CNVs were considered of strong putative interest when they reached the following criteria: (1) they were classified as likely pathogenic or pathogenic; (2) they were of large size (> 100 kb); (3) they had been found in the knowledgebases for the genetic evidence of ASD (Simons Foundation Autism Research Initiative [SFARI, https://www.sfari.org/resource/sfari-gene/], or AutismKB [http://www.autismkb.com]); (4) they had been found in the Database of genomic variation and phenotype in Humans using Ensembl Resources (DECIPHER, https://decipher.sanger.ac.uk/about#overview); and (5) they contained previously reported ASD-relative genes.

### Identification of potential pathogenic genes of ASD

We selected potential pathogenic genes within potential pathogenic CNVs on the basis of the following criteria: (1) genes enriched in ASD-related pathways; and (2) same genes shared with 363 genes in SFARI classified as high-confidence or strong-candidate, or with 228 genes in AutismKB classified as high-confidence.

### Identification of potential pathogenic microRNAs of ASD

MicroRNAs (miRNAs) are involved in the pathogenesis of ASD [30, 36]. Because genes implicated in CNVs that we found encode miRNAs, we further selected potential-pathogenic-CNVs-encoded miRNAs by retrieving PubMed according to experimental evidence documenting nervous system dysfunction.

### Bioinformatic analysis

The Gene Ontology (GO) and KEGG pathway analyses of the genes from potential pathogenic CNVs were performed using clusterProfiler package in R3.6.2 software [37, 38]. *P*-value < 0.05 was considered statistically significant. miRWalk 2.0 database, which contained 12 miRNA-target-prediction database, was used to predict target genes of CNVs-encoded miRNAs [39]. We selected the target genes according to the criteria—target genes existed in at least seven of the 12 databases. Moreover, interactive relationship between CNVs-encoded miRNAs and target genes was presented using Cytoscape 3.8.0 (http://www.cytoscape.org/).

## Results

### Identification of CNVs

To detect CNVs, aCGH was performed in all DNA samples from the 16 subjects with ASD (13 males and 3 females). We identified 364 CNVs (153 deletions and 211 duplications) with an average genomic size of 211.982 kb (114.091 kb for deletions and 258.705 kb for duplications). The mean number of CNVs per subject was 22.750 (9.563 for deletions and 13.188 for duplications). The mean number of deletions in male (10.462) was greater than that in females (5.667) (Table 1).

**Table 1:** The Characteristics of genome-wide CNVs among our subjects

Characters	Number (proportion %) of CNVs	Median CNV Size (kb)	Mean Number of CNVs per Subject
Total	364 (100.0)	211.982 (78.813, 705.031)	22.750
Male	299 (82.1)	213.241 (81.797, 864.947)	23.000
Female	65 (17.9)	132.360 (73.256, 305.926)	21.667
Duplication	211 (58.0)	258.705 (86.903, 708.853)	13.188
Male	163 (77.3)	305.926 (86.903, 1092.277)	12.538
Female	48 (22.7)	133.712 (87.668, 377.979)	16.000
Deletion	153 (42.0)	114.091 (73.256, 656.149)	9.563
Male	136 (88.9)	114.518 (73.256, 693.563)	10.462
Female	17 (11.1)	73.256 (60.021, 135.433)	5.667

A total of 13 males and 3 females

### Identification potential pathogenic CNVs of ASD

A total of 20 CNVs from 364 CNVs failed to be converted to GRCh37 (hg19); thus, we obtained 72 benign, 65 likely benign, 9 VOUS, 167 likely pathogenic, and 31 pathogenic CNVs (Table 2). We found that more than half CNVs were likely pathogenic or pathogenic.

**Table 2:** The Classification of CNVs based on ACMG

Classification	Total (%)	Duplication (%)	Deletion (%)
Benign	72 (19.8)	35 (16.6)	37 (24.2)
Likely Benign	65 (17.9)	59 (28.0)	6 (3.9)
VOUS	9 (2.5)	9 (4.3)	0 (0.0)
Likely Pathogenic	167 (45.9)	68 (32.2)	99 (64.7)
Pathogenic	31 (8.5)	28 (13.3)	3 (2.0)

*VOUS* variant of unknown significance; A total of 20 CNVs failed to be converted to GRCh37 (hg19), thus, the total proportion was not equal to 100%. *ACMG* American College of Medical Genetics guideline

**Fig. 1:**
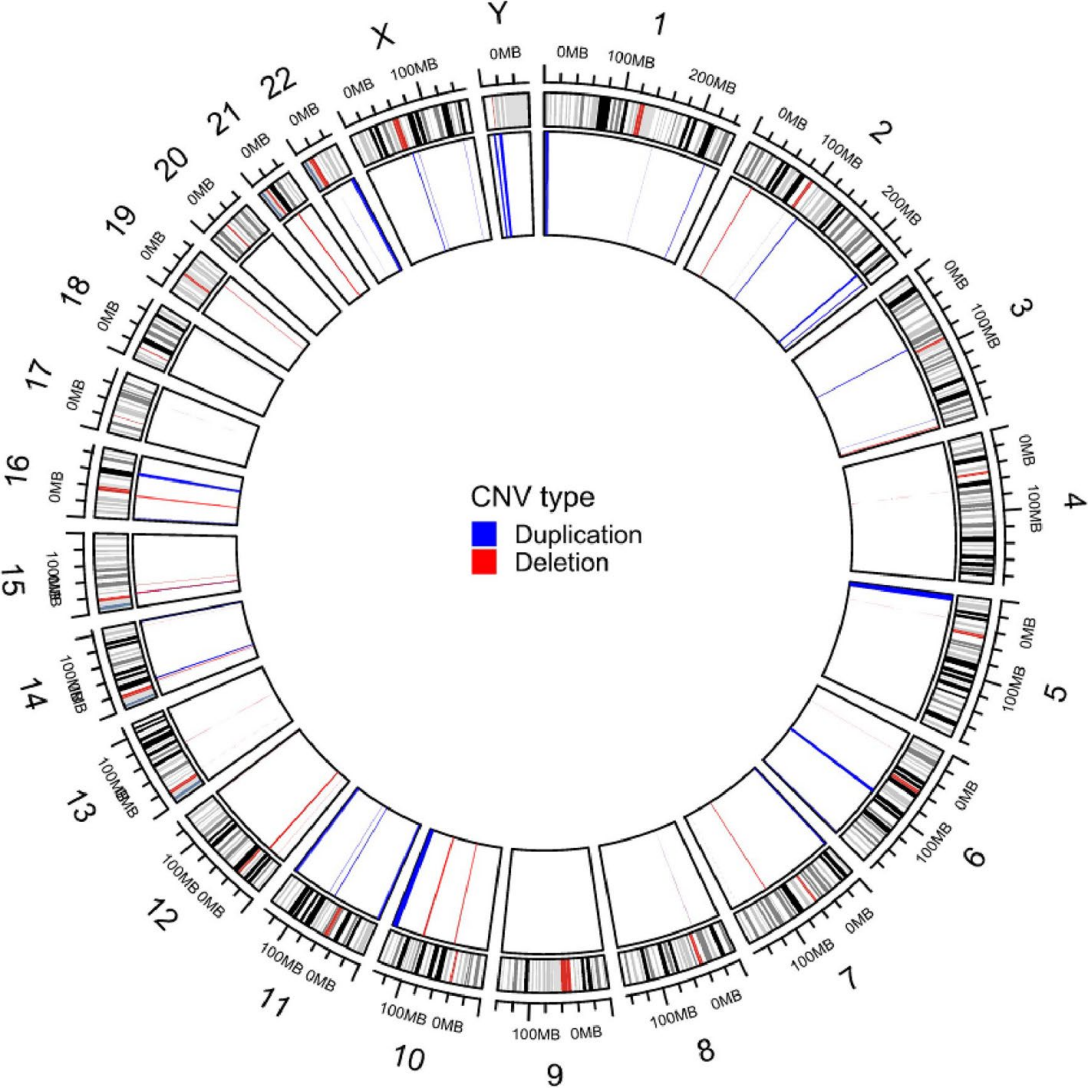
The distribution of CNVs on genome-wide chromosomes

After we calculated the frequency of each overlapping or non-overlapping CNV in DNA samples from our subjects, 344 CNVs were converted into 115 kinds of CNVs (45 deletions and 70 duplications). All the 115 kinds of CNVs were further classified (benign: 13 kinds; likely benign: 18 kinds; VOUS: two kinds; likely pathogenic: 60 kinds; and pathogenic: 13 kinds) (Supplementary Table 1). The distribution of the 115 kinds of CNVs in chromosome is visualized by circular plot (Fig. 1).

We investigated SFARI, AutismKB, and DECIPHER database to identify potential pathogenic CNVs from the 115 kinds of CNVs, revealing that 22 kinds of CNVs (6 deletions and 16 duplications) were potentially pathogenic. The 22 kinds of CNVs were distributed in chromosome 1, 2, 5, 7, 10, 11, 12, 14, 15, 16, 22, and X. Among them, 19 kinds of CNVs were rare (Table 3).

**Table 3:** Summary of candidate CNVs of ASD

M/F No	Coordinates, hg18	Cytoband	Size (Kb)	CNV Type	Classification	Number of genes	Gene Name
1/0	chr1:1,179,223–2,271,500	1p36.33	1092.277	Duplication	LP	42	DVL1, TMEM52
1/0	chr1:5,998,727–6,334,157	1p36.31	335.430	Duplication	LP	9	CHD5
4/0	chr1:225,876,894–226,738,916	1q42.13	862.022	Duplication	LP	26	PRSS38
1/0	chr2:31,412,158–32,712,484	2p23.1-p22.3	1300.327	Deletion	P	12	BIRC6, SPAST, SRD5A2
1/0	chr5:360,041–873,365	5p15.33	513.324	Duplication	LP	11	AHRR, EXOC3, PDCD6
1/0	chr5:1,115,468–8,452,427	5p15.33-p15.2	7336.959	Duplication	P	49	ADCY2, SLC6A3, TERT
2/0	chr7:524,935–1,037,461	7p22.3	512.526	Duplication	LP	13	ADAP1, PRKAR1B
1/0	chr7:1,037,461–2,536,804	7p22.3-p22.2	1499.343	Duplication	LP	26	INTS1
1/0	chr7:103,622,888–104,803,388	7q22.1-q22.2	1180.501	Deletion	LP	8	KMT2E, LHFPL3
13/0	chr10:89,540,133–91,524,263	10q23.2-q23.31	1984.131	Deletion	LP	31	PTEN
1/0	chr10:127,658,856–135,254,513	10q26.2-q26.3	7595.658	Duplication	P	58	DOCK1, EBF3, GLRX3
5/2	chr11:498,019–2,179,368	11p15.5	1681.349	Duplication	P	84	BRSK2, CD151, CTSD, DEAF1, DRD4, HRAS, IGF2, PHRF1, TALDO1
3/0	chr11:132,773,688–134,043,707	11q25	1270.019	Duplication	LP	16	IGSF9B
6/0	chr12:25,156,062–27,414,420	12p12.1-p11.23	2258.359	Deletion	LP	17	KRAS, MED21
1/0	chr14:22,086,438–22,354,007	14q11.2	267.569	Deletion	LP	4	SLC7A7
1/0	chr15:29,809,025–30,298,155	15q13.3	489.131	Deletion	P	1	CHRNA7
0/2	chr16:2,021,433–2,484,806	16p13.3	463.373	Duplication	LP	32	PGP, PKD1, RNPS1, SLC9A3R2, TRAF7, TSC2
0/3	chr16:2,484,806–2,747,528	16p13.3	262.722	Duplication	LP	13	SRRM2
2/0	chr16:61,464,644–64,965,235	16q21	3500.591	Duplication	LP	4	CDH11
4/0	chr22:46,395,224–49,412,774	22q13.31-q13.33	3017.550	Duplication	P	44	CHKB, MAPK12, MAPK8IP2, PANX2, PPP6R2, SBF1, TRABD
2/0	chr22:49,412,774–49,525,130	22q13.31-q13.33	112.356	Duplication	P	3	SHANK3
1/0	chrX:67,331,017–68,768,438	Xq12-q13.1	1437.422	Duplication	P	8	OPHN1

*M* Male, *F* Female. M/F No. means the number of CNV among male/female. *P* Pathogenic, *LP* Likely Pathogenic. The genes were reported to be related with ASD

### Identification of potential pathogenic genes with CNVs of ASD

**Fig. 2:**
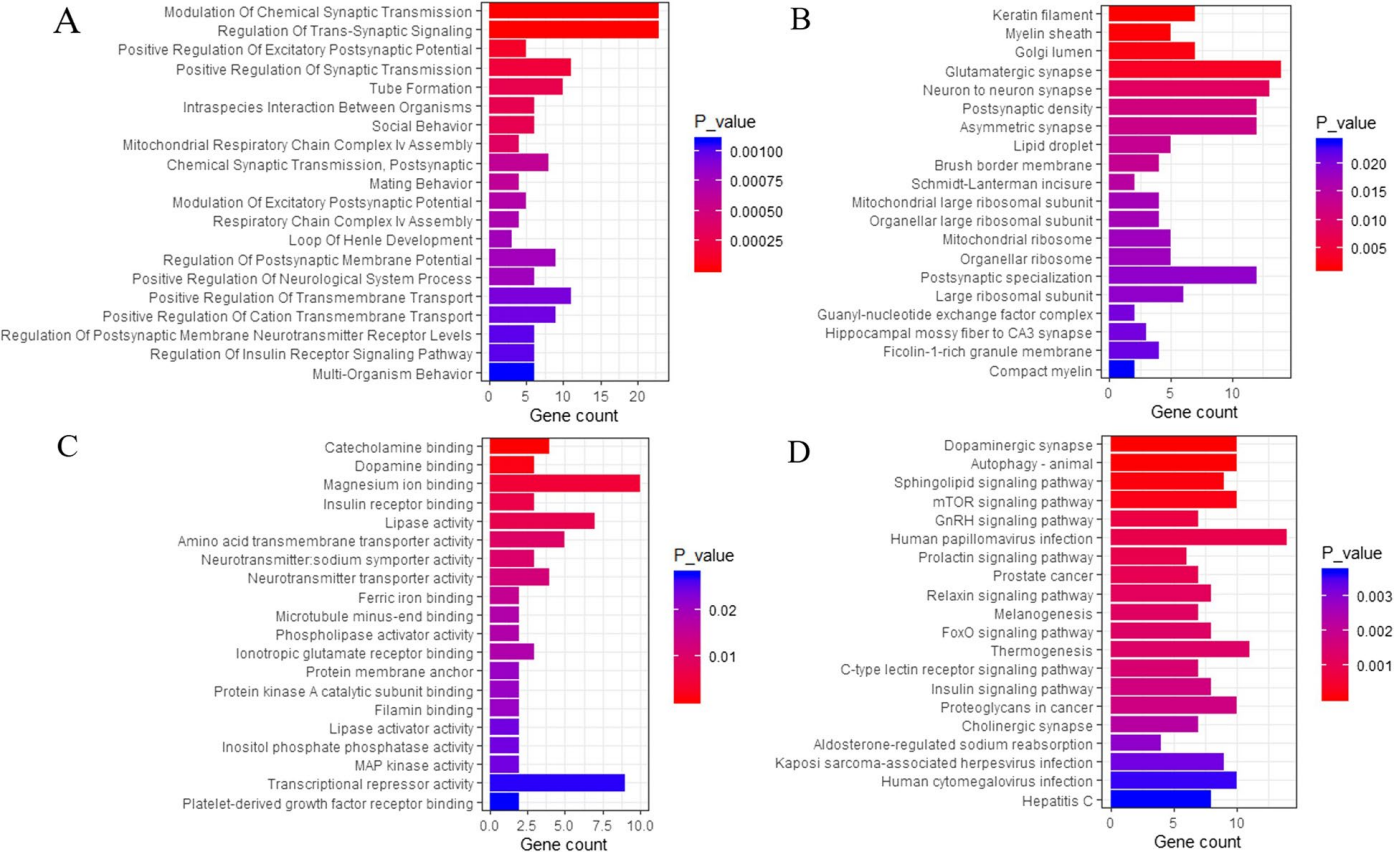
Function and pathway enrichment of the 511 genes from 22 potential pathogenic CNVs. Note: Top 20 annotations or pathways ordered by *P*_value. **A** Biological Process; **B** Cellular Component; **C** Molecular Function; **D** Kyoto encyclopedia of genes and genomes pathway. The ordinate represents the gene ontology function, and the abscissa represents the number of genes enriched to the term. *P*_value indicate the degree of enrichment, with smaller *P*_value indicating genes that are more likely to play significant functional roles

A total of 511 genes from the 22 potential pathogenic CNVs were functionally annotated by GO. The annotated genes were classified into three GO domains (biological processes [BP], cellular component [CC], and molecular function [MF]). For BP, some gene sets were enriched in synaptic-related functions, including modulation of chemical synaptic transmission (GO: 0050804), regulation of trans-synaptic signaling (GO: 0099177), positive regulation of excitatory postsynaptic potential (GO: 2000463), positive regulation of synaptic transmission (GO: 0050806), chemical postsynaptic transmission, (GO: 0099565), modulation of excitatory postsynaptic potential (GO: 0098815), and regulation of postsynaptic membrane potential (GO: 0060078), and in central nervous system related functions (positive regulation of neurological system process [GO: 0031646]). For CC, the top five CC terms included keratin filament (GO: 0045095), myelin sheath (GO: 0043209), Golgi lumen (GO: 0005796), glutamatergic synapse (GO: 0098978), and neuron to neuron synapse (GO: 0098984). For MF, the top five MF terms encompassed catecholamine binding (GO: 1901338), dopamine binding (GO: 0035240), magnesium ion binding (GO: 0000287), insulin receptor binding (GO: 0005158), and lipase activity (GO: 0016298). The top 20 GO functions are presented in Fig. 2 and Supplementary Tables 2, 3, and 4.

KEGG pathway enrichment analysis showed enriched key pathways, such as dopaminergic synapse (hsa04728), mTOR signaling pathway (hsa04150), insulin signaling pathway (hsa04910), and cholinergic synapse (hsa04725). The top 20 pathways are presented in Fig. 2 and Supplementary Table 5.

**Fig. 3:**
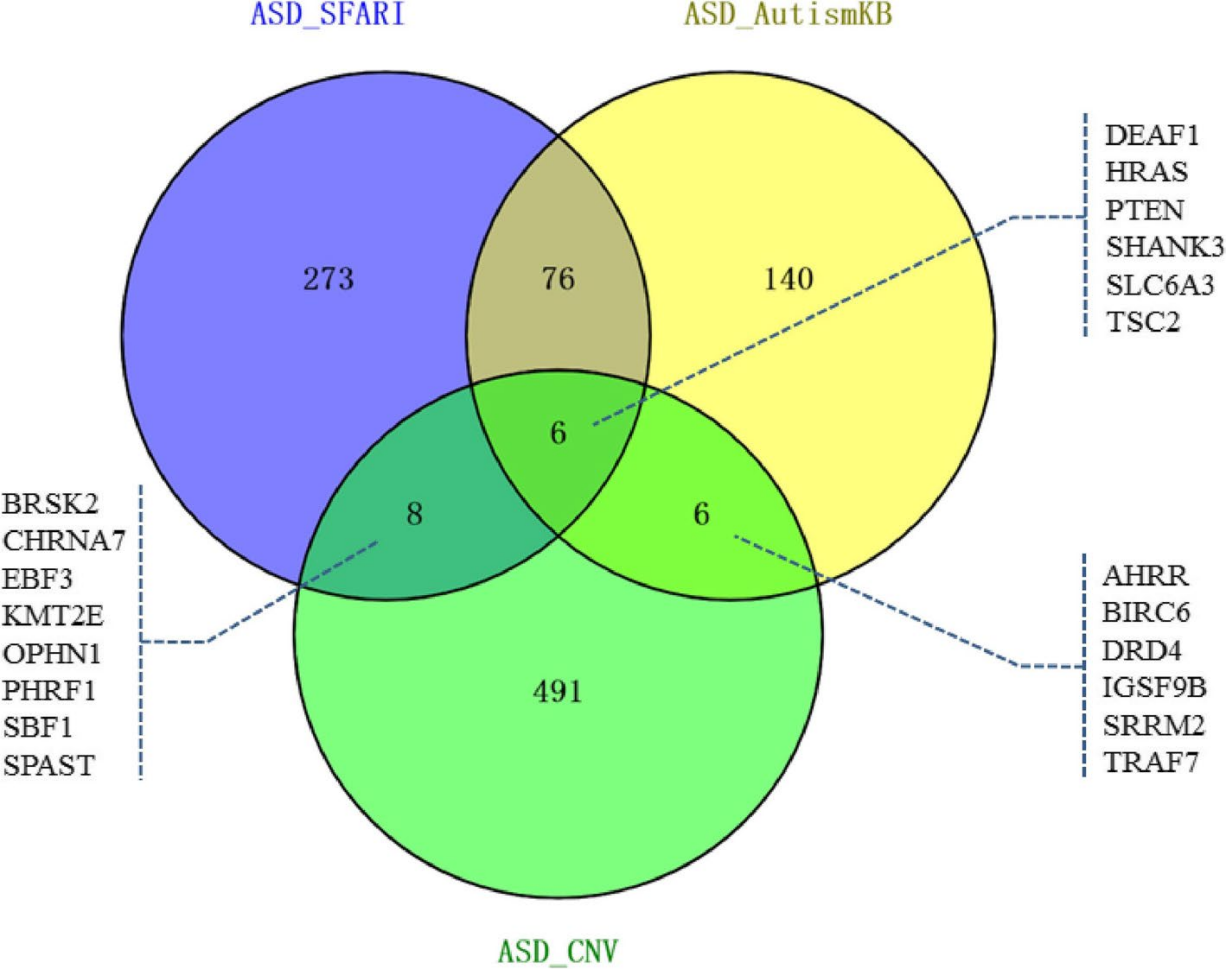
Venn diagram based on ASD_SFARI, ASD_AutismKB, and genes in our candidate CNVs for ASD. Note: We denote genes in our candidate CNVs for ASD as “ASD_CNV”, the 363 high confidence and strong candidate autism risk genes in SFARI as “ASD_SFARI”, and the 228 high confidence autism related genes in AutismKB as “ASD_AutismKB”

We constructed intersections among 511 genes that we found, 363 high-confidence or strong-candidate risk genes of ASD reported in SFARI database, and 228 high-confidence risk genes related to ASD reported in AutismKB database (Fig. 3). After investigating genes in the intersections, we found that cholinergic receptor nicotinic alpha 7 subunit gene (*CHRNA7*) was involved in the regulation of excitatory postsynaptic potential and cholinergic synapse; dopamine receptor D4 gene (*DRD4*) was involved in the regulation of synaptic transmission, dopamine binding, and glutamatergic synapse; HRas proto-oncogene (*HRAS*) played roles in the regulation of excitatory postsynaptic potential, glutamatergic synapse, and mTOR signal pathway; oligophrenin 1 gene (*OPHN1*) correlated with regulated synaptic signal, ionic glutamate receptor binding, and glutamatergic synapse; phosphatase and tensin homolog (*PTEN)* was implicated in the regulation of synaptic signal, neuron differentiation of central nervous system, ionic glutamate receptor binding, sphingolipid signaling, and mTOR signaling; SH3 and multiple ankyrin repeat domains 3 gene (*SHANK3*) was involved in the regulation of synaptic signal, ionic glutamate receptor binding, neuronal synapse, postsynaptic density, and asymmetric synapse; solute carrier family 6 member 3 gene (*SLC6A3*) played roles in dopamine binding, neurotransmitter: sodium cotransporter activity, and neurotransmitter transport activity; and TSC complex subunit 2 gene (*TSC2*) was involved in synapses, postsynaptic density, asymmetric synapses, and mTOR signaling pathways. Scores of all these genes (*CHRNA7*, *DRD4*, *HRAS*, *OPHN1*, *PTEN*, *SHANK3*, *SLC6A3*, and *TSC2*) in AustismKB and corresponding ranks in SFARI are listed in Table 4. *DRD4*, *HRAS*, *OPHN1*, *SHANK3*, *SLC6A3*, and *TSC2* were in the regions of CNVs duplication. *CHRNA7* and *PTEN* were in the regions of CNVs deletion.

**Table 4:** Summary of candidate genes of ASD

Gene Name	CNV Type	M/F No	Category of gene in SFARI	Score of gene in AutismKB
CHRNA7	Deletion	1/0	2	#
DRD4	Duplication	5/2	—	30
HRAS	Duplication	5/2	1	20
OPHN1	Duplication	1/0	2	#
PTEN	Deletion	13/0	1	78
SHANK3	Duplication	2/0	1	62
SLC6A3	Duplication	1/0	2	30
TSC2	Duplication	0/2	1	46

*M* Male, *F* Female. M/F No. means the number of CNV among male/female. —: Not reported as high-confidence or strong candidate autism risk genes in SFARI. #: Not reported as high-confidence autism risk genes in AutismKB

### Identification and analysis of potential pathogenic CNVs-encoded miRNAs of ASD

We found 50 potential-pathogenic-CNVs-encoded miRNAs (45 encoded by duplication regions and 5 encoded by deletion regions). According to experimental evidence documenting nervous system dysfunction, we retrieved PubMed, identifying that 12 CNVs-encoded miRNAs were previously reported to be associated with brain or nervous system dysfunction (Table 5).

**Table 5:** miRNAs with function related to brain or nervous system in CNVs

^miRNA ID^	CNV Type	^Functional relevance^	^Reference (PMID)^
miR-202	Duplication	Depression, Glioma, Neuroblastoma	32425535; 28714009; 21654684; 24337320
miR-210	Duplication	Alzheimer's disease, Epilepsy, Glioblastoma, Glioma, Head and neck paragangliomas, Neuroblastoma, Neuroprotective effects	31092279; 23108914; 21655185; 22977270; 23902947; 25279461; 24729345; 24382515; 25481483; 24930954; 25756397; 29126304; 29362886; 31146085; 32194691; 31896490; 29226333; 30947960; 30746749; 27471387
miR-3178	Duplication	Neuropsychiatric diseases	30766477
miR-339	Duplication	Alzheimer's disease, Glioblastoma, Neuroendocrine neoplasias	32176627; 29983867; 30564636; 24352696; 30176243
miR-4516	Duplication	Glioblastoma	30559405
miR-4717	Duplication	Guillain–Barre Syndrome	27836180
miR-483	Duplication	Alzheimer's disease, Glioma, Neuroblastoma	31938135; 24577456; 22465663
miR-675	Duplication	Glioma	31468534; 28187439; 24466011
miR-6821	Duplication	Alzheimer's disease	27050411
miR-940	Duplication	Glioblastoma, Glioma	31497204; 30906627; 31934283; 29296221; 30431124
miR-107	Deletion	Alzheimer's disease, Amnestic mild cognitive impairment, Bipolar disorder, Brain disorders, Frontotemporal dementia, Glioblastomas, Glioma, Major depression, Neuroblastoma, Neurogenesis, Schizophrenia	31556571; 29258209; 28847283; 26084601; 30543171; 31250578; 27343180; 21625387; 20489155; 28578378; 25662174; 22811466; 20413881; 31778666; 31787850; 29885309; 29671226; 30056425; 30480816; 18234899; 29136645; 23811124; 27143098; 29073742; 25596705; 31605836; 31420923; 23220650; 22594617; 26223576; 23572380; 27501295; 27878295; 32124921; 23962497; 29286086; 21179570; 21111402
miR-558	Deletion	Neuroblastoma	25616966; 27276678

**Fig. 4:**
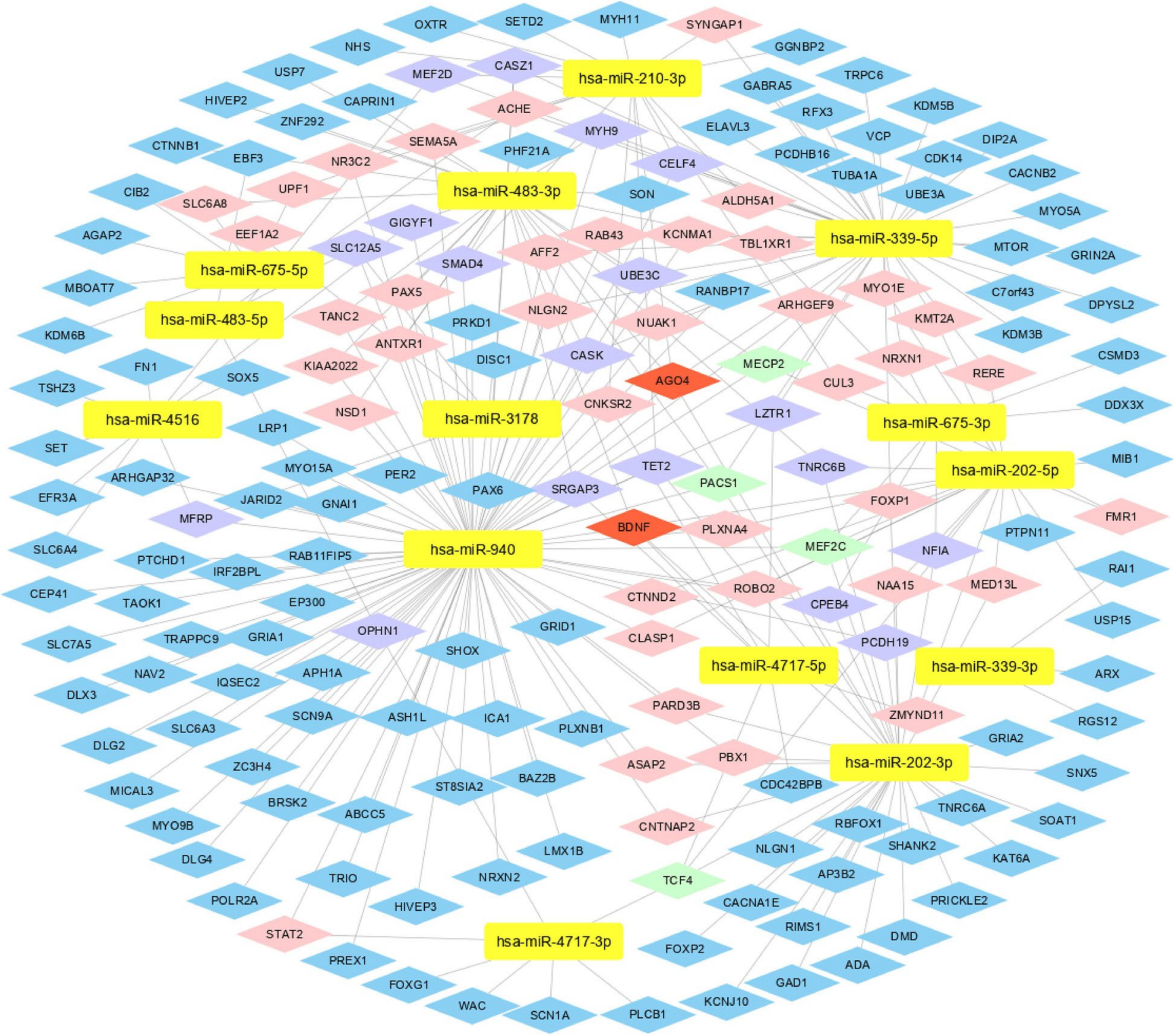
Interaction network of the CNVs-encoded-miRNAs-targeted genes in ASD (duplication). Note: Yellow rectangles represent the miRNAs encoded within pathogenic CNVs regions, while 219 CNVs-encoded-miRNAs-targeted genes are denoted by diamonds. Blue, pink, purple, light green, and red diamonds represent different target genes which are targeted by 1, 2, 3, 4, and 5 miRNAs respectively

**Fig. 5:**
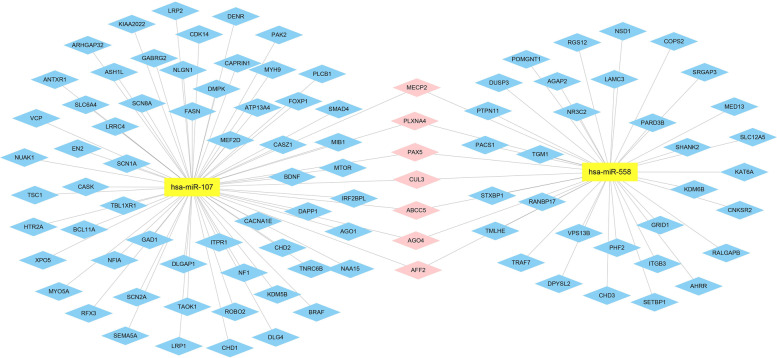
Interaction network of the CNVs-encoded-miRNAs-targeted genes in ASD (deletion). Note: Yellow rectangles represent the miRNAs encoded within pathogenic CNVs regions, while CNVs-encoded-miRNAs-targeted genes are denoted by diamonds. Blue and pink diamonds represent different target genes which are targeted by one and two CNVs-encoded miRNAs respectively

We intersected CNVs-encoded-miRNAs-targeted genes predicted using miRWalk 2.0 database with the union between SFARI and AutismKB (Supplementary Fig. 1). A total of 219 target genes were chosen for further study. We presented the interaction networks between CNVs-encoded miRNAs and 219 target genes (Figs. 4 and 5). The CNVs-encoded miRNAs and target genes are presented in Supplementary Tables 6 and 7.

**Fig. 6:**
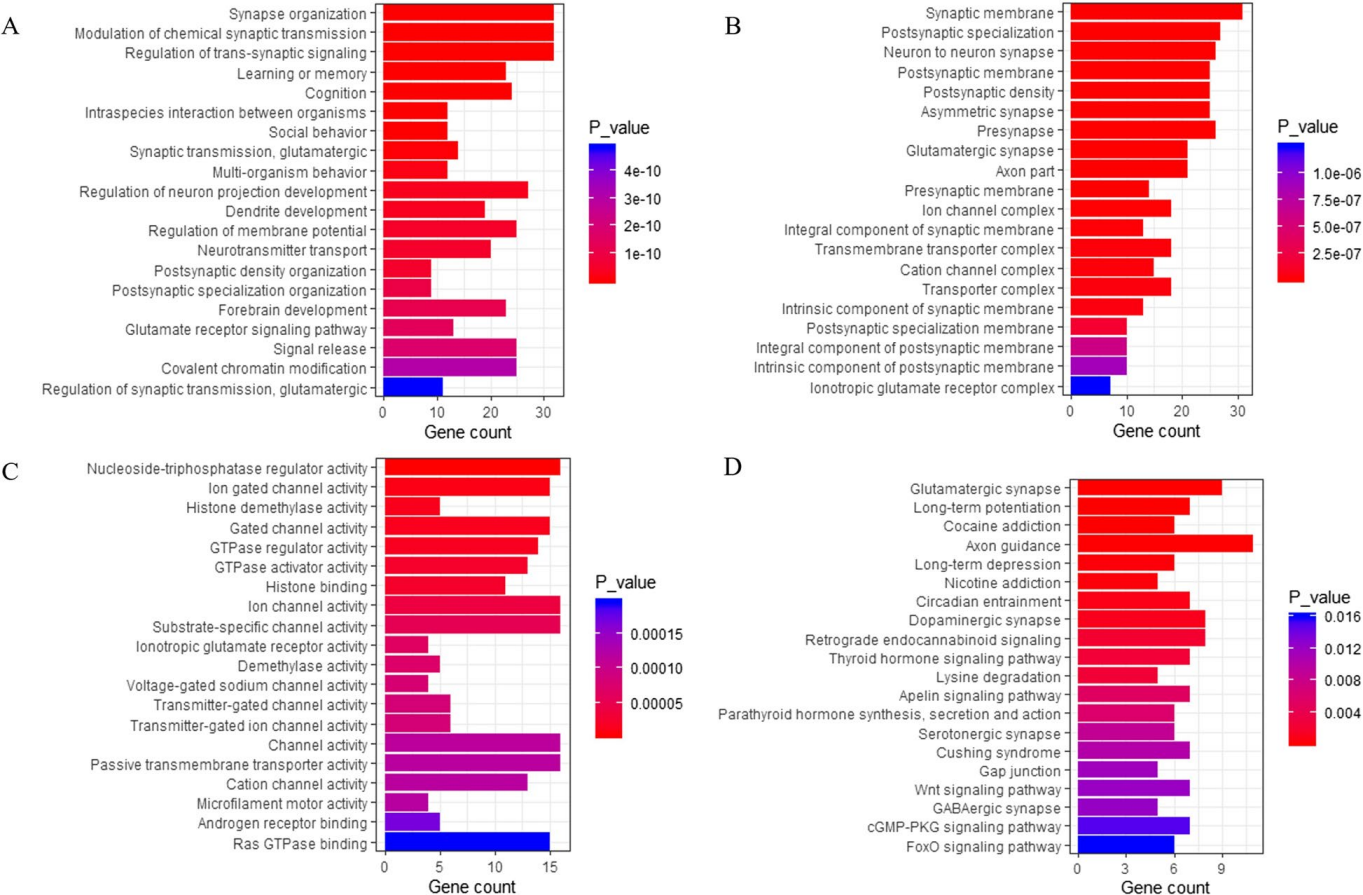
Function and pathway enrichment of the 219 CNVs-encoded-miRNAs-targeted genes of 16 potential pathogenic miRNAs encoded within CNVs. Note: Top 20 annotations or pathways ordered by *P*_value. **A** Biological Process; **B** Cellular Component; **C** Molecular Function; **D** Kyoto encyclopedia of genes and genomes pathway. The ordinate represents the gene ontology function, and the abscissa represents the number of genes enriched to the term. *P*_value indicate the degree of enrichment, with smaller *P*_value indicating genes that are more likely to play significant functional roles

We further investigated potential functions of the 219 target genes using GO analysis. For BP, some gene sets were enriched in synaptic-related functions, including synapse organization (GO: 0050808), modulation of chemical synaptic transmission (GO: 0050804), regulation of trans-synaptic signaling (GO: 0099177), synaptic transmission, glutamatergic (GO: 0035249), postsynaptic density organization (GO: 0097106), postsynaptic specialization organization (GO: 0099084) and regulation of glutamatergic synaptic transmission (GO: 0051966), and in central nervous system related functions, including learning or memory (GO: 0007611), cognition (GO: 0050890), and neurotransmitter transport (GO: 0006836). For CC, some gene sets were enriched in synaptic-related cellular components, including synapse membrane (GO: 0097060), postsynaptic specialization (GO: 0099572), and neuron to neuron synapse (GO: 0098984). For MF, some gene sets were enriched in ion-gated channel activity (GO: 0022839), gated channel activity (GO: 0022836), ion channel activity (GO: 0,005,216), ionotropic glutamate receptor activity (GO: 0004970), and transmitter gated channel activity (GO: 0022835). The top 20 GO functions are presented in Fig. 6 and Supplementary Tables 8, 9, and 10.

KEGG pathway enrichment analysis showed enriched key pathways, such as glutamatergic synapse (hsa04724), dopaminergic synapse (hsa04728), and Wnt signaling pathway (hsa04310). The top 20 pathways are presented in Fig. 6 and Supplementary Table 11.

## Discussion

In the present study, we identified that 22 kinds of CNVs (six deletions and 16 duplications), eight protein-coding genes, and 12 miRNAs-coding genes are associated with ASD risks in northeast Chinese Han from Jilin province, China.

CNVs have repeatedly been found to correlate with ASD risks [40, 41]. In our study, we filtered 22 potential pathogenic CNVs. Individuals with deletions and duplications of 15q13.3 have been found to manifest neuropsychiatric disease and cognitive deficits [42]. In line with the discoveries of Bitar et al*.* [43], Bremer et al*.* [44], Celestino-Soper et al*.* [45], Chen et al*.* [23], Chen et al*.* [46], Pinto et al*.* [28], and Rosenfeld et al*.* [47], we further documented that CNVs at 5p15.33, 5p15.33-p15.2, 7p22.3, 7p22.3-p22.2, 7q22.1-q22.2, 10q26.2-q26.3, 11q25, 12p12.1-p11.23, 15q13.3, 16p13.3, 22q13.31-q13.33, and Xq12-q13.1 were associated with ASD risks. Autism-related phenotypes are common in patients with deletion or duplication at 22q13.3 [48–51]. Most of the defects are due to haploinsufficiency of *SHANK3* [49]. Chen et al*.* found a deletion at 22q13.3 in two male children with ASD and a duplication at 22q13.31-q13.33 in one male child with ASD from Taiwan, China [46]. In our study, we found a duplication at 22q13.31-q13.33 that overlaps *SHANK3* from two male children with ASD, indicating that the duplication at 22q13.31-q13.33 may play a key role in ASD etiology in our population. CNVs at 15q13.3 have been found to be involved in a variety of neuropsychiatric diseases, including intellectual disability/developmental delay, epilepsy, schizophrenia, and ASD [42, 52–54]. The relation between *CHRNA7* at 15q13.3 and neuropsychiatric disorder phenotype has been validated intensively [53]. In accordance with the discovery of Pinto et al*.* [28], we also found that a deletion of *CHRNA7* was associated with ASD risks.

Except *CHRNA7* and *SHANK3*, we found CNVs-duplications (*DRD4*, *HRAS*, *OPHN1*, *SLC6A3*, and *TSC2*) and CNVs-deletions (*PTEN*). For *DRD4* and *HARS*, we found seven children with ASD had duplications at 11p15.5, which overlaps *DRD4* and *HARS*. Mutations in *DRD4* are associated with ASD risks [55–57]. The mRNA expression levels of *DRD4* in peripheral blood lymphocytes are higher in people with ASD than those in healthy controls [58, 59]. Herault et al*.* also found positive association between *HRAS* and autism in French-Caucasian [60, 61]. For *OPHN1* at Xq12-q13.1, Celestino-Soper et al*.* found a deletion of exons 7–15 of *OPHN1* at Xq12 in a male child with ASD [45]. In contrast, we found a male child with ASD had a duplication at Xq12-q13.1. For *SLC6A3* at 5p15.33-p15.2, Bowton et al*.* found *SLC6A3* coding variant Ala559Val is related to ASD [62]. We further found a child with ASD had a duplication at 5p15.33-p15.2. For *TSC2* at 16p13.3 and *PTEN* at 10q23.2-q23.31, Bourgeron et al*.* found that mutations in *TSC2* and *PTEN* activate the mTOR/PI3K pathway, associating with ASD risks [63]. We found duplications at 16p13.3 in two female children with ASD. PTEN loss involved in white matter pathology in human with ASD is consistent with that in mouse models [64]. We revealed that deletions at 10q23.2-q23.31 overlapping *PTEN* in 13 male children with ASD, rather than 3 female children with ASD. Thus, these eight genes may be implicated in ASD etiology.

MiRNAs encoded within CNVs are important functional variants, providing a new dimension to recognize the association between genotype and phenotype [65]. MiRNAs play vital roles in governing essential aspects of inhibitory transmission and interneuron development in nervous system [66]. Deletion or duplication of a chromosomal loci changes the levels of miRNAs which further impact on neuronal function and communication [36]. In our study, 12 candidate-susceptible miRNAs-coding genes of ASD were identified (ten duplications [*MIR202*, *MIR210*, *MIR3178*, *MIR339*, *MIR4516*, *MIR4717*, *MIR483*, *MIR675*, *MIR6821*, and *MIR940*] and two deletions [*MIR107* and *MIR558*]). *BDNF*, a brain-derived neurotrophic factor and a member of the neurotrophic factor family, is a target gene of miR-202 [67]. Moreover, we further predicted that miR-4717-5p, miR-483-3p, and miR-940 also targeted *BNDF*. Skogstrand et al*.* found that lower BDNF levels in serum correlate with ASD risks [68, 69]. miR-339-5p has been found to be a drug target for Alzheimer's disease, and is low expressed in mature neurons and related to axon guidance [70, 71]. In our study, we found that miR-339-5p targets 42 genes associated with ASD risks. Among these genes, the association of *DIP2A* and ASD risks has been validated by our team [72]; moreover, *Dip2a* knockout mice exhibit autism-like behaviors, including excessive repetitive behavior and social novelty defects [73]. Notably, autism-like behaviors and germline transmission in *MECP2* transgenic monkeys corroborate association between miR-339-5p and *MECP2* [74]. In addition, miR-202-5p, miR-483-3p, and miR-940 also targets *MECP2*. For these reasons, miRNAs encoded within CNVs may be implicated in ASD etiology.

For enrichment analysis, we found that genes were enriched in synapse, synapse-related signal regulation, neurotransmitter activity, neurotransmitter transport, and neurotransmitter binding. Mutations in synapse-related or neurotransmitter-related genes are associated with ASD risks in multiple unbiased, targeted sequencing, and neuropathological studies, evidencing that dysregulation in synaptogenesis and neurotransmission is implicated in the pathogenesis of ASD [75–78]. We corroborated that ASD pathogenesis was related to dopaminergic synapse, mTOR signaling pathway, insulin signaling pathway, and cholinergic synapse [79–82]. Dopamine affects ASD-related-brain regions (basal ganglia, cortex, and amygdala) via dopaminergic synapse [79]. mTOR is involved in integrating signaling from ASD synaptic and regulatory proteins, such as SHANK3, FMRP and the glutamate receptors mGluR1/5 [63, 83]. Dysfunction in mTOR signaling affords one of mechanisms of ASD — an imbalance between excitatory and inhibitory currents [80]. Insulin signaling pathway is feasible for development of autism [81]. Neurochemical abnormalities in the cholinergic system are involved in ASD pathogenesis, highlighting the potential for intervention-targeted cholinergic synapses [82].

Functional network analysis of the 219 CNVs-encoded-miRNAs-targeted genes elicited that a novel regulating mechanism of these CNVs-encoded miRNAs consisted of synapse-related functions (glutamatergic synapse, dopaminergic synapse, serotonergic synapse, and GABAergic synapse), axon guidance, ion channel (ion-gated channel and cation channel complex), and Wnt signaling pathway. Synaptic function and Wnt signaling pathway are affected by mutations in diverse ASD-related genes, and altered Wnt pathway signaling may confer an involvement in ASD pathogenesis [78]. Interestingly, dysfunction of axon-guidance signaling is integral to the microstructural abnormalities of the brain in people with ASD [84]. Notably, the involvement of ion channel-related genes has been found in ASD etiology [85]. Mutations in ion channel genes contribute to low-to-moderate susceptibility of ASD [85].

Both GO and Pathway enrichment analyses showed that CNVs-relating genes and CNVs-encoded-miRNAs-targeted genes mapped synapse-related functions. Additionally, CNVs-relating genes also enriched in mTOR signaling pathway and insulin signaling pathway. In contrast, CNVs-encoded-miRNAs-targeted genes enriched in axon guidance, ion channel, and Wnt signaling pathway. These results documented the high complexity and heterogeneity of ASD, suggesting that different genomic alteration on same chromosomal location may confer distinct but complementary effects on the brain of people with ASD.

Our study had some limitations: (1) the sample size in our study may confer limited statistical power to discover significant findings; (2) genetic and environmental factors contribute to ASD risk; however, environmental factors were not available for us; and (3) de novo or inherited of the CNVs were not be classified because of the lack of data from parents.

Despite these limitations above, our study also had some strength. Firstly, we found eight de novo CNVs (duplications at 1p36.31, 1p36.33, 1q42.13, 11p15.5, and 16q21; deletions at 2p23.1-p22.3, 10q23.2-q23.31, and 14q11.2) and 12 validated CNVs (duplication at 5p15.33, 5p15.33-p15.2, 7p22.3, 7p22.3-p22.2, 10q26.2-q26.3, 11q25, 16p13.3, 22q13.31-q13.33, and Xq12-q13.1; deletion at 7q22.1-q22.2, 12p12.1-p11.23, and 15q13.3), further documenting that ASD is of high genetic heterogeneity after comparing our results and previous findings (Supplementary Table 12). Secondly, we identified 20 genes (eight protein-coding genes supported by SFARI and AutismKB and 12 microRNAs-coding genes that refine understanding of involving approach of ASD-susceptible-genes in etiology) are implicated in ASD risks. Thirdly, we performed GO and KEGG pathway analyses of CNVs-relating genes and CNVs-encoded-miRNAs-targeted genes, providing a new dimension to revealing ASD etiology.

## Conclusions

In summary, we identified that 22 kinds of CNVs (six deletions and 16 duplications), eight protein-coding genes, and 12 miRNAs-coding genes are implicated in ASD risks, conferring perception to further reveal ASD etiology.

## Supplementary Information

**Additional file 1. Supplementary Table 1.** Summary of potential Pathogenic CNVs of ASD. **Supplementary Table 2.** Functional annotation (biological processes) of the 511 genes from 22 potential pathogenic CNVs (top 20). **Supplementary Table 3.** Functional annotation (cellular components) of the 511 genes from 22 potential pathogenic CNVs (top 20). **Supplementary Table 4.** Functional annotation (molecular function) of the 511 genes from 22 potential pathogenic CNVs (top 20). **Supplementary Table 5.** Pathway enrichment of the 511 genes from 22 potential pathogenic CNVs (top 20). **Supplementary Table 6.** CNVs-encoded-miRNAs-target genes of ASD (duplication). **Supplementary Table 7.** CNVs-encoded-miRNAs-target genes of ASD (deletion). **Supplementary Table 8.** Functional annotation (biological processes) of the 219 target genes of potential pathogenic miRNAs coded within CNVs (top 20). **Supplementary Table 9.** Functional annotation (cellular components) of the 219 target genes of potential pathogenic miRNAs coded within CNVs (top 20). **Supplementary Table 10.** Functional annotation (molecular function) of the 219 target genes of potential pathogenic miRNAs coded within CNVs (top 20). **Supplementary Table 11.** Pathway enrichment of the 219 target genes of potential pathogenic miRNAs coded within CNVs (top 20). **Supplementary Table 12.** Comparison of CNVs involved in ASD. **Supplementary Fig. 1.** Venn diagram based on ASD_SFARI, ASD_AutismKB, and CNVs-encoded-miRNAs-targeted genes.

## Data Availability

The datasets used during the current study are available from the corresponding author on reasonable request.

## Abbreviations

ASD: Autism spectrum disorder
CNV: Copy number variation
Kb: Kilobase
aCGH: Array-based comparative genomic hybridization
miRNAs: MicroRNAs
GO: Gene Ontology
BP: Biological processes
CC: Cellular component
MF: Molecular function
