# Supplementary material for: Nexus between genome-wide copy number variations and autism spectrum disorder in Northeast Han Chinese population
Source: BMC Psychiatry. 2023 Feb 7;23:96. doi: 10.1186/s12888-023-04565-7 (PMC9906952; doi:10.1186/s12888-023-04565-7)
Supplement: Supplementary file 1 — Additional file 1. Supplementary Table 1. Summary of potential Pathogenic CNVs of ASD. Supplementary Table 2. Functional annotation (biological processes) of the 511 genes from 22 potential pathogenic CNVs (top 20). Supplementary Table 3. Functional annotation (cellular components) of the 511 genes from 22 potential pathogenic CNVs (top 20). Supplementary Table 4. Functional annotation (molecular function) of the 511 genes from 22 potential pathogenic CNVs (top 20). Supplementary Table 5. Pathway enrichment of the 511 genes from 22 potential pathogenic CNVs (top 20). Supplementary Table 6. CNVs-encoded-miRNAs-target genes of ASD (duplication). Supplementary Table 7. CNVs-encoded-miRNAs-target genes of ASD (deletion). Supplementary Table 8. Functional annotation (biological processes) of the 219 target genes of potential pathogenic miRNAs coded within CNVs (top 20). Supplementary Table 9. Functional annotation (cellular components) of the 219 target genes of potential pathogenic miRNAs coded within CNVs (top 20). Supplementary Table 10. Functional annotation (molecular function) of the 219 target genes of potential pathogenic miRNAs coded within CNVs (top 20). Supplementary Table 11. Pathway enrichment of the 219 target genes of potential pathogenic miRNAs coded within CNVs (top 20). Supplementary Table 12. Comparison of CNVs involved in ASD. Supplementary Fig. 1. Venn diagram based on ASD_SFARI, ASD_AutismKB, and CNVs-encoded-miRNAs-targeted genes. [file 12888_2023_4565_MOESM1_ESM.docx]

Supplementary Table 1–12 and Supplementary Fig. 1

| Supplementary Table 1. Summary of potential Pathogenic CNVs of ASD | | | | | | | |  |
| --- | --- | --- | --- | --- | --- | --- | --- | --- |
| Sample ID | M/F No. | Classification^*^ | Coordinates, hg18 | Cytoband | Size (Kb) | CNV Type | Gene Name |  |
| 1 | 1/0 | LP | chr1:1179223-2271500 | 1p36.33 | 1092.277 | Dup | SCNN1D, CPSF3L, TAS1R3, DVL1, AURKAIP1, CCNL2, MRPL20, VWA1, ATAD3B, ATAD3A, TMEM240, MIB2, MMP23B, MMP23A, CDK11B, CDK11A, NADK, GNB1, CALML6, GABRD, PRKCZ, SKI,UBE2J2, ACAP3, MIR6726, PUSL1, MIR6727, CPTP, MIR6808, MXRA8, LOC148413, ANKRD65, TMEM88B, LOC102724312, ATAD3C, SSU72, C1orf233, SLC35E2B, SLC35E2, TMEM52, CFAP74, FAAP20 | |
| 1/3 | 2/0 | LP | chr1:2271500-2386916 | 1p36.33-p36.32 | 115.416 | Dup | MORN1, LOC100129534, RER1 | |
| 1/3/5/7 | 4/0 | - | chr1:2386916-4858418 | 1p36.32 | 2471.502 | Dup | PLCH2, PANK4, HES5, TNFRSF14, ACTRT2, PRDM16, MEGF6, MIR551A, TPRG1L, TP73, SMIM1, CEP104, DFFB, AJAP1, LOC100996583, LOC115110, FAM213B, MMEL1, TTC34, LINC00982, MIR4251, ARHGEF16, WRAP73, TP73-AS1, CCDC27, LRRC47, C1orf174, LINC01134, LINC01346, LOC284661 | |
| 1/3/5 | 3/0 | - | chr1:4858418-5788004 | 1p36.32-p36.31 | 929.586 | Dup | MIR4417 | |
| 1/3 | 2/0 | LP | chr1:5788004-5998727 | 1p36.31 | 210.723 | Dup | NPHP4, KCNAB2, MIR4689 | |
| 3 | 1/0 | LP | chr1:5998727-6334157 | 1p36.31 | 335.430 | Dup | CHD5, RPL22, ICMT, HES3, GPR153, ACOT7, LOC102724450, RNF207, LINC00337 | |
| 16 | 1/0 | LP | chr1:150651299-150852905 | 1q21.3 | 201.606 | Dup | LCE5A, LCE3E, LCE3D, LCE3C, CRNN, CRCT1 | |
| 6/7/8/15 | 4/0 | LP | chr1:225876894-226738916 | 1q42.13 | 862.022 | Dup | WNT3A, ARF1, MRPL55, GUK1, GJC2, IBA57, TRIM11, HIST3H3, HIST3H2A, HIST3H2BB, ZNF678, ZNF847P, LOC100130093, JMJD4, SNAP47, PRSS38, WNT9A, MIR5008, MIR3620, C1orf35, IBA57-AS1, C1orf145, OBSCN, MIR6742, TRIM17, MIR4666A | |
| 7/15 | 2/0 | LP | chr1:226738916-227135170 | 1q42.13 | 396.254 | Dup | RNF187, BTNL10, MIR7641-2, DUSP5P1 | |
| 8 | 1/0 | P | chr2:31412158-32712484 | 2p23.1-p22.3 | 1300.327 | Del | XDH, SRD5A2, MEMO1, DPY30, SPAST, SLC30A6, NLRC4, BIRC6, MIR558, YIPF4, BIRC6-AS2, TTC27 | |
| 4/14 | 0/2 | LP | chr2:88924973-89057332 | 2p11.2 | 132.360 | Dup |  | |
| 13 | 1/0 | LB | chr2:106184290-107769684 | 2q12.2-q12.3 | 1585.395 | Dup | PLGLA, RGPD3, ST6GAL2, MIR548AU, GACAT1 | |
| 15 | 1/0 | P | chr2:219857472-222805232 | 2q35-q36.1 | 2947.761 | Dup | DNAJB2, PTPRN, RESP18, DNPEP, DES, SPEG, GMPPA, ASIC4, CHPF, OBSL1, INHA, STK11IP, PAX3, MIR153-1, LOC100996693, TMEM198, MIR3132, SLC4A3, MIR4268, EPHA4 | |
| 10 | 1/0 | LB | chr2:234842880-236353765 | 2q37.1-q37.2 | 1510.885 | Dup | ARL4C, SH3BP4, LINC01173, AGAP1, AGAP1-IT1 | |
| 3/10 | 2/0 | LB | chr2:236353765-236504477 | 2q37.2 | 150.712 | Dup | AGAP1 | |
| 3/6 | 2/0 | LP | chr3:3862102-3944067 | 3p26.2 | 81.966 | Del | LRRN1 | |
| 12 | 0/1 | LP | chr3:81874379-83271792 | 3p12.2 | 1397.414 | Dup | GBE1 | |
| 7 | 2/0 | P | chr3:185392161-185852438 | 3q27.1 | 460.278 | Dup | MIR1224, ALG3, ECE2, CAMK2N2, PSMD2, EIF4G1, CLCN2, POLR2H, THPO, CHRD, EPHB3, ABCF3, VWA5B2, SNORD66, FAM131A, EIF2B5-AS1 | |
| 15 | 1/0 | LP | chr3:195728212-196943436 | 3q29 | 1215.225 | Del | LSG1, XXYLT1, PPP1R2, APOD, MIR570, MUC20, TMEM44-AS1, TMEM44, FAM43A, LOC100507391, XXYLT1-AS1, MIR3137, XXYLT1-AS2, ACAP2, MIR5692C1, SDHAP2, LINC00969 | |
| 8 | 1/0 | LB | chr4:68511739-68595643 | 4q13.2 | 83.905 | Del | TMPRSS11A, TMPRSS11GP | |
| 2 | 1/0 | LP | chr5:32098707-32209107 | 5p13.3 | 110.401 | Del | PDZD2, GOLPH3 | |
| 1 | 1/0 | LP | chr5:360041-873365 | 5p15.33 | 513.324 | Dup | PDCD6, AHRR, EXOC3, SLC9A3, CEP72, TPPP, EXOC3-AS1, PP7080, LOC100288152, MIR4456, LOC100996325 | |
| 1/12 | 1/1 | LP | chr5:873365-1115468 | 5p15.33 | 242.103 | Dup | TRIP13, NKD2, SLC12A7, ZDHHC11, BRD9, LOC100506688 | |
| 1 | 1/0 | P | chr5:1115468-8452427 | 5p15.33-p15.2 | 7336.959 | Dup | SLC6A19,  SLC6A18, TERT, CLPTM1L, SLC6A3, LPCAT1, MRPL36, NDUFS6, IRX4, IRX2, IRX1,  ADAMTS16, MED10, UBE2QL1, LINC01018, NSUN2, SRD5A1, ADCY2, MTRR,  MIR4635, CTD-3080P12.3, MIR4457, LINC01511, MIR6075, SDHAP3, LOC728613, MIR4277, LOC101929034, CTD-2194D22.4, LOC100506858, C5orf38, LINC01377, LINC01019, LINC01017, LOC101929153, LINC01020, LOC105374631, CTD-2297D10.2, ICE1, FLJ33360, LOC100505625, PAPD7, MIR4278, MIR4454, LOC442132, LOC101929261, C5orf49, FASTKD3, LOC729506 | |
| 1 | 1/0 | LP | chr6:29870108-30053413 | 6p22.1-p21.33 | 183.306 | Del | HLA-G, HLA-H, HLA-A, HCG9, LOC554223, HCG4B | |
| 16 | 1/0 | LP | chr6:91060808-95641411 | 6q15-q16.1 | 4580.604 | Dup | BACH2, MAP3K7, EPHA7, MIR4464, MIR4643, CASC6, TSG1 | |
| 6 | 1/0 | LB | chr6:160247865-160248712 | 6q25.3 | 0.848 | Del | MAS1 | |
| 16 | 1/0 | LP | chr6:168205934-169332956 | 6q27 | 1127.023 | Dup | DACT2, SMOC2, FRMD1, LOC105378137, LOC101929420, LOC101929460, LOC102724357, LINC01615, LOC101929504 | |
| 7 | 1/0 | - | chr7:322848-524935 | 7p22.3 | 202.087 | Dup | PDGFA | |
| 7/8 | 2/0 | LP | chr7:524935-1037461 | 7p22.3 | 512.526 | Dup | PDGFA, PRKAR1B, GET4, COX19, CYP2W1, MIR339, HRAT92, LOC101927000, LOC101926963, DNAAF5, SUN1, ADAP1, C7orf50 | |
| 7 | 1/0 | LP | chr7:1037461-2536804 | 7p22.3-p22.2 | 1499.343 | Dup | ZFAND2A, INTS1, MAFK, ELFN1, MAD1L1, FTSJ2, NUDT1, SNX8, EIF3B, CHST12, LFNG,GPR146, GPER1, LOC101927021, UNCX, MICALL2, TMEM184A, PSMG3, PSMG3-AS1, TFAMP1, ELFN1-AS1, MIR4655, MIR6836, LOC101927181, GRIFIN, MIR4648 | |
| 6 | 1/0 | LP | chr7:103622888-104803388 | 7q22.1-q22.2 | 1180.501 | Del | ORC5, LHFPL3, KMT2E, LHFPL3-AS1, LHFPL3-AS2, LINC01004, KMT2E-AS1, SRPK2 | |
| 5/9/15 | 3/0 | LB | chr7:148832718-148911412 | 7q36.1 | 78.695 | Del | ZNF767P | |
| 4/12/14 | 0/3 | B | chr8:39378051-39505315 | 8p11.23-p11.22 | 127.265 | Dup | ADAM5, ADAM3A | |
| 5/7 | 2/0 | B | chr8:39378051-39505315 | 8p11.23-p11.22 | 127.265 | Del | ADAM5, ADAM3A | |
| 11 | 1/0 | - | chr10:45376528-46431590 | 10q11.21-q11.22 | 1055.062 | Del | MARCH8, FAM21C, SYT15, GPRIN2 | |
| 9/11 | 2/0 | - | chr10:46431590-47125152 | 10q11.22 | 693.563 | Del | NPY4R, ANXA8, CH17-360D5.1, LINC00842, HNRNPA1P33, FAM25C, FAM25G, AGAP9, BMS1P6, FAM35DP, ANTXRLP1 | |
| 2/3/9/11/13 | 5/0 | LP | chr10:88843151-88854843 | 10q23.2-q23.31 | 11.692 | Del | GLUD1 | |
| 1/2/3/6/8/9/10/11/13/15/16 | 11/0 | LP | chr10:88854843-89510992 | 10q23.2-q23.31 | 656.149 | Del | MINPP1, PAPSS2 | |
| 1/2/3/5/6/7/8/9/10/11/13/15/16 | 13/0 | LP | chr10:89540133-91524263 | 10q23.2-q23.31 | 1984.131 | Del | ATAD1, KLLN, PTEN, RNLS, LIPJ, LIPK, LIPN, LIPM, STAMBPL1, ACTA2, FAS, CH25H, LIPA, IFIT2, IFIT1, IFIT5, SLC16A12, PANK1, MIR107, CFL1P1, LIPF, ANKRD22, ACTA2-AS1, FAS-AS1, MIR4679-2,  MIR4679-1, IFIT3, IFIT1B, SLC16A12-AS1, FLJ37201, KIF20B | |
| 7 | 1/0 | P | chr10:127658856-135254513 | 10q26.2-q26.3 | 7595.658 | Dup | FANK1, ADAM12, DOCK1, NPS, PTPRE, MKI67, MGMT, EBF3, GLRX3, PPP2R2D, BNIP3, JAKMIP3, DPYSL4, INPP5A, KNDC1, UTF1, VENTX, ADAM8, CALY, PRAP1, ECHS1, PAOX, SPRN, CYP2E1, SYCE1, LINC00601, C10orf90, FAM196A, FOXI2, CLRN3, LINC01163, MIR4297, LINC00959, CTAGE7P, MIR378C, TCERG1L, TCERG1L-AS1, LINC01164, STK32C, LRRC27, PWWP2B, C10orf91, NKX6-2, CFAP46, LINC01166, LINC01167, LINC01168, ADGRA1-AS1, ADGRA1, MIR202HG, MIR202, TUBGCP2, ZNF511, FUOM, MIR3944, MTG1, SCART1, SPRNP1 | |
| 4 | 0/1 | LP | chr11:208365-274838 | 11p15.5 | 66.473 | Dup | SIRT3, PSMD13 | |
| 1/4/5/14 | 2/2 | LP | chr11:274838-303837 | 11p15.5 | 28.999 | Dup | IFITM5, IFITM2 | |
| 1/4/5/7/14 | 3/2 | LP | chr11:303837-393800 | 11p15.5 | 89.963 | Dup | IFITM1, IFITM3 | |
| 1/4/5/7/14/15 | 4/2 | LP | chr11:393800-498019 | 11p15.5 | 104.219 | Dup | PKP3, SIGIRR, PTDSS2, RNH1 | |
| 1/4/5/7/8/14/15 | 5/2 | P | chr11:498019-2179368 | 11p15.5 | 1681.349 | Dup | HRAS, RASSF7, MIR210, IRF7, CDHR5, SCT, DRD4, DEAF1, EPS8L2, TALDO1, CEND1, SLC25A22, PIDD1, RPLP2, PNPLA2, CD151, POLR2L, TSPAN4, CHID1, AP2A2, MUC6, MUC2, MUC5AC, MUC5B, TOLLIP, BRSK2, DUSP8, KRTAP5-1, CTSD, SYT8, TNNI2, LSP1, TNNT3, H19, MIR675, IGF2, INS, TH, ASCL2, C11orf21, TSPAN32, CD81, TSSC4, TRPM5, KCNQ1, KCNQ1OT1, KCNQ1DN, CDKN1C, PHLDA2, LRRC56, LMNTD2, MIR210HG, LOC143666, PHRF1, TMEM80, PDDC1, LOC171391, PANO1, SNORA52, CRACR2B, MIR6744, TOLLIP-AS1, MOB2, KRTAP5-AS1, KRTAP5-2, KRTAP5-3, KRTAP5-4, KRTAP5-5, FAM99A, FAM99B, KRTAP5-6, IFITM10, MIR4298, MIR7847, LINC01150, MRPL23, SNORD131, MRPL23-AS1, LINC01219, HOTS, INS-IGF2, MIR483, IGF2-AS, MIR4686 | |
| 1/4/7/8/14/15 | 4/2 | P | chr11:2179368-2888221 | 11p15.4 | 708.853 | Dup | CD81-AS1, KCNQ1-AS1, SLC22A18AS, SLC22A18 | |
| 4/14/15 | 1/2 | LP | chr11:2906298-2941885 | 11p15.4 | 35.587 | Dup | SNORA54 | |
| 4 | 0/1 | LB | chr11:3030264-3617261 | 11p15.4 | 586.997 | Dup | MRGPRG-AS1, OR7E12P, LOC101927708, TRPC2 | |
| 16 | 1/0 | LP | chr11:68323254-69629531 | 11q13.2-q13.3 | 1306.278 | Dup | CPT1A, MRPL21, IGHMBP2, MRGPRD, MRGPRF, TPCN2, MYEOV, CCND1, ORAOV1, FGF19, FGF4, FGF3, ANO1, MRGPRF-AS1, MIR3164, LOC338694, LOC102724265, LINC01488, LOC101928443, -AS2, ANO1 | |
| 1 | 1/0 | LP | chr11:78077498-78670982 | 11q14.1 | 593.485 | Dup | TENM4 | |
| 1/10/15 | 3/0 | LB | chr11:130260489-130772681 | 11q24.3-q25 | 512.192 | Dup | SNX19 | |
| 1/3/10/15 | 4/0 | LB | chr11:130772681-131207591 | 11q25 | 434.910 | Dup | NTM, NTM-AS1, LOC101929653 | |
| 1/3/9/10/15 | 5/0 | VOUS | chr11:131207591-132514983 | 11q25 | 1307.393 | Dup | NTM, OPCML, NTM-IT | |
| 1/3/10/15 | 4/0 | VOUS | chr11:132514983-132773688 | 11q25 | 258.705 | Dup | OPCML | |
| 1/3/15 | 3/0 | LP | chr11:132773688-134043707 | 11q25 | 1270.019 | Dup | OPCML, SPATA19, IGSF9B, JAM3, NCAPD3, VPS26B, THYN1, ACAD8, B3GAT1, LOC646522, MIR4697HG, MIR4697, LOC100128239, GLB1L3, GLB1L2, LOC283177 | |
| 1/3 | 2/0 | LP | chr11:134043707-134251838 | 11q25 | 208.131 | Dup |  | |
| 1 | 1/0 | LB | chr11:134251838-134373617 | 11q25 | 121.779 | Dup | LOC100507548 | |
| 10 | 1/0 | LP | chr12:12247496-12510475 | 12p13.2 | 262.980 | Del | LRP6, BORCS5, MANSC1, LOH12CR2 | |
| 11 | 1/0 | LP | chr12:25048911-25133934 | 12p12.1 | 85.023 | Del |  | |
| 5/9/11 | 3/0 | LP | chr12:25133934-25156062 | 12p12.1 | 22.128 | Del | LRMP | |
| 1/2/5/9/11/13 | 6/0 | LP | chr12:25156062-27414420 | 12p12.1-p11.23 | 2258.359 | Del | KRAS, RASSF8, BHLHE41, SSPN, ITPR2, ASUN, TM7SF3, MED21, STK38L, ARNTL2, CASC1, LYRM5, LMNTD1, MIR4302, RASSF8-AS1, FGFR1OP2, C12orf71 | |
| 9/11/13 | 3/0 | B | chr12:27414420-27464769 | 12p11.22 | 50.349 | Del | ARNTL2-AS1 | |
| 11/13 | 2/0 | B | chr12:27464769-27546566 | 12p11.22 | 81.797 | Del | SMCO2 | |
| 15 | 1/0 | LP | chr12:130039668-130299366 | 12q24.33 | 259.699 | Del | ADGRD1, LACAT8, LINC01257 | |
| 1 | 1/0 | LP | chr13:19875814-19957969 | 13q12.11 | 82.156 | Del | CRYL1, MIR4499 | |
| 11 | 1/0 | LB | chr13:61786260-62104565 | 13q21.31 | 318.306 | Del |  | |
| 14 | 0/1 | B | chr14:19323579-19446107 | 14q11.2 | 122.529 | Dup | OR4N2, OR4K2 | |
| 12 | 0/1 | LP | chr14:21321927-21391295 | 14q11.2 | 69.368 | Del |  | |
| 7/8/12/14/15 | 3/2 | LP | chr14:21391295-21667866 | 14q11.2 | 276.571 | Del |  | |
| 4/7/8/10/12/14/15 | 4/3 | LP | chr14:21667866-21803299 | 14q11.2 | 135.433 | Del |  | |
| 4/7/8/10/12/13/14/15 | 5/3 | LP | chr14:21803299-21863320 | 14q11.2 | 60.021 | Del |  | |
| 2/4/7/8/9/10/11/12/13/14/15 | 8/3 | LP | chr14:21863320-21936576 | 14q11.2 | 73.256 | Del |  | |
| 2/4/5/7/8/9/10/11/12/13/14/15 | 9/3 | B | chr14:21936576-21936635 | 14q11.2 | 0.059 | Del |  | |
| 2/5/7/8/9/10/11/12/13/14/15 | 9/2 | LP | chr14:21936635-22034762 | 14q11.2 | 98.127 | Del |  | |
| 10/15 | 2/0 | LP | chr14:22034762-22086438 | 14q11.2 | 51.676 | Del |  | |
| 15 | 1/0 | LP | chr14:22086438-22354007 | 14q11.2 | 267.569 | Del | DAD1, OXA1L, SLC7A7, ABHD4 | |
| 16 | 1/0 | LP | chr14:24417734-26258532 | 14q12 | 1840.798 | Dup | STXBP6, NOVA1 | |
| 7/8 | 2/0 | LP | chr14:99428831-99578034 | 14q32.2 | 149.203 | Del | EML1 | |
| 1/2/3/4/5/6/7/8/9/10/11/12/13/14/15/16 | 13/3 | LB | chr14:105467525-105481523 | 14q32.33 | 13.998 | Dup | KIAA0125 | |
| 1/2/3/4/5/6/7/8/9/10/11/12/13/14/15/16 | 13/3 | B | chr14:105481523-105787449 | 14q32.33 | 305.926 | Dup | ADAM6 | |
| 1/2/3/4/5/6/7/8/9/10/11/12/13/14/15 | 12/3 | B | chr14:105787449-105874352 | 14q32.33 | 86.903 | Dup | LINC00226 | |
| 4/14 | 0/2 | LB | chr14:105874352-105946993 | 14q32.33 | 72.641 | Dup | LINC00221 | |
| 1/2/3/5/6/7/8/9/10/11/13/15 | 13/0 | LB | chr14:105946993-106160234 | 14q32.33 | 213.241 | Dup | LINC00221 | |
| 1/2/3/5/7/8/9/10/11/13/15 | 12/0 | LB | chr14:106160234-106223196 | 14q32.33 | 62.962 | Dup | MIR7641-2 | |
| 4/12 | 0/2 | - | chr15:18741716-19427409 | 15q11.2 | 685.694 | Dup | NBEAP1, CHEK2P2, HERC2P3, GOLGA6L6, GOLGA8CP, LOC646214, CXADRP2, MIR3118-4, MIR3118-2, MIR3118-3, POTEB3, POTEB, POTEB2, NF1P2, MIR5701-3, MIR5701-1, MIR5701-2, LINC01193 | |
| 4 | 0/1 | - | chr15:19427409-19820020 | 15q11.2 | 392.611 | Dup | LOC101927079, LOC727924 | |
| 3/5/6/10/15 | 5/0 | - | chr15:18835660-19819961 | 15q11.2 | 984.301 | Del | NBEAP1, HERC2P3, GOLGA6L6, GOLGA8CP, LOC646214, CXADRP2, MIR3118-4, MIR3118-2, MIR3118-3, POTEB3, POTEB, POTEB2, NF1P2, MIR5701-3, MIR5701-1, MIR5701-2, LINC01193 | |
| 3/5/6/10/15/16 | 6/0 | B | chr15:19819961-19934051 | 15q11.2 | 114.091 | Del | LOC101927079, LOC727924, OR4M2, OR4N4, OR4N3P | |
| 3/5/6/10/15 | 5/0 | B | chr15:19934051-20010618 | 15q11.2 | 76.567 | Del |  | |
| 3/5/10/15 | 4/0 | B | chr15:20010618-20335946 | 15q11.2 | 325.328 | Del | MIR1268A, REREP3, MIR4509-2, MIR4509-1, MIR4509-3, GOLGA8DP, GOLGA6L1 | |
| 15 | 1/0 | P | chr15:29809025-30298155 | 15q13.3 | 489.131 | Del | CHRNA7 | |
| 14 | 0/1 | LP | chr16:1942266-2021433 | 16p13.3 | 79.167 | Dup | NDUFB10, RPS2, TBL3, NOXO1, GFER, SYNGR3, NPW, RPL3L, SNORA10, SNORA64, SNHG9, SNORA78, RNF151, ZNF598 | |
| 12/14 | 0/2 | LP | chr16:2021433-2484806 | 16p13.3 | 463.373 | Dup | SLC9A3R2, NTHL1, TSC2, PKD1, MIR1225, RAB26, TRAF7, CASKIN1, MLST8, PGP, E4F1, ECI1, ABCA3, CCNF, MIR6511B1, MIR6511B2, MIR4516, MIR3180-5, SNHG19, SNORD60, BRICD5, DNASE1L2, RNPS1, LOC106660606, MIR3677, MIR940, MIR4717, ABCA17P, MIR6767, C16orf59, MIR6768, NTN3 | |
| 4/12/14 | 0/3 | LP | chr16:2484806-2747528 | 16p13.3 | 262.722 | Dup | TBC1D24, ATP6V0C, CEMP1, PDPK1, KCTD5, SRRM2, AMDHD2, MIR3178, LOC652276, FLJ42627, ERVK13-1, PRSS27, SRRM2-AS1 | |
| 12/14 | 0/2 | LP | chr16:2747528-3081611 | 16p13.3 | 334.083 | Dup | PRSS33, PRSS21, PRSS22, KREMEN2, PAQR4, PKMYT1, CLDN9, CLDN6, TNFRSF12A, THOC6, MMP25, IL32,TCEB2, PRSS41, ZG16B, PRSS30P, FLYWCH2, FLYWCH1, LINC00514, LOC101929613, HCFC1R1, CCDC64B, LOC100128770, MMP25-AS1, ZSCAN10 | |
| 6 | 1/0 | LP | chr16:31701059-32532079 | 16p11.2 | 831.020 | Del | ZNF267, HERC2P4, TP53TG3D | |
| 6/16 | 2/0 | B | chr16:32532079-33397025 | 16p11.2 | 864.947 | Del | TP53TG3, TP53TG3B, TP53TG3C, SLC6A10P, LOC390705 | |
| 6 | 1/0 | B | chr16:33397025-33511969 | 16p11.2 | 114.944 | Del | ENPP7P13 | |
| 9 | 1/0 | LB | chr16:60416416-61464644 | 16q21 | 1048.228 | Dup | CDH8 | |
| 2/9 | 2/0 | LP | chr16:61464644-64965235 | 16q21 | 3500.591 | Dup | CDH11, CDH5, LOC101927650, LINC00922 | |
| 9 | 1/0 | LP | chr16:64965235-65048937 | 16q21 | 83.702 | Dup | LINC00920,  BEAN1 | |
| 3 | 1/0 | LP | chr17:55063370-55129503 | 17q23.1 | 66.134 | Del | CLTC, PTRH2 | |
| 1 | 1/0 | LP | chr18:70905730-71041077 | 18q22.3 | 135.348 | Dup | ZNF407, ZADH2 | |
| 8 | 1/0 | LP | chr19:47887897-48614523 | 19q13.31 | 726.627 | Del | PSG3, PSG8, PSG1, PSG6, PSG7, PSG11, PSG2, PSG5, PSG4, PSG9, PRG1, CD177, TEX101, LOC100289650, PSG10P, LOC284344 | |
| 7 | 1/0 | P | chr21:24225247-26020469 | 21q21.2-q21.3 | 1795.223 | Del | MIR155, MRPL39, JAM2, ATP5J, LOC101927869, LOC339622, LINC00158, MIR155HG, LINC00515 | |
| 4/12/14 | 0/3 | LP | chr22:21386562-21520273 | 22q11.22 | 133.712 | Dup | MIR650 | |
| 1/8 | 2/0 | LP | chr22:44614141-46395224 | 22q13.31-q13.33 | 1781.083 | Dup | ATXN10, WNT7B, MIRLET7A3, MIRLET7B, PPARA, PKDREJ, TRMU, CELSR1, GRAMD4, CERK, LOC730668, LINC00899, PRR34, PRR34-AS1, MIRLET7BHG, MIR3619, MIR4763, CDPF1, TTC38, GTSE1-AS1, GTSE1, TBC1D22A, TBC1D22A-AS1, LL22NC03-75H12.2 | |
| 1/5/7/8 | 4/0 | P | chr22:46395224-49412774 | 22q13.31-q13.33 | 3017.550 | Dup | ZBED4, ALG12, PIM3, IL17REL, MLC1, MOV10L1, PANX2, SELO, TUBGCP6, MAPK12, PLXNB2, SBF1, NCAPH2, SCO2, TYMP, SYCE3, CPT1B, CHKB, MAPK8IP2, ARSA, LINC00898, LOC284930, MIR3201, FAM19A5, LOC284933, MIR4535, LINC01310, C22orf34, MIR3667, BRD1, CRELD2, MIR6821, TRABD, HDAC10, MAPK11, DENND6B, PPP6R2, ADM2, MIOX, LMF2, ODF3B, KLHDC7B, CHKB-CPT1B, CHKB-AS1 | |
| 5/7 | 2/0 | P | chr22:49412774-49525130 | 22q13.31-q13.33 | 112.356 | Dup | SHANK3, ACR, LOC105373100 | |
| 11 | 1/0 | P | chrX:67331017-68768438 | Xq12-q13.1 | 1437.422 | Dup | OPHN1, STARD8, EFNB1, PJA1, EDA, YIPF6, LINC00269, FAM155B | |
| 4 | 0/1 | LB | chrX:75545456-76114126 | Xq13.3-q21.1 | 568.671 | Dup | MAGEE1, MIR325HG, MIR384 | |
| 8 | 1/0 | LB | chrX:140180757-140617066 | Xq27.2 | 436.31 | Dup | SPANXA1, SPANXA2, SPANXD, SPANXC, SPANXA2-OT1, LOC645188 | |
| 9 | 1/0 | - | chrY:7788596-10622121 | Yp11.2 | 2833.526 | Dup | TSPY1, TTTY18, TTTY19, TTTY11, RBMY1A3P, TTTY20, TSPY4, FAM197Y2P, FAM197Y5P, TSPY8, TSPY3, TSPY10, RBMY3AP, TTTY8, TTTY8B, TTTY7, TTTY7B, TTTY21, TTTY21B, TTTY2,  TTTY2B, TTTY1, TTTY1B, TTTY22, TTTY23, TTTY23B | |
| 2/3/7 | 3/0 | P | chrY:14566592-19266304 | Yq11.221-q11.222 | 4739.859 | Dup | VCY, NLGN4Y, XKRY, VCY1B, NLGN4Y-AS1, FAM41AY2, FAM41AY1, FAM224B, FAM224A, XKRY2, CDY2B, CDY2A, HSFY2, HSFY1, TTTY9A, TTTY9B | |
| M: Male; F: Female; Dup: Duplication; Del: Deletion. P: Pathogenic; LP: Likely Pathogenic; B: Benign; LB: Likely Benign. VOUS: variant of unknown significance; *A total of 9 kinds of CNVs failed to be converted to GRCh37 (hg19), thus, there was no classification for these CNVs using AnnotSV. | | | | | | | | |

| Supplementary Table 2. Functional annotation (biological processes) of the 511 genes from 22 potential pathogenic CNVs (top 20) | | | | |
| --- | --- | --- | --- | --- |
| ID | Description | *P*_value | *P*_adjust | Count |
| GO:0050804 | Modulation of chemical synaptic transmission | 2.6286E-06 | 0.004901335 | 23 |
| GO:0099177 | Regulation of trans-synaptic signaling | 2.73131E-06 | 0.004901335 | 23 |
| GO:2000463 | Positive regulation of excitatory postsynaptic potential | 0.000109605 | 0.131124118 | 5 |
| GO:0050806 | Positive regulation of synaptic transmission | 0.000179191 | 0.141338381 | 11 |
| GO:0035148 | Tube formation | 0.000272195 | 0.141338381 | 10 |
| GO:0035176 | Social behavior | 0.000275667 | 0.141338381 | 6 |
| GO:0051703 | Intraspecies interaction between organisms | 0.000275667 | 0.141338381 | 6 |
| GO:0033617 | Mitochondrial respiratory chain complex IV assembly | 0.00035033 | 0.157166988 | 4 |
| GO:0099565 | Chemical synaptic transmission, postsynaptic | 0.000602573 | 0.187593386 | 8 |
| GO:0007617 | Mating behavior | 0.000614461 | 0.187593386 | 4 |
| GO:0098815 | Modulation of excitatory postsynaptic potential | 0.000694629 | 0.187593386 | 5 |
| GO:0008535 | Respiratory chain complex IV assembly | 0.000727315 | 0.187593386 | 4 |
| GO:0072070 | Loop of Henle development | 0.000777397 | 0.187593386 | 3 |
| GO:0060078 | Regulation of postsynaptic membrane potential | 0.00078313 | 0.187593386 | 9 |
| GO:0031646 | Positive regulation of neurological system process | 0.000784035 | 0.187593386 | 6 |
| GO:0034764 | Positive regulation of transmembrane transport | 0.000927967 | 0.189609157 | 11 |
| GO:1904064 | Positive regulation of cation transmembrane transport | 0.000957162 | 0.189609157 | 9 |
| GO:0046626 | Regulation of insulin receptor signaling pathway | 0.001003782 | 0.189609157 | 6 |
| GO:0099072 | Regulation of postsynaptic membrane neurotransmitter receptor levels | 0.001003782 | 0.189609157 | 6 |
| GO:0051705 | Multi-organism behavior | 0.001086646 | 0.194998559 | 6 |

| Supplementary Table 3. Functional annotation (cellular components) of the 511 genes from 22 potential pathogenic CNVs (top 20) | | | | | |
| --- | --- | --- | --- | --- | --- |
| ID | Description | *P*_value | *P*_adjust | Count | |
| GO:0045095 | Keratin filament | 0.001330593 | 0.298324096 | 7 |  |
| GO:0043209 | Myelin sheath | 0.001555321 | 0.298324096 | 5 |  |
| GO:0005796 | Golgi lumen | 0.002006664 | 0.298324096 | 7 |  |
| GO:0098978 | Glutamatergic synapse | 0.003278314 | 0.365531975 | 14 |  |
| GO:0098984 | Neuron to neuron synapse | 0.008440672 | 0.502301198 | 13 |  |
| GO:0014069 | Postsynaptic density | 0.01138604 | 0.502301198 | 12 |  |
| GO:0032279 | Asymmetric synapse | 0.012448378 | 0.502301198 | 12 |  |
| GO:0005811 | Lipid droplet | 0.013353093 | 0.502301198 | 5 |  |
| GO:0031526 | Brush border membrane | 0.013364144 | 0.502301198 | 4 |  |
| GO:0043220 | Schmidt-Lanterman incisure | 0.014877974 | 0.502301198 | 2 |  |
| GO:0000315 | Organellar large ribosomal subunit | 0.017086785 | 0.502301198 | 4 |  |
| GO:0005762 | Mitochondrial large ribosomal subunit | 0.017086785 | 0.502301198 | 4 |  |
| GO:0000313 | Organellar ribosome | 0.017719284 | 0.502301198 | 5 |  |
| GO:0005761 | Mitochondrial ribosome | 0.017719284 | 0.502301198 | 5 |  |
| GO:0099572 | Postsynaptic specialization | 0.018940947 | 0.502301198 | 12 |  |
| GO:0015934 | Large ribosomal subunit | 0.018994163 | 0.502301198 | 6 |  |
| GO:0032045 | Guanyl-nucleotide exchange factor complex | 0.02062381 | 0.502301198 | 2 |  |
| GO:0098686 | Hippocampal mossy fiber to CA3 synapse | 0.020810963 | 0.502301198 | 3 |  |
| GO:0101003 | Ficolin-1-rich granule membrane | 0.021398482 | 0.502301198 | 4 |  |
| GO:0043218 | Compact myelin | 0.023788839 | 0.530491120 | 2 |  |

| Supplementary Table 4. Functional annotation (molecular function) of the 511 genes from 22 potential pathogenic CNVs (top 20) | | | | |
| --- | --- | --- | --- | --- |
| ID | Description | *P*_value | *P*_adjust | Count |
| GO:1901338 | Catecholamine binding | 0.000358458 | 0.202170058 | 4 |
| GO:0035240 | Dopamine binding | 0.001336421 | 0.376870632 | 3 |
| GO:0000287 | Magnesium ion binding | 0.004439056 | 0.610063599 | 10 |
| GO:0005158 | Insulin receptor binding | 0.006404957 | 0.610063599 | 3 |
| GO:0016298 | Lipase activity | 0.007074428 | 0.610063599 | 7 |
| GO:0015171 | Amino acid transmembrane transporter activity | 0.009612489 | 0.610063599 | 5 |
| GO:0005328 | Neurotransmitter:sodium symporter activity | 0.010271148 | 0.610063599 | 3 |
| GO:0005326 | Neurotransmitter transporter activity | 0.012096665 | 0.610063599 | 4 |
| GO:0008199 | Ferric iron binding | 0.015154792 | 0.610063599 | 2 |
| GO:0016004 | Phospholipase activator activity | 0.017977458 | 0.610063599 | 2 |
| GO:0051011 | Microtubule minus-end binding | 0.017977458 | 0.610063599 | 2 |
| GO:0035255 | Ionotropic glutamate receptor binding | 0.018144977 | 0.610063599 | 3 |
| GO:0031005 | Filamin binding | 0.021003101 | 0.610063599 | 2 |
| GO:0034236 | Protein kinase A catalytic subunit binding | 0.021003101 | 0.610063599 | 2 |
| GO:0043495 | Protein membrane anchor | 0.021003101 | 0.610063599 | 2 |
| GO:0004707 | MAP kinase activity | 0.024223792 | 0.610063599 | 2 |
| GO:0052745 | Inositol phosphate phosphatase activity | 0.024223792 | 0.610063599 | 2 |
| GO:0060229 | Lipase activator activity | 0.024223792 | 0.610063599 | 2 |
| GO:0001227 | Transcriptional repressor activity, RNA polymerase II transcription regulatory region sequence-specific DNA binding | 0.027200246 | 0.610063599 | 9 |
| GO:0005161 | Platelet-derived growth factor receptor binding | 0.027631816 | 0.610063599 | 2 |

| Supplementary Table 5. Pathway enrichment of the 511 genes from 22 potential pathogenic CNVs (top 20) | | | | |
| --- | --- | --- | --- | --- |
| ID | Description | *P*_value | *P*_adjust | Count |
| hsa04728 | Dopaminergic synapse | 5.01576E-05 | 0.009032663 | 10 |
| hsa04140 | Autophagy - animal | 7.34363E-05 | 0.009032663 | 10 |
| hsa04071 | Sphingolipid signaling pathway | 0.000129719 | 0.010636927 | 9 |
| hsa04150 | mTOR signaling pathway | 0.000184442 | 0.011343176 | 10 |
| hsa04912 | GnRH signaling pathway | 0.000763113 | 0.02693539 | 7 |
| hsa05165 | Human papillomavirus infection | 0.000910954 | 0.02693539 | 14 |
| hsa04917 | Prolactin signaling pathway | 0.000925748 | 0.02693539 | 6 |
| hsa05215 | Prostate cancer | 0.000980725 | 0.02693539 | 7 |
| hsa04926 | Relaxin signaling pathway | 0.001163516 | 0.02693539 | 8 |
| hsa04916 | Melanogenesis | 0.001244526 | 0.02693539 | 7 |
| hsa04068 | FoxO signaling pathway | 0.001285203 | 0.02693539 | 8 |
| hsa04714 | Thermogenesis | 0.001313921 | 0.02693539 | 11 |
| hsa04625 | C-type lectin receptor signaling pathway | 0.001476571 | 0.027941258 | 7 |
| hsa04910 | Insulin signaling pathway | 0.001712209 | 0.028826477 | 8 |
| hsa05205 | Proteoglycans in cancer | 0.001757712 | 0.028826477 | 10 |
| hsa04725 | Cholinergic synapse | 0.002262437 | 0.034784965 | 7 |
| hsa04960 | Aldosterone-regulated sodium reabsorption | 0.002919900 | 0.042252677 | 4 |
| hsa05167 | Kaposi sarcoma-associated herpesvirus infection | 0.003246119 | 0.044363629 | 9 |
| hsa05163 | Human cytomegalovirus infection | 0.003589531 | 0.045446465 | 10 |
| hsa05160 | Hepatitis C | 0.003694835 | 0.045446465 | 8 |

| Supplementary Table 6. CNVs-encoded-miRNAs-target genes of ASD (duplication) | |
| --- | --- |
| miRNAs ID | Gene |
| hsa-miR-202-3p | ADA, AGO4, AP3B2, ASAP2, BDNF, CACNA1E, CNTNAP2, CPEB4, DMD, FOXP1, FOXP2, GAD1, GRIA2, KAT6A, KCNJ10, MED13L, MEF2C, NAA15, NFIA, NLGN1, PACS1, PARD3B, PBX1, PCDH19, PRICKLE2, RBFOX1, RIMS1, SHANK2, SNX5, SOAT1, TCF4, TET2, TNRC6A, TNRC6B |
| hsa-miR-202-5p | BDNF, CLASP1, CTNND2, FMR1, KMT2A, MECP2, MED13L, MEF2C, MIB1, MYO1E, NAA15, NFIA, PLXNA4, PTPN11, RERE, ROBO2, TCF4, TNRC6B, USP15, ZMYND11 |
| hsa-miR-210-3p | AGO4, ALDH5A1, CELF4, CNKSR2, CUL3, GGNBP2, MEF2D, MYH11, NHS, NR3C2, OXTR, SEMA5A, SETD2, SMAD4, SYNGAP1, TET2, UPF1 |
| hsa-miR-3178 | CASZ1, EEF1A2, MFRP, SLC12A5 |
| hsa-miR-339-3p | ARX, PCDH19, RAI1, RGS12 |
| hsa-miR-339-5p | ACHE, AFF2, AGO4, ALDH5A1, ARHGEF9, C7orf43, CACNB2, CASK, CASZ1, CDK14, CELF4, DIP2A, DPYSL2, ELAVL3, FOXP1, GABRA5, GRIN2A, KCNMA1, KDM3B, KDM5B, KMT2A, LZTR1, MECP2, MEF2D, MTOR, MYH9, MYO1E, MYO5A, NLGN2, NRXN1, NUAK1, PACS1, PCDHB16, RAB43, RERE, RFX3, SYNGAP1, TRPC6, TUBA1A, UBE3A, UBE3C, VCP |
| hsa-miR-4516 | EFR3A, FN1, GIGYF1, MEF2D, MFRP, SET, SLC6A4, SOX5, TSHZ3 |
| hsa-miR-4717-3p | FOXG1, OPHN1, PLCB1, SCN1A, STAT2, TCF4, WAC |
| hsa-miR-4717-5p | BDNF, CDC42BPB, CNKSR2, LZTR1, SRGAP3, TCF4, ZMYND11 |
| hsa-miR-483-3p | AGO4, ANTXR1, BDNF, CAPRIN1, CASK, CASZ1, CTNNB1, GIGYF1, HIVEP2, KIAA2022, MECP2, MYH9, NSD1, PACS1, PAX5, PAX6, PHF21A, SLC6A8, SMAD4, SON, SRGAP3, TANC2, TBL1XR1, TNRC6B, UBE3C, USP7, ZNF292 |
| hsa-miR-483-5p | ACHE, EBF3, OPHN1, SLC12A5 |
| hsa-miR-675-3p | CPEB4, CSMD3, CUL3, DDX3X, FMR1, MEF2C, NFIA, NRXN1, TBL1XR1 |
| hsa-miR-675-5p | AGAP2, CIB2, EEF1A2, KDM6B, MBOAT7, SLC6A8, UPF1 |
| hsa-miR-940 | ABCC5, AFF2, AGO4, ANTXR1, APH1A, ARHGAP32, ARHGEF9, ASAP2, ASH1L, BAZ2B, BDNF, BRSK2, CASK, CELF4, CEP41, CLASP1, CNTNAP2, CPEB4, CTNND2, DISC1, DLG2, DLG4, DLX3, EP300, GIGYF1, GNAI1, GRIA1, GRID1, HIVEP3, ICA1, IQSEC2, IRF2BPL, JARID2, KCNMA1, KIAA2022, LMX1B, LRP1, LZTR1, MECP2, MEF2C, MFRP, MICAL3, MYH9, MYO15A, MYO9B, NAV2, NLGN2, NR3C2, NRXN2, NSD1, NUAK1, OPHN1, PACS1, PARD3B, PAX5, PBX1, PCDH19, PER2, PLXNA4, PLXNB1, POLR2A, PREX1, PRKD1, PTCHD1, RAB11FIP5, RAB43, RANBP17, ROBO2, SCN9A, SEMA5A, SHOX, SLC12A5, SLC6A3, SLC7A5, SMAD4, SRGAP3, ST8SIA2, STAT2, TANC2, TAOK1, TET2, TRAPPC9, TRIO, UBE3C, ZC3H4 |

| Supplementary Table 7. CNVs-encoded-miRNAs-target genes of ASD (deletion) | |
| --- | --- |
| miRNAs ID | Gene |
| hsa-miR-107 | ABCC5, AFF2, AGO1, AGO4, ANTXR1, ARHGAP32, ASH1L, ATP13A4, BCL11A, BDNF, BRAF, CACNA1E, CAPRIN1, CASK, CASZ1, CDK14, CHD1, CHD2, CUL3, DAPP1, DENR, DLG4, DLGAP1, DMPK, EN2, FASN, FOXP1, GABRG2, GAD1, HTR2A, IRF2BPL, ITPR1, KDM5B, KIAA2022, LRP1, LRP2, LRRC4, MECP2, MEF2D, MIB1, MTOR, MYH9, MYO5A, NAA15, NF1, NFIA, NLGN1, NUAK1, PAK2, PAX5, PLCB1, PLXNA4, RFX3, ROBO2, SCN1A, SCN2A, SCN8A, SEMA5A, SLC6A4, SMAD4, TAOK1, TBL1XR1, TNRC6B, TSC1, VCP, XPO5 |
| hsa-miR-558 | ABCC5, AFF2, AGAP2, AGO4, AHRR, CHD3, CNKSR2, COPS2, CUL3, DPYSL2, DUSP3, GRID1, ITGB3, KAT6A, KDM6B, LAMC3, MECP2, MED13, NR3C2, NSD1, OPHN1, PACS1, PARD3B, PAX5, PHF2, PLXNA4, POMGNT1, PTPN11, RALGAPB, RANBP17, RGS12, SETBP1, SHANK2, SLC12A5, SRGAP3, STXBP1, TGM1, TMLHE, TRAF7, VPS13B |

| Supplementary Table 8. Functional annotation (biological processes) of the 219 target genes of potential pathogenic miRNAs coded within CNVs (top 20) | | | | | |
| --- | --- | --- | --- | --- | --- |
| ID | Description | *P*_value | *P*_adjust | Count | |
| GO:0050808 | Synapse organization | 1.11E-17 | 4.02E-14 | 32 |  |
| GO:0050804 | Modulation of chemical synaptic transmission | 7.63E-17 | 9.87E-14 | 32 |  |
| GO:0099177 | Regulation of trans-synaptic signaling | 8.15E-17 | 9.87E-14 | 32 |  |
| GO:0007611 | Learning or memory | 3.16E-14 | 2.87E-11 | 23 |  |
| GO:0050890 | Cognition | 8.27E-14 | 6.01E-11 | 24 |  |
| GO:0035176 | Social behavior | 6.14E-13 | 3.19E-10 | 12 |  |
| GO:0051703 | Intraspecies interaction between organisms | 6.14E-13 | 3.19E-10 | 12 |  |
| GO:0035249 | Synaptic transmission, glutamatergic | 3.56E-12 | 1.62E-09 | 14 |  |
| GO:0051705 | Multi-organism behavior | 1.53E-11 | 6.19E-09 | 12 |  |
| GO:0010975 | Regulation of neuron projection development | 3.14E-11 | 1.08E-08 | 27 |  |
| GO:0016358 | Dendrite development | 3.28E-11 | 1.08E-08 | 19 |  |
| GO:0042391 | Regulation of membrane potential | 4.74E-11 | 1.37E-08 | 25 |  |
| GO:0006836 | Neurotransmitter transport | 5.20E-11 | 1.37E-08 | 20 |  |
| GO:0097106 | Postsynaptic density organization | 5.30E-11 | 1.37E-08 | 9 |  |
| GO:0099084 | Postsynaptic specialization organization | 9.93E-11 | 2.40E-08 | 9 |  |
| GO:0030900 | Forebrain development | 1.18E-10 | 2.67E-08 | 23 |  |
| GO:0007215 | Glutamate receptor signaling pathway | 1.44E-10 | 3.08E-08 | 13 |  |
| GO:0023061 | Signal release | 1.78E-10 | 3.59E-08 | 25 |  |
| GO:0016569 | Covalent chromatin modification | 3.05E-10 | 5.83E-08 | 25 |  |
| GO:0051966 | Regulation of synaptic transmission, glutamatergic | 4.82E-10 | 8.75E-08 | 11 |  |

| Supplementary Table 9. Functional annotation (cellular components) of the 219 target genes of potential pathogenic miRNAs coded within CNVs (top 20) | | | | |
| --- | --- | --- | --- | --- |
| ID | Description | *P*_value | *P*_adjust | Count |
| GO:0097060 | Synaptic membrane | 6.90E-17 | 2.85E-14 | 31 |
| GO:0099572 | Postsynaptic specialization | 1.18E-15 | 2.43E-13 | 27 |
| GO:0098984 | Neuron to neuron synapse | 1.16E-14 | 1.35E-12 | 26 |
| GO:0045211 | Postsynaptic membrane | 1.52E-14 | 1.35E-12 | 25 |
| GO:0014069 | Postsynaptic density | 1.63E-14 | 1.35E-12 | 25 |
| GO:0032279 | Asymmetric synapse | 2.16E-14 | 1.49E-12 | 25 |
| GO:0098793 | Presynapse | 2.72E-11 | 1.60E-09 | 26 |
| GO:0098978 | Glutamatergic synapse | 2.46E-10 | 1.27E-08 | 21 |
| GO:0033267 | Axon part | 1.27E-09 | 5.81E-08 | 21 |
| GO:0042734 | Presynaptic membrane | 2.55E-09 | 1.05E-07 | 14 |
| GO:0034702 | Ion channel complex | 5.49E-09 | 2.06E-07 | 18 |
| GO:0099699 | Integral component of synaptic membrane | 1.17E-08 | 4.03E-07 | 13 |
| GO:1902495 | Transmembrane transporter complex | 1.72E-08 | 5.48E-07 | 18 |
| GO:0034703 | Cation channel complex | 1.90E-08 | 5.60E-07 | 15 |
| GO:1990351 | Transporter complex | 2.51E-08 | 6.91E-07 | 18 |
| GO:0099240 | Intrinsic component of synaptic membrane | 2.92E-08 | 7.54E-07 | 13 |
| GO:0099634 | Postsynaptic specialization membrane | 1.49E-07 | 3.63E-06 | 10 |
| GO:0099055 | Integral component of postsynaptic membrane | 5.97E-07 | 1.37E-05 | 10 |
| GO:0098936 | Intrinsic component of postsynaptic membrane | 8.80E-07 | 1.91E-05 | 10 |
| GO:0008328 | Ionotropic glutamate receptor complex | 1.24E-06 | 2.56E-05 | 7 |

| Supplementary Table 10. Functional annotation (molecular function) of the 219 target genes of potential pathogenic miRNAs coded within CNVs (top 20) | | | | | |
| --- | --- | --- | --- | --- | --- |
| ID | Description | *P*_value | *P*_adjust | Count | |
| GO:0060589 | Nucleoside-triphosphatase regulator activity | 0.000003690 | 0.001767165 | 16 |  |
| GO:0022839 | Ion gated channel activity | 0.000011400 | 0.001767165 | 15 |  |
| GO:0032452 | Histone demethylase activity | 0.000014600 | 0.001767165 | 5 |  |
| GO:0022836 | Gated channel activity | 0.000015500 | 0.001767165 | 15 |  |
| GO:0030695 | GTPase regulator activity | 0.000016900 | 0.001767165 | 14 |  |
| GO:0005096 | GTPase activator activity | 0.000024000 | 0.001798786 | 13 |  |
| GO:0042393 | Histone binding | 0.000024100 | 0.001798786 | 11 |  |
| GO:0005216 | Ion channel activity | 0.000038800 | 0.002530000 | 16 |  |
| GO:0022838 | Substrate-specific channel activity | 0.000054400 | 0.003124866 | 16 |  |
| GO:0004970 | Ionotropic glutamate receptor activity | 0.000064900 | 0.003124866 | 4 |  |
| GO:0032451 | Demethylase activity | 0.000071800 | 0.003124866 | 5 |  |
| GO:0005248 | Voltage-gated sodium channel activity | 0.000080400 | 0.003124866 | 4 |  |
| GO:0022824 | Transmitter-gated ion channel activity | 0.000083800 | 0.003124866 | 6 |  |
| GO:0022835 | Transmitter-gated channel activity | 0.000083800 | 0.003124866 | 6 |  |
| GO:0015267 | Channel activity | 0.000114023 | 0.003456398 | 16 |  |
| GO:0022803 | Passive transmembrane transporter activity | 0.000116937 | 0.003456398 | 16 |  |
| GO:0005261 | Cation channel activity | 0.000117773 | 0.003456398 | 13 |  |
| GO:0000146 | Microfilament motor activity | 0.000119186 | 0.003456398 | 4 |  |
| GO:0050681 | Androgen receptor binding | 0.000167205 | 0.004593750 | 5 |  |
| GO:0017016 | Ras GTPase binding | 0.000194029 | 0.005064167 | 15 |  |

|  | Supplementary Table 11. Pathway enrichment of the 219 target genes of potential pathogenic miRNAs coded within CNVs (top 20) | | | | |
| --- | --- | --- | --- | --- | --- |
| ID | | Description | *P*_value | *P*_adjust | Count |
| hsa04724 | | Glutamatergic synapse | 0.000064200 | 0.005249750 | 9 |
| hsa04720 | | Long-term potentiation | 0.000073500 | 0.005249750 | 7 |
| hsa05030 | | Cocaine addiction | 0.000100219 | 0.005249750 | 6 |
| hsa04360 | | Axon guidance | 0.000108803 | 0.005249750 | 11 |
| hsa04730 | | Long-term depression | 0.000312236 | 0.011374109 | 6 |
| hsa05033 | | Nicotine addiction | 0.000353599 | 0.011374109 | 5 |
| hsa04713 | | Circadian entrainment | 0.000739626 | 0.020392556 | 7 |
| hsa04728 | | Dopaminergic synapse | 0.000943071 | 0.022751598 | 8 |
| hsa04723 | | Retrograde endocannabinoid signaling | 0.002063498 | 0.043289469 | 8 |
| hsa04919 | | Thyroid hormone signaling pathway | 0.002436947 | 0.043289469 | 7 |
| hsa00310 | | Lysine degradation | 0.002467275 | 0.043289469 | 5 |
| hsa04371 | | Apelin signaling pathway | 0.005324024 | 0.085628051 | 7 |
| hsa04928 | | Parathyroid hormone synthesis, secretion and action | 0.005970482 | 0.088638690 | 6 |
| hsa04726 | | Serotonergic synapse | 0.008801709 | 0.121337851 | 6 |
| hsa04934 | | Cushing syndrome | 0.010246377 | 0.131021893 | 7 |
| hsa04540 | | Gap junction | 0.011674515 | 0.131021893 | 5 |
| hsa04310 | | Wnt signaling pathway | 0.012066037 | 0.131021893 | 7 |
| hsa04727 | | GABAergic synapse | 0.012219658 | 0.131021893 | 5 |
| hsa04022 | | cGMP-PKG signaling pathway | 0.014992069 | 0.152287855 | 7 |
| hsa04068 | | FoxO signaling pathway | 0.016022243 | 0.154614646 | 6 |

| Supplementary Table 12. Comparison of CNVs involved in ASD | | | | | | | | | | | | | | | | | | | | | | | | | | |  |  |  |  |  |
| --- | --- | --- | --- | --- | --- | --- | --- | --- | --- | --- | --- | --- | --- | --- | --- | --- | --- | --- | --- | --- | --- | --- | --- | --- | --- | --- | --- | --- | --- | --- | --- |
| band_type | Qiu  (China) | AlAyadhi  (Saudi Arabia) | Bitar  (Lebanon) | Bremer  (Sweden) | Celestino-Soper  (USA) | Chen  (China) | Costa  (Canada) | Davis  (USA) | Egger  (Austria) | Eriksson  (Sweden) | Fan  (China) | Görker  (Turkey) | Gazzellone (China) | Girirajan  (USA) | Guo  (China) | Kushima  (Japan) | Leppa  (USA) | Mak  (China) | Marshall  (Canada) | Matsunami  (USA) | Monteiro  (Portugal) | Moreno-De-Luca (USA) | Nava  (Asia/  Europe/  South America/  Sub-  Saharan Africa/  Comoros) | Pinto  (European ancestry) | Prasad  (Canada) | Rosenfeld(USA) | | Sakamoto(Japan) | Sebat  (USA) | Sorte  (Norway) | Yep  (Australia) |
| 1p15.1 (Dup) | 0 | 0 | 0 | 0 | 0 | 0 | 0 | 0 | 0 | 1 | 0 | 0 | 0 | 0 | 0 | 0 | 0 | 0 | 0 | 0 | 0 | 0 | 0 | 0 | 0 | 0 | | 0 | 0 | 0 | 0 |
| 1p21.1-p13.2 (Del) | 0 | 0 | 0 | 0 | 0 | 0 | 0 | 0 | 0 | 0 | 0 | 0 | 0 | 0 | 0 | 1 | 0 | 0 | 0 | 0 | 0 | 0 | 0 | 0 | 0 | 0 | | 0 | 0 | 0 | 0 |
| 1p21.3 (Del) | 0 | 0 | 0 | 0 | 0 | 0 | 0 | 0 | 0 | 0 | 0 | 0 | 0 | 0 | 0 | 0 | 0 | 0 | 0 | 0 | 0 | 0 | 0 | 0 | 2 | 0 | | 0 | 0 | 0 | 0 |
| 1p22.1-p21.1 (Del) | 0 | 0 | 0 | 0 | 0 | 0 | 0 | 0 | 0 | 0 | 0 | 0 | 0 | 0 | 1 | 0 | 0 | 0 | 0 | 0 | 0 | 0 | 0 | 0 | 0 | 0 | | 0 | 0 | 0 | 0 |
| 1p31.1 (Del) | 0 | 0 | 0 | 0 | 0 | 0 | 1 | 0 | 0 | 0 | 0 | 0 | 0 | 0 | 0 | 0 | 0 | 0 | 0 | 0 | 0 | 0 | 1 | 1 | 0 | 0 | | 0 | 0 | 0 | 0 |
| 1p31.3 (Dup) | 0 | 0 | 0 | 0 | 0 | 0 | 0 | 0 | 0 | 0 | 0 | 0 | 0 | 0 | 0 | 0 | 0 | 0 | 0 | 0 | 0 | 0 | 0 | 1 | 0 | 0 | | 0 | 0 | 0 | 0 |
| 1p32.2 (Del) | 0 | 0 | 0 | 0 | 0 | 0 | 0 | 0 | 0 | 0 | 0 | 0 | 0 | 0 | 0 | 0 | 0 | 0 | 0 | 0 | 1 | 0 | 0 | 0 | 0 | 0 | | 0 | 0 | 0 | 0 |
| 1p33 (Del) | 0 | 0 | 0 | 0 | 0 | 0 | 0 | 0 | 0 | 0 | 0 | 0 | 0 | 0 | 0 | 0 | 0 | 0 | 0 | 0 | 0 | 0 | 0 | 4 | 0 | 0 | | 0 | 0 | 0 | 0 |
| 1p34.1 (Dup) | 0 | 0 | 0 | 0 | 0 | 0 | 1 | 0 | 0 | 0 | 0 | 0 | 0 | 0 | 0 | 0 | 0 | 0 | 0 | 0 | 0 | 0 | 0 | 0 | 0 | 0 | | 0 | 0 | 0 | 0 |
| 1p36.22 (Del) | 0 | 0 | 0 | 0 | 0 | 0 | 0 | 0 | 0 | 0 | 0 | 0 | 0 | 0 | 0 | 0 | 0 | 0 | 0 | 0 | 0 | 0 | 0 | 0 | 0 | 1 | | 0 | 0 | 0 | 0 |
| 1p36.23 (Del) | 0 | 0 | 0 | 0 | 0 | 0 | 0 | 0 | 0 | 0 | 0 | 0 | 0 | 0 | 0 | 0 | 0 | 0 | 0 | 0 | 0 | 0 | 0 | 0 | 1 | 0 | | 0 | 0 | 0 | 0 |
| 1p36.23 (Dup) | 0 | 0 | 0 | 0 | 0 | 0 | 0 | 0 | 0 | 0 | 0 | 0 | 0 | 0 | 0 | 0 | 0 | 0 | 0 | 0 | 0 | 0 | 0 | 0 | 1 | 0 | | 0 | 0 | 0 | 0 |
| 1p36.31 (Dup) | 1 | 0 | 0 | 0 | 0 | 0 | 0 | 0 | 0 | 0 | 0 | 0 | 0 | 0 | 0 | 0 | 0 | 0 | 0 | 0 | 0 | 0 | 0 | 0 | 0 | 0 | | 0 | 0 | 0 | 0 |
| 1p36.33 (Del) | 0 | 0 | 0 | 0 | 0 | 0 | 0 | 0 | 0 | 0 | 0 | 0 | 0 | 0 | 0 | 0 | 0 | 0 | 0 | 0 | 0 | 0 | 0 | 1 | 0 | 0 | | 0 | 0 | 0 | 0 |
| 1p36.33 (Dup) | 1 | 0 | 0 | 0 | 0 | 0 | 0 | 0 | 0 | 0 | 0 | 0 | 0 | 0 | 0 | 0 | 0 | 0 | 0 | 0 | 0 | 0 | 0 | 0 | 0 | 0 | | 0 | 0 | 0 | 0 |
| 1q21 (Del) | 0 | 0 | 0 | 0 | 0 | 0 | 0 | 0 | 0 | 0 | 0 | 0 | 0 | 0 | 0 | 0 | 0 | 0 | 0 | 0 | 0 | 1 | 0 | 0 | 0 | 0 | | 0 | 0 | 0 | 0 |
| 1q21.1 (Del) | 0 | 0 | 0 | 0 | 0 | 0 | 0 | 0 | 0 | 0 | 1 | 0 | 0 | 4 | 0 | 0 | 1 | 0 | 0 | 0 | 0 | 1 | 0 | 0 | 0 | 4 | | 0 | 0 | 0 | 0 |
| 1q21.1 (Dup) | 0 | 0 | 0 | 0 | 0 | 0 | 1 | 0 | 0 | 0 | 1 | 0 | 0 | 0 | 0 | 0 | 4 | 0 | 0 | 9 | 1 | 1 | 0 | 1 | 0 | 3 | | 0 | 0 | 0 | 0 |
| 1q21.1-q21.2 (Dup) | 0 | 0 | 0 | 0 | 0 | 0 | 0 | 0 | 0 | 0 | 0 | 0 | 0 | 0 | 0 | 0 | 0 | 0 | 0 | 0 | 1 | 0 | 0 | 0 | 0 | 0 | | 0 | 0 | 0 | 0 |
| 1q21.3 (Del) | 0 | 0 | 0 | 0 | 3 | 0 | 0 | 0 | 0 | 0 | 0 | 0 | 0 | 0 | 0 | 0 | 0 | 0 | 0 | 0 | 0 | 0 | 0 | 0 | 0 | 0 | | 0 | 0 | 0 | 0 |
| 1q21.3 (Dup) | 0 | 0 | 0 | 0 | 2 | 0 | 0 | 0 | 0 | 0 | 0 | 0 | 0 | 0 | 0 | 0 | 0 | 0 | 0 | 0 | 0 | 0 | 0 | 0 | 0 | 0 | | 0 | 0 | 0 | 0 |
| 1q23.2 (Dup) | 0 | 0 | 1 | 0 | 0 | 0 | 0 | 0 | 0 | 0 | 0 | 0 | 0 | 0 | 0 | 0 | 0 | 0 | 0 | 0 | 0 | 0 | 0 | 0 | 0 | 0 | | 0 | 0 | 0 | 0 |
| 1q24.2 (Del) | 0 | 0 | 0 | 0 | 0 | 0 | 0 | 1 | 0 | 0 | 0 | 0 | 0 | 0 | 0 | 0 | 0 | 0 | 0 | 0 | 0 | 0 | 0 | 2 | 0 | 0 | | 0 | 0 | 0 | 0 |
| 1q24.3 (Dup) | 0 | 0 | 0 | 0 | 0 | 0 | 0 | 0 | 1 | 0 | 0 | 0 | 0 | 0 | 0 | 0 | 0 | 0 | 0 | 0 | 0 | 0 | 0 | 0 | 0 | 0 | | 0 | 0 | 0 | 0 |
| 1q25.1 (Dup) | 0 | 0 | 0 | 0 | 0 | 0 | 0 | 0 | 0 | 0 | 0 | 0 | 0 | 0 | 0 | 0 | 0 | 0 | 0 | 0 | 0 | 0 | 0 | 1 | 0 | 0 | | 0 | 0 | 0 | 0 |
| 1q25.2 (Del) | 0 | 0 | 0 | 0 | 0 | 0 | 0 | 0 | 0 | 0 | 0 | 0 | 0 | 0 | 0 | 0 | 0 | 0 | 0 | 0 | 0 | 0 | 0 | 0 | 0 | 3 | | 0 | 0 | 0 | 0 |
| 1q25.3-q31.1 (Del) | 0 | 0 | 0 | 1 | 0 | 0 | 0 | 0 | 0 | 0 | 0 | 0 | 0 | 0 | 0 | 0 | 0 | 0 | 0 | 0 | 0 | 0 | 0 | 0 | 0 | 0 | | 0 | 0 | 0 | 0 |
| 1q31.1 (Del) | 0 | 0 | 0 | 0 | 0 | 1 | 0 | 0 | 0 | 0 | 0 | 0 | 0 | 0 | 0 | 0 | 0 | 0 | 0 | 0 | 0 | 0 | 0 | 0 | 0 | 0 | | 0 | 0 | 0 | 0 |
| 1q31.2 (Del) | 0 | 1 | 0 | 0 | 0 | 0 | 0 | 0 | 0 | 0 | 0 | 0 | 0 | 0 | 0 | 0 | 0 | 0 | 0 | 0 | 0 | 0 | 0 | 0 | 0 | 0 | | 0 | 0 | 0 | 0 |
| 1q32.1 (Dup) | 0 | 0 | 0 | 0 | 0 | 0 | 1 | 0 | 1 | 0 | 0 | 0 | 0 | 0 | 0 | 0 | 0 | 0 | 0 | 0 | 0 | 0 | 0 | 0 | 0 | 0 | | 0 | 0 | 0 | 0 |
| 1q32.1-q32.2 (Del) | 0 | 0 | 0 | 0 | 0 | 0 | 0 | 0 | 0 | 0 | 0 | 0 | 0 | 0 | 0 | 1 | 0 | 0 | 0 | 0 | 0 | 0 | 0 | 0 | 0 | 0 | | 0 | 0 | 0 | 0 |
| 1q41 (Del) | 0 | 0 | 0 | 0 | 0 | 0 | 0 | 0 | 0 | 0 | 0 | 0 | 0 | 0 | 0 | 0 | 0 | 0 | 0 | 22 | 0 | 0 | 0 | 0 | 0 | 0 | | 0 | 0 | 0 | 0 |
| 1q41-q42.12 (Del) | 0 | 0 | 0 | 0 | 0 | 0 | 0 | 0 | 0 | 0 | 0 | 0 | 0 | 0 | 0 | 0 | 0 | 0 | 0 | 0 | 0 | 0 | 0 | 0 | 0 | 1 | | 0 | 0 | 0 | 0 |
| 1q42.13 (Dup) | 4 | 0 | 0 | 0 | 0 | 0 | 0 | 0 | 0 | 0 | 0 | 0 | 0 | 0 | 0 | 0 | 0 | 0 | 0 | 0 | 0 | 0 | 0 | 0 | 0 | 0 | | 0 | 0 | 0 | 0 |
| 1q42.2 (Dup) | 0 | 0 | 0 | 0 | 0 | 0 | 0 | 0 | 0 | 0 | 0 | 0 | 0 | 0 | 0 | 0 | 0 | 0 | 0 | 0 | 0 | 0 | 0 | 1 | 0 | 0 | | 0 | 0 | 0 | 0 |
| 1q43 (Del) | 0 | 0 | 0 | 0 | 0 | 0 | 0 | 0 | 0 | 0 | 0 | 0 | 0 | 0 | 0 | 0 | 0 | 0 | 0 | 0 | 0 | 0 | 0 | 0 | 2 | 0 | | 0 | 0 | 0 | 0 |
| 1q44 (Del) | 0 | 0 | 0 | 0 | 0 | 0 | 0 | 0 | 0 | 0 | 0 | 0 | 0 | 0 | 0 | 0 | 0 | 1 | 0 | 0 | 0 | 0 | 0 | 0 | 0 | 0 | | 0 | 0 | 0 | 0 |
| 1q44 (Dup) | 0 | 0 | 0 | 0 | 0 | 0 | 2 | 0 | 0 | 0 | 0 | 0 | 0 | 0 | 0 | 0 | 0 | 0 | 0 | 0 | 0 | 0 | 0 | 0 | 0 | 0 | | 0 | 0 | 0 | 0 |
| 2p11.2 (Dup) | 0 | 0 | 0 | 0 | 0 | 0 | 0 | 0 | 0 | 0 | 0 | 0 | 0 | 0 | 0 | 0 | 0 | 0 | 0 | 0 | 1 | 0 | 0 | 0 | 0 | 0 | | 0 | 0 | 0 | 0 |
| 2p12 (Del) | 0 | 0 | 0 | 0 | 0 | 0 | 0 | 0 | 0 | 0 | 0 | 0 | 0 | 0 | 0 | 0 | 0 | 0 | 0 | 0 | 0 | 0 | 0 | 4 | 0 | 0 | | 0 | 0 | 0 | 0 |
| 2p12 (Dup) | 0 | 0 | 0 | 0 | 0 | 0 | 0 | 0 | 0 | 0 | 0 | 0 | 0 | 0 | 2 | 0 | 0 | 0 | 0 | 0 | 0 | 0 | 0 | 0 | 0 | 0 | | 0 | 0 | 0 | 0 |
| 2p13.1-p12 (Dup) | 0 | 0 | 0 | 0 | 0 | 1 | 0 | 0 | 0 | 0 | 0 | 0 | 0 | 0 | 0 | 0 | 0 | 0 | 0 | 0 | 0 | 0 | 0 | 0 | 0 | 0 | | 0 | 0 | 0 | 0 |
| 2p16.1 (Dup) | 0 | 0 | 0 | 0 | 0 | 1 | 0 | 0 | 0 | 0 | 0 | 0 | 0 | 0 | 0 | 0 | 0 | 0 | 0 | 0 | 0 | 0 | 0 | 0 | 0 | 0 | | 0 | 1 | 0 | 0 |
| 2p16.3 (Del) | 0 | 0 | 0 | 1 | 0 | 0 | 0 | 0 | 0 | 2 | 0 | 2 | 0 | 0 | 0 | 0 | 0 | 0 | 0 | 4 | 0 | 0 | 0 | 5 | 4 | 0 | | 0 | 0 | 0 | 0 |
| 2p16.3 (Dup) | 0 | 0 | 0 | 0 | 0 | 0 | 0 | 0 | 0 | 0 | 0 | 0 | 0 | 0 | 0 | 0 | 0 | 0 | 0 | 0 | 0 | 0 | 0 | 2 | 0 | 0 | | 0 | 0 | 0 | 0 |
| 2p21 (Dup) | 0 | 0 | 0 | 0 | 0 | 0 | 1 | 0 | 0 | 0 | 0 | 0 | 0 | 0 | 0 | 0 | 0 | 0 | 0 | 0 | 0 | 0 | 0 | 0 | 0 | 0 | | 0 | 0 | 0 | 0 |
| 2p22.3 (Dup) | 0 | 0 | 0 | 0 | 0 | 0 | 1 | 0 | 0 | 0 | 0 | 0 | 0 | 0 | 0 | 0 | 0 | 0 | 0 | 0 | 0 | 0 | 0 | 0 | 0 | 0 | | 0 | 0 | 0 | 0 |
| 2p23.1-p22.3 (Del) | 1 | 0 | 0 | 0 | 0 | 0 | 0 | 0 | 0 | 0 | 0 | 0 | 0 | 0 | 0 | 0 | 0 | 0 | 0 | 0 | 0 | 0 | 0 | 0 | 0 | 0 | | 0 | 0 | 0 | 0 |
| 2p23.3 (Dup) | 0 | 0 | 0 | 0 | 0 | 0 | 0 | 0 | 1 | 0 | 0 | 0 | 0 | 0 | 0 | 0 | 0 | 0 | 0 | 0 | 0 | 0 | 0 | 0 | 0 | 0 | | 0 | 0 | 0 | 0 |
| 2p25.1 (Del) | 0 | 0 | 0 | 0 | 0 | 0 | 0 | 0 | 1 | 0 | 0 | 0 | 0 | 0 | 0 | 0 | 0 | 0 | 0 | 0 | 0 | 0 | 0 | 0 | 0 | 0 | | 0 | 0 | 0 | 0 |
| 2p25.1 (Dup) | 0 | 0 | 0 | 0 | 0 | 0 | 0 | 0 | 0 | 0 | 0 | 0 | 0 | 0 | 0 | 0 | 0 | 0 | 0 | 0 | 0 | 0 | 0 | 1 | 0 | 0 | | 0 | 0 | 0 | 0 |
| 2p25.3 (Del) | 0 | 0 | 0 | 0 | 0 | 0 | 0 | 0 | 0 | 0 | 0 | 0 | 0 | 0 | 0 | 0 | 0 | 0 | 0 | 0 | 0 | 0 | 0 | 0 | 0 | 1 | | 0 | 0 | 0 | 0 |
| 2p25.3 (Dup) | 0 | 0 | 0 | 0 | 0 | 0 | 0 | 0 | 0 | 0 | 0 | 0 | 0 | 0 | 0 | 0 | 0 | 0 | 0 | 0 | 1 | 0 | 0 | 0 | 0 | 0 | | 0 | 0 | 0 | 0 |
| 2q12.1 (Del) | 0 | 0 | 0 | 0 | 0 | 0 | 0 | 0 | 0 | 0 | 0 | 0 | 0 | 0 | 0 | 0 | 0 | 0 | 0 | 0 | 0 | 0 | 0 | 1 | 0 | 0 | | 0 | 0 | 0 | 0 |
| 2q12.2-q12.3 (Dup) | 0 | 0 | 0 | 0 | 0 | 0 | 0 | 0 | 0 | 0 | 0 | 0 | 0 | 0 | 1 | 0 | 0 | 0 | 0 | 0 | 0 | 0 | 0 | 0 | 0 | 0 | | 0 | 0 | 0 | 0 |
| 2q13 (Del) | 0 | 0 | 0 | 0 | 1 | 0 | 0 | 0 | 0 | 0 | 0 | 0 | 0 | 0 | 0 | 0 | 0 | 0 | 0 | 0 | 0 | 0 | 0 | 0 | 0 | 3 | | 0 | 0 | 0 | 0 |
| 2q13 (Dup) | 0 | 0 | 0 | 0 | 0 | 0 | 0 | 0 | 1 | 0 | 0 | 0 | 0 | 0 | 0 | 0 | 0 | 0 | 0 | 0 | 0 | 0 | 0 | 0 | 0 | 0 | | 0 | 0 | 0 | 0 |
| 2q14.1 (Del) | 0 | 0 | 0 | 0 | 0 | 0 | 0 | 0 | 0 | 0 | 0 | 0 | 0 | 0 | 0 | 0 | 0 | 0 | 1 | 0 | 0 | 0 | 0 | 0 | 0 | 0 | | 0 | 0 | 0 | 0 |
| 2q14.1 (Dup) | 0 | 0 | 0 | 0 | 0 | 0 | 0 | 0 | 0 | 0 | 0 | 0 | 0 | 0 | 0 | 0 | 0 | 0 | 1 | 0 | 0 | 0 | 0 | 0 | 0 | 0 | | 0 | 0 | 0 | 0 |
| 2q14.3 (Del) | 0 | 0 | 0 | 0 | 0 | 0 | 0 | 0 | 0 | 0 | 0 | 0 | 0 | 0 | 0 | 0 | 0 | 0 | 0 | 0 | 0 | 0 | 0 | 0 | 2 | 0 | | 0 | 0 | 0 | 0 |
| 2q14.3 (Dup) | 0 | 0 | 0 | 0 | 0 | 1 | 0 | 0 | 0 | 0 | 0 | 0 | 0 | 0 | 0 | 0 | 0 | 0 | 0 | 0 | 0 | 0 | 0 | 0 | 0 | 0 | | 0 | 0 | 0 | 0 |
| 2q14.3-q21.1 (Del) | 0 | 0 | 0 | 0 | 0 | 1 | 0 | 0 | 0 | 0 | 0 | 0 | 0 | 0 | 0 | 0 | 0 | 0 | 0 | 0 | 0 | 0 | 0 | 0 | 0 | 0 | | 0 | 0 | 0 | 0 |
| 2q21.1 (Del) | 0 | 0 | 0 | 0 | 0 | 0 | 0 | 0 | 0 | 1 | 0 | 0 | 0 | 0 | 0 | 0 | 0 | 0 | 0 | 0 | 0 | 0 | 0 | 0 | 0 | 0 | | 0 | 0 | 0 | 0 |
| 2q21.2 (Del) | 0 | 0 | 0 | 0 | 0 | 0 | 0 | 0 | 0 | 0 | 0 | 0 | 0 | 0 | 0 | 0 | 0 | 0 | 0 | 0 | 0 | 0 | 0 | 0 | 1 | 0 | | 0 | 0 | 0 | 0 |
| 2q21.2 (Dup) | 0 | 0 | 0 | 0 | 0 | 0 | 0 | 0 | 0 | 0 | 0 | 0 | 0 | 0 | 0 | 0 | 0 | 0 | 0 | 0 | 0 | 0 | 0 | 0 | 1 | 0 | | 0 | 0 | 0 | 0 |
| 2q21.3 (Del) | 0 | 0 | 0 | 0 | 0 | 0 | 0 | 0 | 0 | 0 | 0 | 1 | 0 | 0 | 0 | 0 | 0 | 0 | 0 | 0 | 0 | 0 | 0 | 0 | 0 | 0 | | 0 | 0 | 0 | 0 |
| 2q23.1 (Dup) | 0 | 0 | 0 | 0 | 0 | 0 | 0 | 0 | 0 | 0 | 0 | 0 | 0 | 0 | 0 | 0 | 0 | 0 | 0 | 0 | 0 | 0 | 0 | 1 | 0 | 0 | | 0 | 0 | 0 | 0 |
| 2q23.3 (Del) | 0 | 0 | 0 | 0 | 0 | 0 | 0 | 0 | 0 | 0 | 0 | 0 | 0 | 0 | 0 | 0 | 0 | 0 | 0 | 0 | 0 | 0 | 0 | 0 | 0 | 1 | | 0 | 0 | 0 | 0 |
| 2q24.2 (Del) | 0 | 0 | 0 | 0 | 0 | 0 | 0 | 0 | 0 | 0 | 0 | 0 | 0 | 0 | 0 | 0 | 0 | 0 | 0 | 0 | 0 | 0 | 0 | 0 | 0 | 0 | | 0 | 1 | 0 | 0 |
| 2q31.1 (Del) | 0 | 0 | 0 | 0 | 0 | 0 | 0 | 0 | 0 | 0 | 0 | 0 | 0 | 0 | 0 | 0 | 0 | 0 | 0 | 0 | 0 | 0 | 0 | 1 | 0 | 0 | | 0 | 0 | 0 | 0 |
| 2q31.2 (Del) | 0 | 0 | 0 | 0 | 0 | 0 | 0 | 0 | 0 | 0 | 0 | 0 | 0 | 0 | 0 | 0 | 0 | 0 | 0 | 0 | 0 | 0 | 0 | 1 | 0 | 0 | | 0 | 0 | 0 | 0 |
| 2q32.1 (Del) | 0 | 0 | 0 | 0 | 0 | 0 | 0 | 0 | 0 | 0 | 0 | 0 | 0 | 0 | 0 | 0 | 0 | 0 | 2 | 0 | 0 | 0 | 0 | 0 | 0 | 0 | | 0 | 0 | 0 | 0 |
| 2q32.1-q33.1 (Del) | 0 | 0 | 0 | 0 | 0 | 0 | 1 | 0 | 0 | 0 | 0 | 0 | 0 | 0 | 0 | 0 | 0 | 0 | 0 | 0 | 0 | 0 | 0 | 0 | 0 | 0 | | 0 | 0 | 0 | 0 |
| 2q33.3-q34 (Del) | 0 | 0 | 0 | 0 | 0 | 0 | 0 | 0 | 0 | 0 | 0 | 0 | 0 | 0 | 0 | 0 | 0 | 0 | 0 | 0 | 0 | 0 | 0 | 0 | 0 | 1 | | 0 | 0 | 0 | 0 |
| 2q34 (Del) | 0 | 0 | 0 | 1 | 0 | 0 | 0 | 0 | 0 | 0 | 0 | 0 | 0 | 0 | 0 | 0 | 0 | 0 | 0 | 0 | 0 | 0 | 0 | 0 | 2 | 0 | | 0 | 0 | 0 | 0 |
| 2q34 (Dup) | 0 | 0 | 0 | 0 | 0 | 0 | 0 | 0 | 0 | 0 | 0 | 0 | 0 | 0 | 0 | 0 | 0 | 0 | 0 | 0 | 0 | 0 | 0 | 5 | 0 | 0 | | 0 | 0 | 0 | 0 |
| 2q35 (Dup) | 0 | 0 | 0 | 0 | 0 | 0 | 0 | 0 | 0 | 0 | 0 | 0 | 0 | 0 | 0 | 0 | 0 | 0 | 0 | 0 | 0 | 0 | 0 | 1 | 0 | 0 | | 0 | 0 | 0 | 0 |
| 2q36 (Del) | 0 | 0 | 0 | 0 | 0 | 0 | 0 | 0 | 0 | 0 | 0 | 0 | 0 | 0 | 0 | 0 | 0 | 0 | 0 | 0 | 0 | 0 | 1 | 0 | 0 | 0 | | 0 | 0 | 0 | 0 |
| 2q36.3 (Del) | 0 | 1 | 0 | 0 | 0 | 0 | 0 | 0 | 0 | 0 | 0 | 0 | 0 | 0 | 0 | 0 | 0 | 0 | 0 | 0 | 0 | 0 | 0 | 0 | 0 | 0 | | 0 | 0 | 0 | 0 |
| 2q36.3 (Dup) | 0 | 0 | 0 | 0 | 0 | 0 | 0 | 0 | 0 | 0 | 0 | 0 | 0 | 0 | 1 | 0 | 0 | 0 | 0 | 0 | 0 | 0 | 0 | 0 | 0 | 0 | | 0 | 0 | 0 | 0 |
| 2q37.1 (Del) | 0 | 0 | 0 | 0 | 0 | 0 | 0 | 0 | 0 | 0 | 0 | 0 | 1 | 0 | 0 | 0 | 0 | 0 | 0 | 0 | 0 | 0 | 0 | 0 | 0 | 0 | | 0 | 0 | 0 | 0 |
| 2q37.1 (Dup) | 0 | 0 | 0 | 0 | 0 | 0 | 0 | 0 | 0 | 0 | 0 | 0 | 0 | 0 | 0 | 0 | 0 | 0 | 0 | 0 | 0 | 0 | 0 | 0 | 0 | 2 | | 0 | 0 | 0 | 0 |
| 2q37.2-q37.3 (Del) | 0 | 0 | 0 | 0 | 0 | 0 | 0 | 0 | 0 | 0 | 0 | 0 | 0 | 0 | 0 | 0 | 0 | 0 | 0 | 0 | 0 | 0 | 0 | 0 | 0 | 0 | | 0 | 1 | 0 | 0 |
| 2q37.3 (Del) | 0 | 0 | 0 | 0 | 0 | 1 | 0 | 0 | 0 | 0 | 0 | 0 | 0 | 0 | 0 | 0 | 0 | 0 | 0 | 0 | 0 | 0 | 0 | 0 | 0 | 0 | | 0 | 1 | 0 | 0 |
| 2q37.3 (Dup) | 0 | 0 | 0 | 0 | 0 | 0 | 1 | 0 | 0 | 0 | 0 | 0 | 0 | 0 | 0 | 0 | 0 | 0 | 0 | 0 | 0 | 0 | 0 | 0 | 0 | 0 | | 0 | 0 | 0 | 0 |
| 3p11.1 (Del) | 0 | 0 | 0 | 0 | 0 | 0 | 0 | 0 | 1 | 0 | 0 | 0 | 0 | 0 | 0 | 0 | 0 | 0 | 0 | 0 | 0 | 0 | 0 | 1 | 0 | 0 | | 0 | 0 | 0 | 0 |
| 3p12.3 (Del) | 0 | 0 | 0 | 0 | 0 | 0 | 0 | 0 | 0 | 0 | 0 | 0 | 0 | 0 | 0 | 0 | 0 | 0 | 0 | 0 | 0 | 0 | 0 | 0 | 2 | 0 | | 0 | 0 | 0 | 0 |
| 3p12.3-p12.2 (Dup) | 0 | 0 | 0 | 0 | 0 | 0 | 0 | 0 | 0 | 0 | 0 | 0 | 0 | 0 | 0 | 0 | 0 | 0 | 0 | 0 | 0 | 0 | 0 | 0 | 0 | 1 | | 0 | 0 | 0 | 0 |
| 3p14.1 (Dup) | 0 | 0 | 0 | 0 | 0 | 0 | 0 | 0 | 0 | 0 | 1 | 0 | 0 | 0 | 0 | 0 | 0 | 0 | 0 | 0 | 0 | 0 | 0 | 0 | 0 | 0 | | 0 | 0 | 0 | 0 |
| 3p14.2 (Del) | 0 | 0 | 0 | 0 | 0 | 0 | 0 | 0 | 0 | 0 | 0 | 0 | 0 | 0 | 0 | 0 | 0 | 0 | 0 | 0 | 0 | 0 | 0 | 0 | 0 | 0 | | 0 | 1 | 0 | 0 |
| 3p14.2 (Dup) | 0 | 0 | 0 | 0 | 0 | 0 | 0 | 0 | 0 | 0 | 0 | 0 | 0 | 0 | 0 | 0 | 0 | 0 | 0 | 0 | 0 | 0 | 0 | 0 | 0 | 0 | | 0 | 1 | 1 | 0 |
| 3p21.31 (Dup) | 0 | 0 | 0 | 0 | 0 | 0 | 0 | 0 | 0 | 0 | 0 | 0 | 0 | 0 | 0 | 0 | 0 | 0 | 0 | 0 | 0 | 0 | 0 | 1 | 0 | 0 | | 0 | 0 | 0 | 0 |
| 3p24.3 (Del) | 0 | 0 | 0 | 0 | 0 | 0 | 0 | 0 | 0 | 0 | 0 | 0 | 0 | 0 | 0 | 0 | 0 | 0 | 0 | 0 | 0 | 0 | 0 | 1 | 0 | 0 | | 0 | 0 | 0 | 0 |
| 3p24.3 (Dup) | 0 | 0 | 0 | 0 | 0 | 1 | 0 | 0 | 0 | 0 | 0 | 0 | 0 | 0 | 0 | 0 | 0 | 0 | 0 | 0 | 0 | 0 | 0 | 1 | 0 | 0 | | 0 | 0 | 0 | 0 |
| 3p25.3 (Dup) | 0 | 0 | 0 | 1 | 0 | 0 | 0 | 0 | 0 | 0 | 0 | 0 | 0 | 0 | 0 | 0 | 0 | 0 | 0 | 0 | 0 | 0 | 0 | 0 | 0 | 0 | | 0 | 0 | 0 | 0 |
| 3p26.1 (Del) | 0 | 0 | 0 | 0 | 1 | 0 | 0 | 0 | 0 | 0 | 0 | 0 | 0 | 0 | 0 | 0 | 0 | 0 | 0 | 0 | 0 | 0 | 0 | 0 | 0 | 0 | | 0 | 0 | 0 | 0 |
| 3p26.1 (Dup) | 0 | 0 | 0 | 0 | 0 | 0 | 0 | 1 | 0 | 0 | 0 | 0 | 0 | 0 | 0 | 0 | 0 | 0 | 0 | 0 | 0 | 0 | 0 | 0 | 0 | 0 | | 0 | 0 | 0 | 0 |
| 3p26.2 (Del) | 0 | 0 | 0 | 0 | 0 | 0 | 0 | 0 | 0 | 0 | 0 | 0 | 0 | 0 | 0 | 0 | 0 | 0 | 0 | 0 | 0 | 0 | 0 | 2 | 0 | 0 | | 0 | 0 | 0 | 0 |
| 3p26.2 (Dup) | 0 | 0 | 0 | 0 | 0 | 0 | 0 | 1 | 0 | 0 | 0 | 0 | 0 | 0 | 0 | 0 | 0 | 0 | 0 | 0 | 0 | 0 | 0 | 0 | 0 | 0 | | 0 | 0 | 0 | 0 |
| 3p26.3 (Del) | 0 | 0 | 0 | 0 | 0 | 0 | 2 | 0 | 0 | 0 | 0 | 0 | 0 | 0 | 0 | 0 | 0 | 0 | 0 | 0 | 1 | 0 | 0 | 1 | 0 | 0 | | 0 | 0 | 0 | 0 |
| 3p26.3 (Dup) | 0 | 0 | 1 | 0 | 0 | 0 | 0 | 0 | 0 | 0 | 0 | 0 | 0 | 0 | 1 | 1 | 0 | 0 | 0 | 0 | 0 | 0 | 0 | 2 | 0 | 0 | | 0 | 0 | 0 | 0 |
| 3p26.3-p26.2 (Dup) | 0 | 0 | 0 | 0 | 0 | 0 | 0 | 0 | 0 | 0 | 0 | 0 | 0 | 0 | 1 | 0 | 0 | 0 | 0 | 0 | 0 | 0 | 0 | 0 | 0 | 0 | | 0 | 0 | 0 | 0 |
| 3q11.2 (Dup) | 0 | 0 | 0 | 0 | 0 | 0 | 0 | 0 | 0 | 0 | 0 | 0 | 0 | 0 | 0 | 0 | 0 | 0 | 0 | 0 | 0 | 0 | 0 | 1 | 0 | 0 | | 0 | 0 | 0 | 0 |
| 3q13.31 (Del) | 0 | 0 | 0 | 0 | 0 | 0 | 0 | 0 | 0 | 0 | 0 | 0 | 0 | 0 | 0 | 0 | 0 | 0 | 0 | 0 | 0 | 0 | 0 | 1 | 0 | 0 | | 0 | 0 | 0 | 0 |
| 3q26.1 (Del) | 0 | 0 | 0 | 0 | 0 | 0 | 0 | 0 | 0 | 0 | 0 | 0 | 0 | 0 | 0 | 0 | 0 | 0 | 0 | 0 | 0 | 0 | 0 | 2 | 0 | 0 | | 0 | 0 | 0 | 0 |
| 3q26.31 (Del) | 0 | 0 | 1 | 0 | 0 | 0 | 0 | 0 | 0 | 0 | 0 | 0 | 0 | 0 | 0 | 0 | 0 | 0 | 0 | 0 | 0 | 0 | 0 | 0 | 0 | 0 | | 0 | 0 | 0 | 0 |
| 3q26.31 (Dup) | 0 | 0 | 0 | 0 | 0 | 0 | 0 | 0 | 0 | 0 | 0 | 0 | 0 | 0 | 0 | 0 | 0 | 0 | 0 | 1 | 0 | 0 | 0 | 1 | 0 | 0 | | 0 | 0 | 0 | 0 |
| 3q27.2-q29 (Del) | 0 | 0 | 0 | 1 | 0 | 0 | 0 | 0 | 0 | 0 | 0 | 0 | 0 | 0 | 0 | 0 | 0 | 0 | 0 | 0 | 0 | 0 | 0 | 0 | 0 | 0 | | 0 | 0 | 0 | 0 |
| 3q29 (Del) | 0 | 0 | 0 | 0 | 0 | 0 | 0 | 0 | 0 | 0 | 0 | 0 | 0 | 1 | 0 | 0 | 0 | 0 | 0 | 0 | 0 | 1 | 1 | 0 | 0 | 2 | | 0 | 0 | 0 | 0 |
| 3q29 (Dup) | 0 | 0 | 0 | 0 | 0 | 0 | 0 | 0 | 0 | 0 | 0 | 0 | 0 | 3 | 0 | 1 | 0 | 0 | 0 | 0 | 0 | 0 | 0 | 0 | 0 | 0 | | 0 | 0 | 0 | 0 |
| 4p13 (Dup) | 0 | 0 | 0 | 0 | 0 | 1 | 0 | 0 | 0 | 0 | 0 | 0 | 0 | 0 | 0 | 0 | 0 | 0 | 0 | 0 | 0 | 0 | 0 | 0 | 0 | 0 | | 0 | 0 | 0 | 0 |
| 4p15.31 (Del) | 0 | 0 | 0 | 0 | 0 | 0 | 0 | 0 | 0 | 0 | 0 | 0 | 0 | 0 | 0 | 0 | 0 | 0 | 0 | 0 | 0 | 0 | 0 | 1 | 0 | 0 | | 0 | 0 | 0 | 0 |
| 4p15.31 (Dup) | 0 | 0 | 0 | 0 | 0 | 0 | 0 | 0 | 0 | 0 | 0 | 1 | 0 | 0 | 0 | 0 | 0 | 0 | 0 | 0 | 0 | 0 | 0 | 0 | 0 | 0 | | 0 | 0 | 0 | 0 |
| 4p16.1 (Dup) | 0 | 2 | 0 | 0 | 0 | 0 | 1 | 0 | 0 | 0 | 0 | 0 | 0 | 0 | 0 | 0 | 0 | 0 | 0 | 0 | 0 | 0 | 0 | 0 | 0 | 0 | | 0 | 0 | 0 | 0 |
| 4p16.3-p16.1 (Del) | 0 | 0 | 0 | 0 | 0 | 0 | 1 | 0 | 0 | 0 | 0 | 0 | 0 | 0 | 0 | 0 | 0 | 0 | 0 | 0 | 0 | 0 | 0 | 0 | 0 | 0 | | 0 | 0 | 0 | 0 |
| 4q12-q13.1 (Dup) | 0 | 0 | 0 | 0 | 0 | 1 | 0 | 0 | 0 | 0 | 0 | 0 | 0 | 0 | 0 | 0 | 0 | 0 | 0 | 0 | 0 | 0 | 0 | 0 | 0 | 0 | | 0 | 0 | 0 | 0 |
| 4q13.1 (Del) | 0 | 0 | 0 | 0 | 0 | 0 | 0 | 0 | 0 | 0 | 0 | 0 | 0 | 0 | 0 | 0 | 0 | 0 | 0 | 0 | 0 | 0 | 0 | 1 | 0 | 0 | | 0 | 0 | 0 | 0 |
| 4q13.1 (Dup) | 0 | 0 | 0 | 0 | 0 | 0 | 0 | 0 | 0 | 0 | 0 | 0 | 0 | 1 | 0 | 0 | 0 | 0 | 0 | 0 | 0 | 0 | 0 | 0 | 0 | 0 | | 0 | 0 | 0 | 0 |
| 4q13.2 (Del) | 0 | 0 | 0 | 0 | 2 | 0 | 0 | 0 | 0 | 0 | 0 | 0 | 0 | 0 | 0 | 0 | 0 | 0 | 0 | 0 | 0 | 0 | 0 | 0 | 0 | 0 | | 0 | 0 | 0 | 0 |
| 4q13.2 (Dup) | 0 | 0 | 0 | 0 | 1 | 0 | 0 | 0 | 0 | 0 | 0 | 0 | 0 | 0 | 0 | 0 | 0 | 0 | 0 | 0 | 0 | 0 | 0 | 0 | 0 | 0 | | 12 | 0 | 0 | 0 |
| 4q22.2 (Del) | 0 | 0 | 0 | 0 | 0 | 0 | 0 | 0 | 0 | 1 | 0 | 0 | 1 | 0 | 0 | 0 | 0 | 0 | 0 | 0 | 0 | 0 | 0 | 0 | 0 | 0 | | 0 | 0 | 0 | 0 |
| 4q23 (Dup) | 0 | 0 | 0 | 0 | 0 | 0 | 0 | 0 | 0 | 0 | 0 | 0 | 0 | 0 | 0 | 0 | 0 | 0 | 0 | 0 | 0 | 0 | 0 | 0 | 2 | 0 | | 0 | 0 | 0 | 0 |
| 4q26 (Del) | 0 | 0 | 1 | 0 | 0 | 0 | 0 | 0 | 0 | 0 | 0 | 0 | 0 | 0 | 0 | 0 | 0 | 0 | 0 | 0 | 0 | 0 | 0 | 0 | 0 | 0 | | 0 | 0 | 0 | 0 |
| 4q28.1 (Dup) | 0 | 0 | 0 | 0 | 0 | 0 | 1 | 0 | 0 | 0 | 0 | 0 | 1 | 0 | 0 | 0 | 0 | 0 | 0 | 0 | 0 | 0 | 0 | 0 | 0 | 0 | | 0 | 0 | 0 | 0 |
| 4q28.3 (Del) | 0 | 0 | 0 | 0 | 0 | 0 | 0 | 0 | 0 | 0 | 0 | 0 | 0 | 0 | 0 | 0 | 0 | 0 | 0 | 0 | 0 | 0 | 0 | 0 | 0 | 4 | | 0 | 0 | 0 | 0 |
| 4q31.1 (Del) | 0 | 0 | 0 | 0 | 0 | 0 | 0 | 0 | 0 | 0 | 0 | 0 | 0 | 0 | 0 | 0 | 0 | 0 | 0 | 0 | 0 | 0 | 0 | 1 | 0 | 0 | | 0 | 0 | 0 | 0 |
| 4q31.21-q33 (Dup) | 0 | 0 | 0 | 0 | 0 | 0 | 0 | 0 | 0 | 0 | 1 | 0 | 0 | 0 | 0 | 0 | 0 | 0 | 0 | 0 | 0 | 0 | 0 | 0 | 0 | 0 | | 0 | 0 | 0 | 0 |
| 4q31.22 (Del) | 0 | 0 | 0 | 0 | 0 | 1 | 0 | 0 | 0 | 0 | 0 | 0 | 0 | 0 | 0 | 0 | 0 | 0 | 0 | 0 | 0 | 0 | 0 | 0 | 0 | 0 | | 0 | 0 | 0 | 0 |
| 4q31.23 (Del) | 0 | 0 | 0 | 0 | 0 | 0 | 1 | 0 | 0 | 0 | 0 | 0 | 0 | 0 | 0 | 0 | 0 | 0 | 0 | 0 | 0 | 0 | 0 | 0 | 0 | 0 | | 0 | 0 | 0 | 0 |
| 4q32.3 (Del) | 0 | 0 | 0 | 0 | 0 | 0 | 0 | 0 | 0 | 0 | 0 | 0 | 0 | 1 | 0 | 0 | 0 | 0 | 0 | 0 | 0 | 0 | 0 | 0 | 0 | 0 | | 0 | 0 | 0 | 0 |
| 4q33 (Del) | 0 | 0 | 0 | 0 | 0 | 0 | 0 | 0 | 0 | 0 | 0 | 0 | 0 | 0 | 0 | 0 | 0 | 0 | 0 | 0 | 0 | 0 | 0 | 0 | 0 | 2 | | 0 | 0 | 0 | 0 |
| 4q34.3 (Dup) | 0 | 0 | 0 | 0 | 0 | 0 | 0 | 1 | 0 | 0 | 0 | 0 | 0 | 0 | 0 | 0 | 0 | 0 | 0 | 0 | 0 | 0 | 0 | 0 | 0 | 0 | | 0 | 0 | 0 | 0 |
| 4q35 (Del) | 0 | 0 | 0 | 0 | 0 | 0 | 1 | 0 | 0 | 0 | 0 | 0 | 0 | 0 | 0 | 0 | 0 | 0 | 0 | 0 | 0 | 0 | 0 | 0 | 0 | 0 | | 0 | 0 | 0 | 0 |
| 4q35.1-q35.2 (Dup) | 0 | 0 | 0 | 0 | 0 | 0 | 0 | 0 | 0 | 0 | 0 | 0 | 0 | 0 | 0 | 1 | 0 | 0 | 0 | 0 | 0 | 0 | 0 | 0 | 0 | 0 | | 0 | 0 | 0 | 0 |
| 4q35.2 (Del) | 0 | 0 | 0 | 0 | 0 | 0 | 0 | 0 | 0 | 0 | 0 | 0 | 0 | 0 | 1 | 0 | 0 | 0 | 0 | 2 | 0 | 0 | 0 | 2 | 0 | 0 | | 0 | 0 | 0 | 0 |
| 4q35.2 (Dup) | 0 | 2 | 0 | 0 | 0 | 0 | 0 | 0 | 0 | 0 | 0 | 0 | 0 | 0 | 0 | 0 | 0 | 0 | 0 | 0 | 0 | 0 | 0 | 0 | 0 | 5 | | 0 | 0 | 0 | 0 |
| 5p12 (Del) | 0 | 0 | 0 | 0 | 0 | 0 | 0 | 0 | 0 | 0 | 0 | 0 | 0 | 0 | 0 | 0 | 0 | 0 | 0 | 0 | 0 | 0 | 0 | 1 | 0 | 0 | | 0 | 0 | 0 | 0 |
| 5p12-p11 (Dup) | 0 | 0 | 0 | 0 | 0 | 1 | 0 | 0 | 0 | 0 | 0 | 0 | 0 | 0 | 0 | 0 | 0 | 0 | 0 | 0 | 0 | 0 | 0 | 0 | 0 | 0 | | 0 | 0 | 0 | 0 |
| 5p13.2 (Del) | 0 | 0 | 0 | 0 | 0 | 0 | 1 | 0 | 0 | 0 | 0 | 0 | 0 | 0 | 0 | 0 | 0 | 0 | 0 | 0 | 0 | 0 | 0 | 0 | 0 | 0 | | 0 | 0 | 0 | 0 |
| 5p14.2 (Del) | 0 | 0 | 0 | 0 | 0 | 0 | 0 | 0 | 0 | 0 | 0 | 0 | 0 | 0 | 0 | 0 | 0 | 0 | 0 | 0 | 0 | 0 | 0 | 1 | 0 | 0 | | 0 | 0 | 0 | 0 |
| 5p14.2-p14.1 (Del) | 0 | 0 | 0 | 0 | 0 | 0 | 0 | 0 | 0 | 0 | 0 | 0 | 0 | 0 | 1 | 0 | 0 | 0 | 0 | 0 | 0 | 0 | 0 | 0 | 0 | 0 | | 0 | 0 | 0 | 0 |
| 5p14.3 (Del) | 0 | 0 | 0 | 0 | 0 | 0 | 0 | 0 | 0 | 0 | 0 | 0 | 0 | 0 | 0 | 0 | 0 | 0 | 0 | 0 | 0 | 0 | 0 | 1 | 0 | 0 | | 0 | 0 | 0 | 0 |
| 5p15 (Dup) | 0 | 0 | 0 | 0 | 0 | 0 | 0 | 0 | 0 | 1 | 0 | 0 | 0 | 0 | 0 | 0 | 0 | 0 | 0 | 0 | 0 | 0 | 0 | 0 | 0 | 0 | | 0 | 0 | 0 | 0 |
| 5p15.1-p14.3 (Dup) | 0 | 0 | 0 | 0 | 0 | 0 | 0 | 0 | 0 | 1 | 0 | 0 | 0 | 0 | 0 | 0 | 0 | 0 | 0 | 0 | 0 | 0 | 0 | 0 | 0 | 0 | | 0 | 0 | 0 | 0 |
| 5p15.2 (Del) | 0 | 0 | 0 | 0 | 0 | 0 | 0 | 0 | 0 | 0 | 0 | 0 | 0 | 0 | 0 | 0 | 0 | 0 | 0 | 0 | 1 | 0 | 0 | 0 | 0 | 0 | | 0 | 0 | 0 | 0 |
| 5p15.33 (Dup) | 1 | 0 | 0 | 0 | 0 | 1 | 0 | 0 | 0 | 0 | 0 | 0 | 0 | 0 | 0 | 0 | 0 | 0 | 0 | 0 | 0 | 0 | 0 | 0 | 0 | 0 | | 0 | 0 | 0 | 0 |
| 5p15.33-p15.2 (Del) | 0 | 0 | 0 | 0 | 0 | 0 | 0 | 0 | 0 | 0 | 0 | 0 | 0 | 0 | 1 | 0 | 0 | 0 | 0 | 0 | 0 | 0 | 0 | 0 | 0 | 0 | | 0 | 0 | 0 | 0 |
| 5p15.33-p15.2 (Dup) | 1 | 0 | 0 | 0 | 0 | 0 | 0 | 0 | 0 | 0 | 0 | 0 | 0 | 0 | 0 | 0 | 0 | 0 | 0 | 0 | 0 | 0 | 0 | 0 | 0 | 0 | | 0 | 0 | 0 | 0 |
| 5q11.2 (Dup) | 0 | 0 | 0 | 0 | 0 | 0 | 0 | 0 | 0 | 0 | 0 | 0 | 0 | 0 | 0 | 0 | 0 | 0 | 0 | 0 | 0 | 0 | 0 | 1 | 0 | 0 | | 0 | 0 | 0 | 0 |
| 5q13.2 (Del) | 0 | 0 | 0 | 0 | 3 | 0 | 0 | 0 | 0 | 0 | 0 | 0 | 0 | 0 | 0 | 0 | 0 | 0 | 0 | 0 | 0 | 0 | 0 | 0 | 0 | 0 | | 0 | 0 | 0 | 0 |
| 5q13.2 (Dup) | 0 | 0 | 0 | 0 | 0 | 1 | 0 | 0 | 0 | 0 | 0 | 0 | 0 | 0 | 1 | 0 | 0 | 0 | 0 | 0 | 0 | 0 | 0 | 0 | 0 | 0 | | 0 | 0 | 0 | 0 |
| 5q21.3 (Dup) | 0 | 0 | 1 | 0 | 0 | 0 | 0 | 0 | 0 | 0 | 0 | 0 | 0 | 0 | 0 | 0 | 0 | 0 | 0 | 0 | 0 | 0 | 0 | 0 | 0 | 0 | | 0 | 0 | 0 | 0 |
| 5q21.3-q22.1 (Dup) | 0 | 0 | 0 | 0 | 0 | 0 | 1 | 0 | 0 | 0 | 0 | 0 | 0 | 0 | 0 | 0 | 0 | 0 | 0 | 0 | 0 | 0 | 0 | 0 | 0 | 0 | | 0 | 0 | 0 | 0 |
| 5q22.1 (Dup) | 0 | 0 | 0 | 0 | 0 | 0 | 1 | 0 | 0 | 0 | 0 | 0 | 0 | 0 | 0 | 0 | 0 | 0 | 0 | 0 | 0 | 0 | 0 | 0 | 0 | 0 | | 0 | 0 | 0 | 0 |
| 5q22.3-q23.1 (Dup) | 0 | 0 | 0 | 0 | 0 | 0 | 0 | 0 | 0 | 0 | 0 | 0 | 0 | 0 | 0 | 1 | 0 | 0 | 0 | 0 | 0 | 0 | 0 | 0 | 0 | 0 | | 0 | 0 | 0 | 0 |
| 5q23.1 (Dup) | 0 | 0 | 0 | 0 | 0 | 0 | 0 | 0 | 0 | 0 | 0 | 0 | 0 | 0 | 0 | 0 | 0 | 0 | 0 | 0 | 0 | 0 | 0 | 1 | 0 | 0 | | 0 | 0 | 0 | 0 |
| 5q32 (Del) | 0 | 0 | 0 | 0 | 0 | 1 | 0 | 0 | 0 | 0 | 0 | 0 | 0 | 0 | 0 | 0 | 0 | 0 | 0 | 0 | 0 | 0 | 0 | 0 | 0 | 0 | | 0 | 0 | 0 | 0 |
| 5q33.1 (Del) | 0 | 0 | 1 | 0 | 0 | 0 | 0 | 0 | 0 | 0 | 0 | 0 | 0 | 0 | 0 | 0 | 0 | 0 | 0 | 0 | 0 | 0 | 0 | 0 | 0 | 0 | | 0 | 0 | 0 | 0 |
| 5q35 (Del) | 0 | 0 | 0 | 0 | 0 | 0 | 0 | 0 | 0 | 0 | 0 | 0 | 0 | 0 | 0 | 0 | 1 | 0 | 0 | 0 | 0 | 1 | 0 | 0 | 0 | 0 | | 0 | 0 | 0 | 0 |
| 5q35.3 (Del) | 0 | 0 | 0 | 0 | 1 | 0 | 0 | 0 | 0 | 0 | 0 | 0 | 0 | 0 | 0 | 0 | 0 | 0 | 0 | 0 | 0 | 0 | 0 | 0 | 0 | 1 | | 0 | 0 | 0 | 0 |
| 5q35.3 (Dup) | 0 | 0 | 0 | 0 | 0 | 0 | 0 | 0 | 0 | 0 | 0 | 0 | 0 | 0 | 1 | 0 | 0 | 0 | 0 | 0 | 0 | 0 | 0 | 0 | 0 | 0 | | 0 | 0 | 0 | 0 |
| 6p21.31 (Dup) | 0 | 0 | 0 | 0 | 0 | 0 | 0 | 0 | 0 | 0 | 0 | 0 | 0 | 0 | 0 | 0 | 0 | 0 | 0 | 0 | 0 | 0 | 0 | 1 | 0 | 0 | | 0 | 0 | 0 | 0 |
| 6p21.32 (Del) | 0 | 1 | 0 | 0 | 0 | 0 | 0 | 0 | 0 | 0 | 0 | 0 | 0 | 0 | 0 | 0 | 0 | 0 | 0 | 0 | 0 | 0 | 0 | 1 | 0 | 0 | | 0 | 0 | 0 | 0 |
| 6p22.1 (Del) | 0 | 2 | 0 | 0 | 0 | 0 | 0 | 0 | 0 | 0 | 0 | 0 | 0 | 0 | 0 | 0 | 0 | 0 | 0 | 0 | 0 | 0 | 0 | 0 | 0 | 1 | | 0 | 0 | 0 | 0 |
| 6p22.1 (Dup) | 0 | 0 | 0 | 0 | 0 | 0 | 0 | 0 | 0 | 0 | 0 | 0 | 0 | 0 | 0 | 0 | 0 | 0 | 0 | 0 | 0 | 0 | 0 | 1 | 0 | 0 | | 0 | 0 | 0 | 0 |
| 6p23 (Del) | 0 | 0 | 0 | 0 | 0 | 0 | 0 | 0 | 0 | 0 | 0 | 0 | 0 | 0 | 0 | 0 | 0 | 0 | 0 | 0 | 0 | 0 | 0 | 0 | 0 | 0 | | 0 | 1 | 0 | 0 |
| 6p24.3 (Del) | 0 | 0 | 0 | 0 | 0 | 0 | 0 | 0 | 0 | 0 | 0 | 0 | 0 | 0 | 0 | 0 | 0 | 0 | 0 | 1 | 0 | 0 | 0 | 0 | 0 | 0 | | 0 | 0 | 0 | 0 |
| 6p25 (Del) | 0 | 0 | 0 | 0 | 0 | 0 | 0 | 0 | 1 | 0 | 0 | 0 | 0 | 0 | 0 | 0 | 0 | 0 | 0 | 0 | 0 | 0 | 0 | 0 | 0 | 0 | | 0 | 0 | 0 | 0 |
| 6p25.3 (Dup) | 0 | 0 | 0 | 0 | 0 | 0 | 0 | 0 | 0 | 0 | 0 | 0 | 0 | 0 | 0 | 0 | 0 | 0 | 0 | 0 | 0 | 0 | 0 | 1 | 0 | 0 | | 0 | 0 | 0 | 0 |
| 6q11.1 (Dup) | 0 | 0 | 0 | 0 | 0 | 0 | 0 | 0 | 0 | 0 | 0 | 0 | 0 | 0 | 0 | 0 | 0 | 0 | 0 | 2 | 0 | 0 | 0 | 0 | 0 | 0 | | 0 | 0 | 0 | 0 |
| 6q15 (Dup) | 0 | 0 | 0 | 0 | 0 | 0 | 1 | 0 | 1 | 0 | 0 | 0 | 0 | 0 | 0 | 0 | 0 | 0 | 0 | 0 | 0 | 0 | 0 | 0 | 0 | 0 | | 0 | 0 | 0 | 0 |
| 6q21 (Dup) | 0 | 0 | 0 | 0 | 0 | 0 | 1 | 0 | 0 | 0 | 0 | 0 | 0 | 0 | 0 | 0 | 0 | 0 | 0 | 0 | 0 | 0 | 0 | 1 | 0 | 0 | | 0 | 0 | 0 | 0 |
| 6q22.31 (Dup) | 0 | 0 | 0 | 0 | 0 | 0 | 0 | 0 | 0 | 0 | 0 | 0 | 0 | 1 | 0 | 0 | 0 | 0 | 2 | 0 | 0 | 0 | 0 | 0 | 0 | 0 | | 0 | 0 | 0 | 0 |
| 6q24.3 (Del) | 0 | 0 | 0 | 0 | 0 | 0 | 0 | 1 | 0 | 0 | 0 | 0 | 0 | 0 | 0 | 0 | 0 | 0 | 0 | 1 | 0 | 0 | 0 | 3 | 0 | 0 | | 0 | 0 | 0 | 0 |
| 6q24.3-q27 (Dup) | 0 | 0 | 0 | 0 | 0 | 0 | 0 | 0 | 0 | 0 | 0 | 0 | 0 | 0 | 1 | 0 | 0 | 0 | 0 | 0 | 0 | 0 | 0 | 0 | 0 | 0 | | 0 | 0 | 0 | 0 |
| 6q25.3 (Del) | 0 | 0 | 0 | 0 | 0 | 0 | 0 | 0 | 0 | 0 | 0 | 0 | 0 | 0 | 0 | 0 | 0 | 0 | 0 | 0 | 0 | 0 | 0 | 1 | 0 | 0 | | 0 | 0 | 0 | 0 |
| 6q25.3 (Dup) | 0 | 0 | 0 | 0 | 0 | 0 | 0 | 0 | 0 | 0 | 0 | 0 | 0 | 0 | 0 | 0 | 0 | 0 | 0 | 0 | 0 | 0 | 0 | 1 | 0 | 0 | | 0 | 0 | 0 | 0 |
| 6q26 (Del) | 0 | 0 | 1 | 2 | 0 | 0 | 0 | 0 | 0 | 0 | 0 | 0 | 0 | 0 | 0 | 0 | 0 | 0 | 0 | 0 | 0 | 0 | 0 | 0 | 0 | 0 | | 0 | 0 | 0 | 0 |
| 6q26 (Dup) | 0 | 0 | 0 | 0 | 0 | 0 | 0 | 0 | 0 | 0 | 0 | 0 | 0 | 0 | 0 | 0 | 0 | 1 | 0 | 0 | 0 | 0 | 0 | 0 | 0 | 0 | | 0 | 0 | 0 | 0 |
| 6q27 (Del) | 0 | 0 | 0 | 0 | 0 | 0 | 0 | 0 | 0 | 0 | 0 | 0 | 0 | 0 | 0 | 0 | 0 | 0 | 0 | 0 | 0 | 0 | 0 | 0 | 0 | 4 | | 0 | 0 | 0 | 0 |
| 7p12.1-p11.2 (Dup) | 0 | 0 | 0 | 0 | 0 | 0 | 0 | 0 | 0 | 0 | 0 | 0 | 0 | 0 | 0 | 1 | 0 | 0 | 0 | 0 | 0 | 0 | 0 | 0 | 0 | 0 | | 0 | 0 | 0 | 0 |
| 7p13 (Del) | 0 | 0 | 1 | 0 | 0 | 0 | 0 | 0 | 0 | 0 | 0 | 0 | 0 | 0 | 0 | 0 | 0 | 0 | 0 | 0 | 0 | 0 | 0 | 0 | 0 | 0 | | 0 | 0 | 0 | 0 |
| 7p14.1 (Dup) | 0 | 0 | 0 | 0 | 0 | 1 | 0 | 0 | 0 | 0 | 0 | 0 | 0 | 0 | 0 | 0 | 0 | 0 | 0 | 0 | 1 | 0 | 0 | 0 | 0 | 0 | | 0 | 0 | 0 | 0 |
| 7p14.3 (Dup) | 0 | 0 | 1 | 0 | 0 | 0 | 0 | 0 | 0 | 0 | 0 | 0 | 0 | 0 | 0 | 0 | 0 | 0 | 0 | 0 | 0 | 0 | 0 | 0 | 0 | 0 | | 0 | 0 | 0 | 0 |
| 7p15.1 (Dup) | 0 | 0 | 0 | 0 | 0 | 0 | 0 | 0 | 0 | 0 | 0 | 0 | 0 | 0 | 0 | 0 | 0 | 0 | 0 | 0 | 0 | 0 | 0 | 1 | 0 | 0 | | 0 | 0 | 0 | 0 |
| 7p21.1 (Del) | 0 | 0 | 0 | 1 | 0 | 0 | 0 | 0 | 0 | 0 | 0 | 0 | 0 | 0 | 0 | 0 | 0 | 0 | 0 | 0 | 0 | 0 | 0 | 0 | 0 | 1 | | 0 | 1 | 0 | 0 |
| 7p21.1 (Dup) | 0 | 0 | 0 | 0 | 0 | 0 | 0 | 0 | 0 | 0 | 0 | 0 | 0 | 0 | 0 | 0 | 0 | 0 | 0 | 0 | 0 | 0 | 0 | 1 | 0 | 0 | | 0 | 0 | 0 | 0 |
| 7p22.1 (Dup) | 0 | 0 | 0 | 1 | 0 | 0 | 0 | 0 | 0 | 0 | 0 | 0 | 0 | 0 | 0 | 0 | 0 | 0 | 0 | 1 | 0 | 0 | 0 | 0 | 0 | 0 | | 0 | 0 | 0 | 0 |
| 7p22.3 (Dup) | 2 | 0 | 0 | 0 | 0 | 2 | 0 | 0 | 0 | 0 | 0 | 0 | 0 | 0 | 0 | 0 | 0 | 0 | 0 | 0 | 0 | 0 | 0 | 0 | 0 | 2 | | 0 | 0 | 0 | 0 |
| 7p22.3-p22.2 (Dup) | 1 | 0 | 0 | 0 | 0 | 0 | 0 | 0 | 0 | 0 | 0 | 0 | 0 | 0 | 0 | 0 | 0 | 0 | 0 | 0 | 0 | 0 | 0 | 0 | 0 | 0 | | 0 | 0 | 0 | 0 |
| 7q11.22 (Del) | 0 | 0 | 0 | 0 | 0 | 0 | 0 | 0 | 0 | 0 | 0 | 0 | 0 | 1 | 0 | 0 | 0 | 0 | 0 | 0 | 1 | 0 | 0 | 0 | 0 | 0 | | 0 | 0 | 0 | 0 |
| 7q11.22 (Dup) | 0 | 0 | 0 | 0 | 0 | 0 | 0 | 0 | 1 | 0 | 0 | 0 | 0 | 0 | 0 | 0 | 0 | 0 | 0 | 0 | 0 | 0 | 0 | 0 | 0 | 0 | | 0 | 0 | 0 | 0 |
| 7q11.23 (Del) | 0 | 0 | 0 | 0 | 0 | 0 | 0 | 0 | 0 | 0 | 1 | 0 | 0 | 0 | 0 | 0 | 0 | 0 | 0 | 0 | 0 | 1 | 0 | 0 | 0 | 0 | | 0 | 0 | 0 | 0 |
| 7q11.23 (Dup) | 0 | 0 | 0 | 0 | 0 | 0 | 0 | 0 | 0 | 0 | 0 | 0 | 0 | 0 | 0 | 1 | 1 | 0 | 0 | 0 | 0 | 1 | 0 | 0 | 0 | 1 | | 0 | 0 | 0 | 0 |
| 7q21.13 (Dup) | 0 | 0 | 0 | 0 | 0 | 0 | 0 | 0 | 0 | 0 | 0 | 0 | 0 | 0 | 0 | 0 | 0 | 0 | 0 | 0 | 0 | 0 | 0 | 1 | 0 | 0 | | 0 | 0 | 0 | 0 |
| 7q21.3 (Del) | 0 | 0 | 0 | 0 | 0 | 0 | 0 | 0 | 0 | 0 | 0 | 0 | 0 | 0 | 0 | 0 | 0 | 0 | 0 | 2 | 0 | 0 | 0 | 0 | 0 | 0 | | 0 | 0 | 0 | 0 |
| 7q21.3 (Dup) | 0 | 0 | 1 | 0 | 0 | 0 | 0 | 0 | 0 | 0 | 0 | 0 | 0 | 0 | 0 | 0 | 0 | 0 | 0 | 0 | 0 | 0 | 0 | 0 | 0 | 0 | | 0 | 0 | 0 | 0 |
| 7q22.1 (Del) | 0 | 0 | 0 | 0 | 3 | 0 | 0 | 0 | 0 | 0 | 0 | 0 | 0 | 0 | 0 | 0 | 0 | 0 | 0 | 0 | 0 | 0 | 0 | 1 | 0 | 0 | | 0 | 0 | 0 | 0 |
| 7q22.1 (Dup) | 0 | 0 | 0 | 0 | 2 | 0 | 0 | 0 | 0 | 0 | 0 | 0 | 0 | 0 | 0 | 0 | 0 | 0 | 0 | 0 | 0 | 0 | 0 | 0 | 0 | 0 | | 0 | 0 | 0 | 0 |
| 7q22.1-q22.2 (Del) | 1 | 0 | 0 | 0 | 0 | 0 | 0 | 0 | 0 | 0 | 0 | 0 | 0 | 0 | 0 | 0 | 0 | 0 | 0 | 0 | 0 | 0 | 0 | 0 | 0 | 0 | | 0 | 0 | 0 | 0 |
| 7q31.1 (Del) | 0 | 0 | 0 | 0 | 0 | 0 | 0 | 0 | 0 | 0 | 0 | 0 | 0 | 0 | 0 | 0 | 0 | 1 | 0 | 0 | 0 | 0 | 0 | 2 | 0 | 0 | | 0 | 0 | 0 | 0 |
| 7q31.2 (Dup) | 0 | 0 | 0 | 0 | 0 | 1 | 0 | 0 | 0 | 0 | 0 | 0 | 0 | 0 | 0 | 0 | 0 | 0 | 0 | 0 | 0 | 0 | 0 | 0 | 0 | 0 | | 0 | 0 | 0 | 0 |
| 7q31.31 (Dup) | 0 | 0 | 0 | 0 | 0 | 1 | 0 | 0 | 0 | 0 | 0 | 0 | 0 | 0 | 0 | 0 | 0 | 0 | 0 | 0 | 0 | 0 | 0 | 0 | 0 | 0 | | 0 | 0 | 0 | 0 |
| 7q31.33 (Dup) | 0 | 0 | 0 | 0 | 0 | 0 | 0 | 0 | 0 | 0 | 0 | 0 | 0 | 0 | 0 | 0 | 0 | 0 | 0 | 0 | 0 | 0 | 0 | 0 | 5 | 0 | | 0 | 0 | 0 | 0 |
| 7q31.33-q32.1 (Del) | 0 | 0 | 0 | 0 | 0 | 0 | 0 | 0 | 0 | 0 | 0 | 0 | 0 | 0 | 0 | 0 | 0 | 0 | 0 | 0 | 0 | 0 | 0 | 0 | 0 | 3 | | 0 | 0 | 0 | 0 |
| 7q32.1 (Dup) | 0 | 0 | 0 | 0 | 0 | 1 | 0 | 0 | 0 | 0 | 0 | 0 | 0 | 0 | 0 | 0 | 0 | 0 | 0 | 0 | 0 | 0 | 0 | 0 | 2 | 0 | | 0 | 0 | 0 | 0 |
| 7q32.3 (Del) | 0 | 0 | 0 | 0 | 0 | 0 | 0 | 0 | 0 | 0 | 0 | 0 | 0 | 0 | 0 | 0 | 0 | 0 | 0 | 0 | 0 | 0 | 0 | 0 | 1 | 0 | | 0 | 0 | 0 | 0 |
| 7q32.3 (Dup) | 0 | 0 | 0 | 0 | 0 | 0 | 0 | 0 | 0 | 0 | 0 | 0 | 0 | 0 | 0 | 0 | 0 | 0 | 0 | 0 | 0 | 0 | 0 | 0 | 1 | 0 | | 0 | 0 | 0 | 0 |
| 7q35 (Del) | 0 | 0 | 0 | 0 | 0 | 0 | 0 | 1 | 1 | 1 | 0 | 0 | 0 | 0 | 0 | 0 | 0 | 0 | 0 | 0 | 0 | 0 | 0 | 0 | 0 | 0 | | 0 | 0 | 0 | 0 |
| 7q35 (Dup) | 0 | 0 | 0 | 0 | 0 | 1 | 0 | 0 | 0 | 0 | 0 | 0 | 0 | 0 | 0 | 0 | 0 | 0 | 0 | 0 | 0 | 0 | 0 | 3 | 0 | 0 | | 0 | 0 | 0 | 0 |
| 7q35-q36.3 (Del) | 0 | 0 | 0 | 0 | 0 | 0 | 0 | 0 | 0 | 0 | 0 | 0 | 0 | 0 | 0 | 0 | 0 | 0 | 0 | 0 | 0 | 0 | 0 | 0 | 0 | 2 | | 0 | 0 | 0 | 0 |
| 7q36.1 (Dup) | 0 | 0 | 0 | 0 | 0 | 0 | 0 | 0 | 0 | 1 | 0 | 0 | 0 | 0 | 0 | 0 | 0 | 0 | 0 | 0 | 0 | 0 | 0 | 0 | 0 | 0 | | 0 | 0 | 0 | 0 |
| 7q36.1-q36.2 (Dup) | 0 | 0 | 0 | 0 | 0 | 0 | 0 | 0 | 0 | 0 | 0 | 0 | 0 | 0 | 0 | 0 | 0 | 0 | 0 | 0 | 0 | 0 | 0 | 0 | 0 | 1 | | 0 | 0 | 0 | 0 |
| 7q36.1-q36.3 (Del) | 0 | 0 | 0 | 0 | 0 | 0 | 0 | 0 | 0 | 0 | 0 | 0 | 0 | 0 | 0 | 1 | 0 | 0 | 0 | 0 | 0 | 0 | 0 | 0 | 0 | 0 | | 0 | 0 | 0 | 0 |
| 7q36.2 (Del) | 0 | 0 | 0 | 0 | 0 | 0 | 1 | 0 | 1 | 0 | 0 | 0 | 0 | 0 | 0 | 0 | 0 | 0 | 1 | 0 | 0 | 0 | 0 | 2 | 0 | 0 | | 0 | 0 | 0 | 0 |
| 7q36.2 (Dup) | 0 | 0 | 0 | 0 | 0 | 0 | 0 | 0 | 1 | 0 | 0 | 0 | 0 | 0 | 0 | 0 | 0 | 0 | 3 | 0 | 0 | 0 | 0 | 1 | 0 | 1 | | 0 | 0 | 0 | 0 |
| 7q36.3 (Dup) | 0 | 0 | 0 | 0 | 0 | 0 | 1 | 0 | 0 | 0 | 0 | 0 | 0 | 0 | 0 | 0 | 0 | 0 | 0 | 0 | 0 | 0 | 0 | 0 | 0 | 0 | | 0 | 0 | 0 | 0 |
| 8p11.21 (Del) | 0 | 0 | 0 | 0 | 0 | 0 | 0 | 0 | 0 | 0 | 0 | 0 | 0 | 0 | 0 | 0 | 0 | 0 | 0 | 0 | 0 | 0 | 0 | 0 | 1 | 0 | | 0 | 0 | 0 | 0 |
| 8p11.21 (Dup) | 0 | 0 | 0 | 0 | 0 | 0 | 0 | 0 | 0 | 0 | 0 | 0 | 0 | 0 | 0 | 0 | 0 | 0 | 0 | 0 | 0 | 0 | 0 | 0 | 1 | 0 | | 0 | 0 | 0 | 0 |
| 8p11.22 (Del) | 0 | 1 | 0 | 0 | 0 | 0 | 0 | 0 | 0 | 0 | 0 | 0 | 0 | 0 | 0 | 0 | 0 | 0 | 0 | 0 | 0 | 0 | 0 | 0 | 0 | 0 | | 0 | 0 | 0 | 0 |
| 8p21.3 (Dup) | 0 | 0 | 0 | 0 | 0 | 0 | 1 | 0 | 0 | 0 | 0 | 0 | 0 | 0 | 0 | 0 | 0 | 0 | 0 | 0 | 0 | 0 | 0 | 0 | 0 | 0 | | 0 | 0 | 0 | 0 |
| 8p22 (Del) | 0 | 0 | 0 | 0 | 0 | 1 | 1 | 0 | 0 | 0 | 0 | 0 | 0 | 0 | 0 | 0 | 0 | 0 | 0 | 0 | 0 | 0 | 0 | 0 | 0 | 0 | | 0 | 0 | 0 | 0 |
| 8p23.1 (Del) | 0 | 0 | 0 | 0 | 0 | 0 | 0 | 0 | 0 | 0 | 0 | 0 | 0 | 0 | 0 | 0 | 0 | 0 | 0 | 0 | 0 | 1 | 0 | 2 | 0 | 0 | | 0 | 0 | 1 | 0 |
| 8p23.1 (Dup) | 0 | 0 | 0 | 0 | 0 | 0 | 0 | 0 | 0 | 1 | 0 | 0 | 0 | 0 | 0 | 0 | 0 | 0 | 0 | 0 | 0 | 1 | 0 | 0 | 0 | 0 | | 9 | 0 | 0 | 0 |
| 8p23.2 (Del) | 0 | 0 | 0 | 0 | 0 | 0 | 0 | 0 | 0 | 0 | 0 | 0 | 0 | 0 | 0 | 0 | 0 | 0 | 0 | 0 | 0 | 0 | 0 | 1 | 0 | 0 | | 0 | 0 | 0 | 0 |
| 8p23.2 (Dup) | 0 | 0 | 0 | 0 | 0 | 1 | 1 | 0 | 0 | 0 | 0 | 0 | 0 | 0 | 0 | 0 | 0 | 0 | 0 | 0 | 0 | 0 | 0 | 0 | 0 | 0 | | 0 | 0 | 0 | 0 |
| 8p23.3 (Dup) | 0 | 0 | 0 | 0 | 0 | 1 | 0 | 0 | 0 | 0 | 0 | 0 | 0 | 0 | 0 | 0 | 0 | 0 | 0 | 0 | 0 | 0 | 0 | 1 | 0 | 0 | | 0 | 0 | 0 | 0 |
| 8p23.3-p23.2 (Dup) | 0 | 0 | 0 | 0 | 0 | 0 | 0 | 0 | 0 | 0 | 0 | 0 | 0 | 0 | 1 | 0 | 0 | 0 | 0 | 0 | 0 | 0 | 0 | 0 | 0 | 0 | | 0 | 0 | 0 | 0 |
| 8p23.3-p22 (Del) | 0 | 0 | 0 | 0 | 0 | 0 | 0 | 0 | 0 | 0 | 0 | 0 | 0 | 0 | 1 | 0 | 0 | 0 | 0 | 0 | 0 | 0 | 0 | 0 | 0 | 0 | | 0 | 0 | 0 | 0 |
| 8p23.3-p23.1 (Del) | 0 | 0 | 0 | 0 | 0 | 0 | 0 | 0 | 0 | 0 | 1 | 0 | 0 | 0 | 0 | 0 | 0 | 0 | 0 | 0 | 0 | 0 | 0 | 0 | 0 | 0 | | 0 | 0 | 0 | 0 |
| 8q11.21 (Del) | 0 | 1 | 0 | 0 | 0 | 0 | 0 | 0 | 0 | 0 | 0 | 0 | 0 | 0 | 0 | 0 | 0 | 0 | 0 | 0 | 0 | 0 | 0 | 0 | 0 | 0 | | 0 | 0 | 0 | 0 |
| 8q11.21 (Dup) | 0 | 0 | 0 | 0 | 0 | 0 | 0 | 0 | 0 | 0 | 0 | 0 | 0 | 0 | 0 | 0 | 0 | 0 | 0 | 0 | 0 | 0 | 0 | 1 | 0 | 0 | | 0 | 0 | 0 | 0 |
| 8q11.23 (Dup) | 0 | 0 | 0 | 0 | 0 | 0 | 0 | 0 | 0 | 0 | 0 | 0 | 0 | 0 | 0 | 0 | 0 | 0 | 2 | 0 | 0 | 0 | 0 | 0 | 0 | 0 | | 0 | 0 | 0 | 0 |
| 8q12.3-q13.1 (Del) | 0 | 0 | 0 | 0 | 0 | 0 | 0 | 0 | 0 | 0 | 0 | 0 | 0 | 0 | 0 | 0 | 0 | 0 | 0 | 0 | 0 | 0 | 0 | 1 | 0 | 0 | | 0 | 0 | 0 | 0 |
| 8q13.3 (Del) | 0 | 0 | 0 | 0 | 0 | 0 | 0 | 0 | 0 | 0 | 0 | 0 | 0 | 0 | 0 | 0 | 0 | 0 | 0 | 0 | 0 | 0 | 0 | 1 | 0 | 0 | | 0 | 0 | 0 | 0 |
| 8q21.11 (Dup) | 0 | 0 | 0 | 0 | 0 | 1 | 0 | 0 | 0 | 0 | 0 | 0 | 0 | 0 | 0 | 0 | 0 | 0 | 0 | 0 | 0 | 0 | 0 | 0 | 0 | 0 | | 0 | 0 | 0 | 0 |
| 8q22.3 (Del) | 0 | 0 | 0 | 0 | 0 | 0 | 0 | 0 | 0 | 0 | 0 | 0 | 0 | 0 | 0 | 0 | 0 | 0 | 0 | 0 | 0 | 0 | 0 | 1 | 0 | 0 | | 0 | 0 | 0 | 0 |
| 8q23.3 (Dup) | 0 | 0 | 0 | 0 | 0 | 0 | 0 | 0 | 0 | 0 | 0 | 0 | 0 | 0 | 1 | 0 | 0 | 0 | 0 | 0 | 0 | 0 | 0 | 0 | 0 | 0 | | 0 | 0 | 0 | 0 |
| 8q24.22-q24.3 (Dup) | 0 | 0 | 0 | 0 | 0 | 0 | 0 | 0 | 0 | 0 | 0 | 0 | 0 | 0 | 0 | 0 | 0 | 1 | 0 | 0 | 0 | 0 | 0 | 0 | 0 | 0 | | 0 | 0 | 0 | 0 |
| 8q24.23 (Del) | 0 | 0 | 0 | 0 | 0 | 1 | 0 | 0 | 0 | 0 | 0 | 0 | 0 | 0 | 0 | 0 | 0 | 0 | 0 | 0 | 0 | 0 | 0 | 0 | 0 | 0 | | 0 | 0 | 0 | 0 |
| 8q24.3 (Del) | 0 | 0 | 0 | 0 | 0 | 0 | 0 | 0 | 0 | 0 | 0 | 0 | 0 | 0 | 0 | 0 | 0 | 0 | 0 | 0 | 0 | 0 | 0 | 0 | 2 | 0 | | 0 | 0 | 0 | 0 |
| 9p21.1 (Del) | 0 | 0 | 0 | 0 | 0 | 0 | 0 | 0 | 0 | 0 | 0 | 0 | 2 | 0 | 0 | 0 | 0 | 0 | 0 | 5 | 0 | 0 | 0 | 0 | 0 | 0 | | 0 | 0 | 0 | 0 |
| 9p21.3 (Dup) | 0 | 0 | 0 | 0 | 0 | 1 | 0 | 0 | 0 | 0 | 0 | 0 | 0 | 0 | 0 | 0 | 0 | 0 | 0 | 0 | 0 | 0 | 0 | 0 | 0 | 0 | | 0 | 0 | 0 | 0 |
| 9p23 (Del) | 0 | 0 | 0 | 0 | 0 | 0 | 0 | 0 | 0 | 0 | 0 | 0 | 0 | 0 | 0 | 0 | 0 | 0 | 0 | 0 | 0 | 0 | 0 | 1 | 0 | 0 | | 0 | 0 | 0 | 0 |
| 9p24 (Del) | 0 | 0 | 0 | 0 | 0 | 0 | 0 | 0 | 0 | 0 | 0 | 0 | 0 | 0 | 0 | 0 | 0 | 0 | 0 | 0 | 0 | 0 | 1 | 0 | 0 | 0 | | 0 | 0 | 0 | 0 |
| 9p24.1 (Del) | 0 | 0 | 0 | 0 | 0 | 0 | 0 | 0 | 0 | 0 | 0 | 0 | 0 | 0 | 0 | 0 | 0 | 0 | 2 | 0 | 0 | 0 | 0 | 0 | 0 | 0 | | 0 | 0 | 0 | 0 |
| 9p24.1-p24.2 (Dup) | 0 | 0 | 0 | 1 | 0 | 0 | 0 | 0 | 0 | 0 | 0 | 0 | 0 | 0 | 0 | 0 | 0 | 0 | 0 | 0 | 0 | 0 | 0 | 0 | 0 | 0 | | 0 | 0 | 0 | 0 |
| 9p24.2 (Dup) | 0 | 0 | 0 | 0 | 0 | 0 | 0 | 0 | 0 | 1 | 0 | 0 | 0 | 0 | 0 | 0 | 0 | 0 | 0 | 0 | 0 | 0 | 0 | 0 | 0 | 2 | | 0 | 0 | 0 | 0 |
| 9p24.2-p24.1 (Del) | 0 | 0 | 0 | 0 | 0 | 0 | 0 | 0 | 0 | 0 | 0 | 0 | 0 | 0 | 0 | 0 | 0 | 0 | 0 | 0 | 1 | 0 | 0 | 0 | 0 | 0 | | 0 | 0 | 0 | 0 |
| 9p24.3 (Del) | 0 | 0 | 0 | 0 | 0 | 0 | 1 | 0 | 0 | 0 | 0 | 0 | 0 | 0 | 0 | 0 | 0 | 0 | 0 | 0 | 0 | 0 | 0 | 3 | 0 | 0 | | 0 | 0 | 0 | 0 |
| 9p24.3 (Dup) | 0 | 0 | 0 | 0 | 0 | 0 | 0 | 0 | 1 | 0 | 0 | 0 | 0 | 0 | 0 | 0 | 0 | 0 | 0 | 0 | 0 | 0 | 0 | 3 | 0 | 0 | | 0 | 0 | 0 | 0 |
| 9p24.3-p24.2 (Del) | 0 | 0 | 0 | 0 | 0 | 0 | 0 | 0 | 0 | 0 | 0 | 0 | 0 | 0 | 0 | 0 | 0 | 0 | 0 | 0 | 0 | 0 | 0 | 1 | 0 | 0 | | 0 | 0 | 0 | 0 |
| 9q13-q21.31 (Del) | 0 | 0 | 0 | 1 | 0 | 0 | 0 | 0 | 0 | 0 | 0 | 0 | 0 | 0 | 0 | 0 | 0 | 0 | 0 | 0 | 0 | 0 | 0 | 0 | 0 | 0 | | 0 | 0 | 0 | 0 |
| 9q21.13 (Dup) | 0 | 1 | 0 | 0 | 0 | 0 | 0 | 0 | 0 | 0 | 0 | 0 | 0 | 0 | 0 | 0 | 0 | 0 | 0 | 0 | 0 | 0 | 0 | 0 | 0 | 0 | | 0 | 0 | 0 | 0 |
| 9q22.1 (Dup) | 0 | 0 | 0 | 0 | 0 | 0 | 0 | 0 | 0 | 1 | 0 | 0 | 0 | 0 | 0 | 0 | 0 | 0 | 0 | 0 | 0 | 0 | 0 | 0 | 0 | 0 | | 0 | 0 | 0 | 0 |
| 9q22.31 (Del) | 0 | 0 | 0 | 0 | 0 | 0 | 0 | 0 | 0 | 0 | 0 | 0 | 0 | 0 | 0 | 0 | 0 | 0 | 0 | 0 | 0 | 0 | 0 | 0 | 3 | 0 | | 0 | 0 | 0 | 0 |
| 9q32 (Del) | 0 | 0 | 0 | 0 | 0 | 1 | 0 | 0 | 0 | 0 | 0 | 0 | 0 | 0 | 0 | 0 | 0 | 0 | 0 | 0 | 0 | 0 | 0 | 0 | 0 | 0 | | 0 | 0 | 0 | 0 |
| 9q33.1 (Del) | 0 | 0 | 0 | 1 | 0 | 0 | 0 | 0 | 0 | 1 | 0 | 0 | 0 | 0 | 0 | 0 | 0 | 0 | 0 | 0 | 0 | 0 | 0 | 0 | 0 | 0 | | 0 | 0 | 0 | 0 |
| 9q33.1 (Dup) | 0 | 0 | 0 | 0 | 0 | 0 | 1 | 0 | 0 | 0 | 0 | 0 | 0 | 0 | 0 | 0 | 0 | 0 | 0 | 0 | 0 | 0 | 0 | 0 | 0 | 0 | | 0 | 0 | 0 | 0 |
| 9q33.2 (Dup) | 0 | 0 | 0 | 0 | 0 | 0 | 0 | 0 | 0 | 0 | 0 | 0 | 0 | 0 | 0 | 0 | 0 | 0 | 0 | 0 | 0 | 0 | 0 | 2 | 0 | 0 | | 0 | 0 | 0 | 0 |
| 9q33.2-q33.3 (Dup) | 0 | 0 | 0 | 0 | 0 | 1 | 0 | 0 | 0 | 0 | 0 | 0 | 0 | 0 | 0 | 0 | 0 | 0 | 0 | 0 | 0 | 0 | 0 | 0 | 0 | 0 | | 0 | 0 | 0 | 0 |
| 9q34.3 (Del) | 0 | 0 | 0 | 0 | 0 | 0 | 0 | 0 | 0 | 0 | 0 | 0 | 0 | 0 | 0 | 0 | 0 | 0 | 0 | 0 | 0 | 0 | 0 | 0 | 0 | 2 | | 0 | 0 | 0 | 0 |
| 9q34.3 (Dup) | 0 | 0 | 0 | 0 | 0 | 0 | 0 | 0 | 0 | 1 | 0 | 0 | 0 | 0 | 0 | 0 | 0 | 0 | 0 | 0 | 0 | 0 | 0 | 0 | 0 | 1 | | 0 | 0 | 0 | 0 |
| 10p11.21 (Dup) | 0 | 0 | 0 | 0 | 0 | 1 | 0 | 0 | 0 | 0 | 0 | 0 | 0 | 0 | 0 | 0 | 0 | 0 | 0 | 0 | 0 | 0 | 0 | 0 | 0 | 0 | | 0 | 0 | 0 | 0 |
| 10p12.33 (Del) | 0 | 0 | 0 | 0 | 0 | 0 | 0 | 0 | 0 | 0 | 0 | 0 | 1 | 0 | 0 | 0 | 0 | 0 | 0 | 0 | 0 | 0 | 0 | 0 | 0 | 0 | | 0 | 0 | 0 | 0 |
| 10p13 (Dup) | 0 | 0 | 0 | 0 | 0 | 0 | 0 | 0 | 0 | 0 | 0 | 1 | 0 | 0 | 0 | 0 | 0 | 0 | 0 | 0 | 0 | 0 | 0 | 0 | 0 | 0 | | 0 | 0 | 0 | 0 |
| 10p15.3 (Del) | 0 | 0 | 0 | 1 | 0 | 0 | 0 | 0 | 0 | 0 | 0 | 0 | 0 | 0 | 0 | 0 | 0 | 0 | 0 | 0 | 0 | 0 | 0 | 0 | 0 | 0 | | 0 | 0 | 0 | 0 |
| 10p15.3 (Dup) | 0 | 0 | 0 | 0 | 0 | 0 | 0 | 0 | 0 | 0 | 0 | 0 | 0 | 0 | 0 | 0 | 0 | 0 | 0 | 0 | 0 | 0 | 0 | 0 | 0 | 5 | | 0 | 0 | 0 | 0 |
| 10q11.2 (Dup) | 0 | 0 | 0 | 0 | 0 | 0 | 0 | 0 | 0 | 0 | 1 | 0 | 0 | 0 | 0 | 0 | 0 | 0 | 0 | 0 | 0 | 0 | 0 | 0 | 0 | 0 | | 0 | 0 | 0 | 0 |
| 10q11.21 (Dup) | 0 | 0 | 0 | 0 | 0 | 0 | 0 | 0 | 0 | 0 | 0 | 0 | 0 | 0 | 0 | 0 | 0 | 0 | 0 | 0 | 0 | 0 | 0 | 1 | 0 | 0 | | 0 | 0 | 0 | 0 |
| 10q11.22 (Del) | 0 | 0 | 0 | 0 | 0 | 0 | 0 | 0 | 0 | 0 | 0 | 0 | 0 | 0 | 0 | 0 | 0 | 0 | 0 | 0 | 1 | 0 | 0 | 0 | 0 | 0 | | 0 | 0 | 0 | 0 |
| 10q11.23 (Dup) | 0 | 0 | 0 | 0 | 0 | 0 | 0 | 0 | 0 | 0 | 0 | 0 | 0 | 1 | 0 | 0 | 0 | 0 | 0 | 0 | 0 | 0 | 0 | 0 | 0 | 0 | | 0 | 0 | 0 | 0 |
| 10q11.23-q21.2 (Dup) | 0 | 0 | 0 | 0 | 0 | 0 | 0 | 0 | 0 | 0 | 0 | 0 | 0 | 0 | 0 | 0 | 0 | 0 | 0 | 0 | 0 | 0 | 0 | 0 | 0 | 0 | | 0 | 1 | 0 | 0 |
| 10q12.33 (Dup) | 0 | 0 | 0 | 0 | 0 | 0 | 0 | 0 | 0 | 0 | 0 | 0 | 0 | 0 | 1 | 0 | 0 | 0 | 0 | 0 | 0 | 0 | 0 | 0 | 0 | 0 | | 0 | 0 | 0 | 0 |
| 10q21.1 (Del) | 0 | 0 | 0 | 0 | 0 | 0 | 0 | 0 | 0 | 0 | 0 | 0 | 0 | 0 | 0 | 0 | 0 | 0 | 0 | 0 | 0 | 0 | 0 | 0 | 0 | 0 | | 0 | 0 | 1 | 0 |
| 10q21.1 (Dup) | 0 | 0 | 0 | 0 | 0 | 0 | 0 | 0 | 0 | 0 | 0 | 0 | 0 | 0 | 0 | 0 | 0 | 0 | 0 | 0 | 0 | 0 | 0 | 1 | 0 | 0 | | 0 | 0 | 0 | 0 |
| 10q21.2-q21.3 (Del) | 0 | 0 | 0 | 0 | 0 | 0 | 0 | 0 | 0 | 0 | 0 | 0 | 0 | 0 | 0 | 0 | 0 | 0 | 0 | 0 | 0 | 0 | 0 | 0 | 0 | 1 | | 0 | 0 | 0 | 0 |
| 10q21.3 (Del) | 0 | 0 | 0 | 0 | 0 | 0 | 1 | 0 | 0 | 0 | 0 | 0 | 0 | 0 | 0 | 0 | 0 | 0 | 0 | 0 | 0 | 0 | 0 | 10 | 0 | 0 | | 0 | 0 | 0 | 0 |
| 10q21.3 (Dup) | 0 | 0 | 0 | 0 | 0 | 0 | 0 | 0 | 1 | 0 | 0 | 0 | 0 | 0 | 0 | 0 | 0 | 0 | 0 | 0 | 0 | 0 | 0 | 0 | 0 | 0 | | 0 | 0 | 0 | 0 |
| 10q23 (Del) | 0 | 0 | 0 | 0 | 0 | 0 | 0 | 0 | 0 | 0 | 0 | 0 | 0 | 4 | 0 | 0 | 0 | 0 | 0 | 0 | 0 | 1 | 0 | 0 | 0 | 0 | | 0 | 0 | 0 | 0 |
| 10q23 (Dup) | 0 | 0 | 0 | 0 | 0 | 0 | 0 | 0 | 0 | 0 | 0 | 0 | 0 | 8 | 0 | 0 | 0 | 0 | 0 | 0 | 0 | 0 | 0 | 0 | 0 | 0 | | 0 | 0 | 0 | 0 |
| 10q23.1 (Del) | 0 | 0 | 0 | 0 | 0 | 0 | 0 | 0 | 0 | 1 | 0 | 0 | 0 | 0 | 0 | 0 | 0 | 0 | 0 | 7 | 0 | 0 | 0 | 0 | 0 | 0 | | 0 | 0 | 0 | 0 |
| 10q23.2-q23.31 (Del) | 13 | 0 | 0 | 0 | 0 | 0 | 0 | 0 | 0 | 0 | 0 | 0 | 0 | 0 | 0 | 0 | 0 | 0 | 0 | 0 | 0 | 0 | 0 | 0 | 0 | 0 | | 0 | 0 | 0 | 0 |
| 10q23.31 (Dup) | 0 | 0 | 0 | 0 | 0 | 0 | 0 | 0 | 0 | 0 | 0 | 0 | 0 | 0 | 0 | 0 | 0 | 0 | 0 | 2 | 0 | 0 | 0 | 0 | 0 | 0 | | 0 | 0 | 0 | 0 |
| 10q24.32 (Dup) | 0 | 0 | 0 | 0 | 0 | 0 | 0 | 0 | 0 | 0 | 0 | 0 | 0 | 0 | 0 | 0 | 0 | 0 | 0 | 0 | 0 | 0 | 0 | 0 | 0 | 1 | | 0 | 0 | 0 | 0 |
| 10q26.2 (Dup) | 0 | 0 | 0 | 0 | 0 | 1 | 0 | 0 | 0 | 0 | 0 | 0 | 0 | 0 | 0 | 0 | 0 | 0 | 0 | 0 | 0 | 0 | 0 | 0 | 0 | 0 | | 0 | 0 | 0 | 0 |
| 10q26.2-q26.3 (Dup) | 1 | 0 | 0 | 0 | 0 | 0 | 0 | 0 | 0 | 0 | 0 | 0 | 0 | 0 | 0 | 0 | 0 | 0 | 0 | 0 | 0 | 0 | 0 | 0 | 0 | 0 | | 0 | 0 | 0 | 0 |
| 10q26.3 (Dup) | 0 | 0 | 1 | 0 | 0 | 0 | 0 | 0 | 0 | 0 | 0 | 0 | 0 | 0 | 0 | 0 | 0 | 0 | 0 | 0 | 0 | 0 | 0 | 2 | 0 | 0 | | 0 | 0 | 0 | 0 |
| 11p11.2 (Dup) | 0 | 0 | 0 | 0 | 0 | 0 | 0 | 0 | 0 | 0 | 0 | 0 | 0 | 0 | 0 | 0 | 0 | 1 | 0 | 0 | 0 | 0 | 0 | 0 | 0 | 0 | | 0 | 0 | 0 | 0 |
| 11p12 (Del) | 0 | 0 | 0 | 0 | 0 | 0 | 0 | 0 | 0 | 0 | 0 | 0 | 0 | 0 | 0 | 0 | 0 | 0 | 2 | 0 | 0 | 0 | 0 | 0 | 0 | 0 | | 0 | 0 | 0 | 0 |
| 11p14.1 (Del) | 0 | 0 | 0 | 0 | 0 | 0 | 0 | 0 | 1 | 0 | 0 | 0 | 0 | 0 | 0 | 0 | 0 | 0 | 0 | 0 | 0 | 0 | 0 | 0 | 0 | 0 | | 0 | 0 | 0 | 0 |
| 11p14.3 (Dup) | 0 | 0 | 0 | 0 | 0 | 0 | 1 | 0 | 0 | 0 | 0 | 0 | 0 | 0 | 0 | 0 | 0 | 0 | 0 | 0 | 0 | 0 | 0 | 0 | 0 | 0 | | 0 | 0 | 0 | 0 |
| 11p15.4 (Dup) | 0 | 0 | 0 | 0 | 0 | 0 | 0 | 0 | 0 | 0 | 0 | 0 | 0 | 0 | 0 | 0 | 0 | 0 | 0 | 0 | 0 | 0 | 0 | 1 | 0 | 0 | | 0 | 0 | 0 | 0 |
| 11p15.5 (Dup) | 7 | 0 | 0 | 0 | 0 | 0 | 0 | 0 | 0 | 0 | 0 | 0 | 0 | 0 | 0 | 0 | 0 | 0 | 0 | 0 | 0 | 0 | 0 | 0 | 0 | 0 | | 0 | 0 | 0 | 0 |
| 11q12.1-q12.1 (Dup) | 0 | 0 | 0 | 0 | 0 | 0 | 0 | 0 | 0 | 0 | 0 | 0 | 0 | 0 | 0 | 0 | 0 | 0 | 0 | 0 | 0 | 0 | 0 | 0 | 0 | 2 | | 0 | 0 | 0 | 0 |
| 11q12.1-q12.2 (Dup) | 0 | 0 | 0 | 0 | 0 | 0 | 0 | 0 | 0 | 0 | 0 | 0 | 0 | 0 | 0 | 0 | 0 | 1 | 0 | 0 | 0 | 0 | 0 | 0 | 0 | 0 | | 0 | 0 | 0 | 0 |
| 11q12.2 (Del) | 0 | 0 | 0 | 0 | 1 | 0 | 0 | 0 | 0 | 0 | 0 | 0 | 0 | 0 | 0 | 0 | 0 | 0 | 0 | 0 | 0 | 0 | 0 | 0 | 0 | 0 | | 0 | 0 | 0 | 0 |
| 11q12.2 (Dup) | 0 | 0 | 0 | 0 | 1 | 0 | 0 | 0 | 0 | 0 | 0 | 0 | 0 | 0 | 0 | 0 | 0 | 0 | 0 | 0 | 0 | 0 | 0 | 0 | 0 | 0 | | 0 | 0 | 0 | 0 |
| 11q13.3 (Del) | 0 | 0 | 0 | 0 | 0 | 0 | 0 | 0 | 0 | 0 | 0 | 0 | 0 | 0 | 0 | 0 | 0 | 0 | 0 | 0 | 0 | 0 | 0 | 1 | 0 | 0 | | 0 | 0 | 0 | 0 |
| 11q13.3-q13.4 (Del) | 0 | 0 | 0 | 0 | 0 | 0 | 0 | 0 | 0 | 0 | 0 | 0 | 0 | 0 | 0 | 0 | 0 | 0 | 0 | 0 | 0 | 0 | 0 | 1 | 0 | 0 | | 0 | 0 | 0 | 0 |
| 11q14.1 (Del) | 0 | 0 | 1 | 0 | 0 | 0 | 0 | 0 | 0 | 0 | 0 | 0 | 0 | 0 | 0 | 0 | 0 | 0 | 0 | 0 | 0 | 0 | 0 | 0 | 0 | 0 | | 0 | 0 | 0 | 0 |
| 11q14.1 (Dup) | 0 | 0 | 0 | 0 | 0 | 0 | 0 | 0 | 1 | 0 | 0 | 0 | 0 | 0 | 0 | 0 | 0 | 0 | 0 | 0 | 0 | 0 | 0 | 0 | 0 | 0 | | 0 | 0 | 0 | 0 |
| 11q22.1 (Dup) | 0 | 0 | 0 | 0 | 0 | 0 | 0 | 0 | 0 | 0 | 0 | 0 | 0 | 0 | 0 | 0 | 0 | 0 | 0 | 0 | 0 | 0 | 0 | 1 | 0 | 0 | | 0 | 0 | 0 | 0 |
| 11q23.3 (Del) | 0 | 0 | 0 | 0 | 0 | 0 | 0 | 0 | 0 | 0 | 0 | 0 | 0 | 0 | 0 | 0 | 0 | 0 | 0 | 0 | 1 | 0 | 0 | 0 | 0 | 0 | | 0 | 0 | 0 | 0 |
| 11q23.3 (Dup) | 0 | 0 | 0 | 0 | 0 | 0 | 0 | 0 | 0 | 0 | 0 | 0 | 0 | 0 | 0 | 0 | 0 | 0 | 0 | 0 | 0 | 0 | 0 | 1 | 0 | 0 | | 0 | 0 | 0 | 0 |
| 11q24.2-q25 (Del) | 0 | 0 | 0 | 0 | 0 | 0 | 0 | 0 | 0 | 0 | 0 | 0 | 0 | 0 | 0 | 0 | 0 | 0 | 0 | 0 | 0 | 0 | 0 | 1 | 0 | 0 | | 0 | 0 | 0 | 0 |
| 11q24.3-q25 (Del) | 0 | 0 | 0 | 0 | 0 | 0 | 0 | 0 | 0 | 0 | 0 | 0 | 0 | 0 | 0 | 0 | 0 | 0 | 0 | 0 | 0 | 0 | 0 | 0 | 0 | 5 | | 0 | 0 | 0 | 0 |
| 11q25 (Dup) | 3 | 0 | 0 | 0 | 0 | 0 | 0 | 0 | 0 | 0 | 0 | 0 | 0 | 0 | 0 | 0 | 0 | 0 | 0 | 0 | 0 | 0 | 0 | 0 | 0 | 4 | | 0 | 0 | 0 | 0 |
| 12p11.1 (Del) | 0 | 0 | 0 | 0 | 0 | 0 | 0 | 0 | 0 | 0 | 0 | 0 | 0 | 0 | 0 | 0 | 0 | 0 | 0 | 0 | 0 | 0 | 0 | 0 | 0 | 0 | | 15 | 0 | 0 | 0 |
| 12p11.1 (Dup) | 0 | 0 | 0 | 0 | 0 | 2 | 1 | 0 | 0 | 0 | 0 | 0 | 0 | 0 | 0 | 0 | 0 | 0 | 0 | 0 | 0 | 0 | 0 | 0 | 0 | 0 | | 0 | 0 | 0 | 0 |
| 12p11.21 (Del) | 0 | 0 | 0 | 0 | 3 | 0 | 0 | 0 | 0 | 0 | 0 | 0 | 0 | 0 | 0 | 0 | 0 | 0 | 0 | 0 | 0 | 0 | 0 | 0 | 0 | 0 | | 0 | 0 | 0 | 0 |
| 12p11.21 (Dup) | 0 | 0 | 0 | 0 | 1 | 0 | 0 | 0 | 0 | 0 | 0 | 0 | 0 | 0 | 0 | 0 | 0 | 0 | 0 | 0 | 0 | 0 | 0 | 0 | 0 | 0 | | 0 | 0 | 0 | 0 |
| 12p12.1 (Del) | 0 | 0 | 0 | 0 | 0 | 0 | 0 | 0 | 0 | 0 | 0 | 0 | 0 | 0 | 0 | 0 | 0 | 0 | 0 | 0 | 0 | 0 | 0 | 0 | 0 | 1 | | 0 | 0 | 0 | 0 |
| 12p12.1-p11.23 (Del) | 6 | 0 | 0 | 0 | 0 | 0 | 0 | 0 | 0 | 0 | 0 | 0 | 0 | 0 | 0 | 0 | 0 | 0 | 0 | 0 | 0 | 0 | 0 | 0 | 0 | 0 | | 0 | 0 | 0 | 0 |
| 12p13.2 (Del) | 0 | 0 | 0 | 0 | 0 | 0 | 0 | 0 | 0 | 0 | 0 | 0 | 0 | 0 | 0 | 0 | 0 | 0 | 0 | 0 | 0 | 0 | 0 | 1 | 0 | 0 | | 0 | 0 | 0 | 0 |
| 12p13.31 (Del) | 0 | 0 | 0 | 0 | 0 | 0 | 0 | 0 | 0 | 0 | 0 | 0 | 0 | 0 | 0 | 0 | 0 | 0 | 0 | 0 | 0 | 0 | 0 | 1 | 0 | 0 | | 0 | 0 | 0 | 0 |
| 12p13.31 (Dup) | 0 | 0 | 0 | 1 | 0 | 0 | 0 | 0 | 0 | 0 | 0 | 0 | 0 | 0 | 0 | 0 | 0 | 0 | 0 | 0 | 0 | 0 | 0 | 0 | 0 | 0 | | 0 | 0 | 1 | 0 |
| 12p13.32 (Dup) | 0 | 0 | 0 | 0 | 0 | 0 | 0 | 0 | 0 | 0 | 0 | 0 | 0 | 0 | 0 | 0 | 0 | 0 | 0 | 0 | 0 | 0 | 0 | 1 | 0 | 0 | | 0 | 0 | 0 | 0 |
| 12p13.33 (Del) | 0 | 0 | 0 | 0 | 0 | 0 | 0 | 0 | 0 | 0 | 0 | 0 | 0 | 0 | 0 | 0 | 0 | 0 | 0 | 0 | 0 | 0 | 0 | 5 | 0 | 0 | | 0 | 0 | 0 | 0 |
| 12q12 (Del) | 0 | 0 | 0 | 0 | 0 | 0 | 0 | 0 | 0 | 0 | 0 | 0 | 0 | 0 | 0 | 0 | 0 | 0 | 0 | 0 | 0 | 0 | 0 | 1 | 0 | 0 | | 0 | 0 | 0 | 0 |
| 12q13.3-q14.1 (Dup) | 0 | 0 | 0 | 0 | 0 | 0 | 0 | 0 | 0 | 0 | 0 | 0 | 0 | 0 | 0 | 0 | 0 | 0 | 0 | 0 | 0 | 0 | 0 | 1 | 0 | 0 | | 0 | 0 | 0 | 0 |
| 12q14.3 (Del) | 0 | 0 | 0 | 0 | 0 | 0 | 0 | 0 | 0 | 0 | 0 | 1 | 0 | 0 | 0 | 0 | 0 | 0 | 0 | 0 | 0 | 0 | 0 | 0 | 0 | 0 | | 0 | 0 | 0 | 0 |
| 12q21.31 (Del) | 0 | 0 | 0 | 0 | 0 | 0 | 1 | 0 | 0 | 0 | 0 | 0 | 0 | 0 | 0 | 0 | 0 | 0 | 0 | 0 | 0 | 0 | 0 | 0 | 0 | 0 | | 0 | 0 | 0 | 0 |
| 12q23.1 (Del) | 0 | 0 | 0 | 0 | 0 | 0 | 0 | 0 | 0 | 0 | 0 | 0 | 0 | 0 | 0 | 0 | 0 | 0 | 0 | 0 | 0 | 0 | 0 | 1 | 0 | 0 | | 0 | 0 | 0 | 0 |
| 12q23.2 (Dup) | 0 | 0 | 0 | 0 | 0 | 0 | 0 | 0 | 0 | 0 | 0 | 0 | 0 | 0 | 0 | 0 | 0 | 0 | 0 | 2 | 0 | 0 | 0 | 0 | 0 | 0 | | 0 | 0 | 0 | 0 |
| 12q24.33 (Dup) | 0 | 0 | 0 | 0 | 0 | 1 | 0 | 0 | 0 | 0 | 0 | 0 | 0 | 0 | 0 | 0 | 0 | 0 | 0 | 0 | 0 | 0 | 0 | 0 | 0 | 1 | | 0 | 0 | 0 | 0 |
| 13q13.3 (Del) | 0 | 0 | 0 | 0 | 0 | 0 | 0 | 0 | 0 | 0 | 0 | 0 | 0 | 0 | 0 | 0 | 0 | 0 | 0 | 1 | 0 | 0 | 0 | 0 | 0 | 0 | | 0 | 0 | 0 | 0 |
| 13q13.31-q13.33 (Dup) | 0 | 0 | 1 | 0 | 0 | 0 | 0 | 0 | 0 | 0 | 0 | 0 | 0 | 0 | 0 | 0 | 0 | 0 | 0 | 0 | 0 | 0 | 0 | 0 | 0 | 0 | | 0 | 0 | 0 | 0 |
| 13q14.12-q14.13 (Del) | 0 | 0 | 0 | 0 | 0 | 0 | 0 | 0 | 0 | 0 | 0 | 0 | 0 | 0 | 0 | 0 | 0 | 0 | 0 | 0 | 0 | 0 | 0 | 0 | 0 | 0 | | 0 | 1 | 0 | 0 |
| 13q21.1 (Dup) | 0 | 0 | 0 | 0 | 1 | 0 | 0 | 0 | 0 | 0 | 0 | 0 | 0 | 0 | 0 | 0 | 0 | 0 | 0 | 0 | 0 | 0 | 0 | 0 | 0 | 0 | | 0 | 0 | 0 | 0 |
| 13q21.2 (Del) | 0 | 0 | 0 | 0 | 0 | 0 | 0 | 0 | 0 | 1 | 0 | 0 | 0 | 0 | 0 | 0 | 0 | 0 | 0 | 0 | 0 | 0 | 0 | 0 | 0 | 0 | | 0 | 0 | 0 | 0 |
| 13q21.31 (Dup) | 0 | 0 | 0 | 0 | 0 | 0 | 0 | 0 | 0 | 0 | 0 | 0 | 0 | 0 | 0 | 0 | 0 | 0 | 0 | 0 | 0 | 0 | 0 | 1 | 0 | 0 | | 0 | 0 | 0 | 0 |
| 13q21.32 (Dup) | 0 | 0 | 0 | 0 | 0 | 0 | 0 | 0 | 0 | 0 | 0 | 0 | 0 | 0 | 0 | 0 | 0 | 0 | 2 | 0 | 0 | 0 | 0 | 0 | 0 | 0 | | 0 | 0 | 0 | 0 |
| 14q11.2 (Del) | 1 | 0 | 0 | 0 | 0 | 0 | 0 | 0 | 0 | 0 | 0 | 0 | 0 | 0 | 0 | 0 | 0 | 0 | 0 | 0 | 0 | 0 | 0 | 0 | 0 | 0 | | 0 | 0 | 0 | 0 |
| 14q11.2 (Dup) | 0 | 1 | 0 | 0 | 0 | 0 | 0 | 0 | 0 | 0 | 0 | 0 | 0 | 0 | 0 | 0 | 0 | 0 | 0 | 0 | 0 | 0 | 0 | 1 | 0 | 0 | | 0 | 0 | 0 | 0 |
| 14q21.1 (Del) | 0 | 1 | 0 | 0 | 0 | 0 | 0 | 0 | 0 | 0 | 0 | 0 | 0 | 0 | 0 | 0 | 0 | 0 | 0 | 0 | 0 | 0 | 0 | 0 | 0 | 0 | | 0 | 0 | 0 | 0 |
| 14q23.3 (Del) | 0 | 0 | 0 | 0 | 0 | 0 | 0 | 0 | 1 | 1 | 0 | 0 | 0 | 0 | 0 | 0 | 0 | 0 | 0 | 0 | 0 | 0 | 0 | 0 | 1 | 0 | | 0 | 0 | 0 | 0 |
| 14q24.3 (Del) | 0 | 0 | 0 | 0 | 7 | 0 | 0 | 0 | 0 | 0 | 0 | 0 | 0 | 0 | 0 | 0 | 0 | 0 | 0 | 0 | 0 | 0 | 0 | 0 | 1 | 0 | | 0 | 0 | 0 | 0 |
| 14q31.1 (Del) | 0 | 0 | 0 | 0 | 0 | 0 | 0 | 0 | 1 | 0 | 0 | 0 | 0 | 0 | 0 | 0 | 0 | 0 | 0 | 0 | 0 | 0 | 0 | 2 | 1 | 0 | | 0 | 0 | 0 | 0 |
| 14q32.2 (Dup) | 0 | 0 | 0 | 0 | 0 | 0 | 0 | 0 | 0 | 0 | 0 | 0 | 0 | 0 | 0 | 0 | 0 | 0 | 0 | 5 | 0 | 0 | 0 | 0 | 0 | 0 | | 0 | 0 | 0 | 0 |
| 14q32.31 (Del) | 0 | 0 | 0 | 0 | 0 | 0 | 0 | 0 | 0 | 0 | 0 | 0 | 0 | 0 | 0 | 0 | 0 | 0 | 0 | 2 | 0 | 0 | 0 | 0 | 0 | 0 | | 0 | 0 | 0 | 0 |
| 14q32.31 (Dup) | 0 | 0 | 0 | 0 | 0 | 0 | 0 | 0 | 0 | 0 | 0 | 0 | 0 | 0 | 0 | 0 | 0 | 0 | 0 | 196 | 0 | 0 | 0 | 0 | 0 | 0 | | 0 | 0 | 0 | 0 |
| 14q32.33 (Del) | 0 | 0 | 0 | 0 | 0 | 0 | 0 | 0 | 1 | 0 | 0 | 0 | 0 | 0 | 0 | 0 | 0 | 0 | 0 | 0 | 0 | 0 | 0 | 0 | 0 | 0 | | 0 | 0 | 0 | 0 |
| 15q11.1 (Del) | 0 | 0 | 0 | 0 | 1 | 0 | 0 | 0 | 0 | 0 | 0 | 0 | 0 | 0 | 0 | 0 | 0 | 0 | 0 | 0 | 0 | 0 | 0 | 0 | 0 | 0 | | 0 | 0 | 0 | 0 |
| 15q11.1-q11.2 (Del) | 0 | 0 | 0 | 0 | 1 | 0 | 0 | 0 | 0 | 0 | 0 | 0 | 0 | 0 | 0 | 0 | 0 | 0 | 0 | 0 | 0 | 0 | 0 | 0 | 0 | 0 | | 0 | 0 | 0 | 0 |
| 15q11.2 (Del) | 0 | 1 | 0 | 0 | 1 | 0 | 0 | 0 | 0 | 0 | 0 | 0 | 0 | 0 | 0 | 3 | 0 | 0 | 0 | 0 | 2 | 0 | 0 | 2 | 0 | 0 | | 0 | 0 | 1 | 0 |
| 15q11.2 (Dup) | 0 | 0 | 0 | 0 | 0 | 1 | 0 | 0 | 0 | 0 | 0 | 1 | 0 | 0 | 0 | 0 | 0 | 0 | 0 | 0 | 0 | 0 | 0 | 5 | 0 | 0 | | 0 | 0 | 0 | 0 |
| 15q11.2-q13.1 (Dup) | 0 | 0 | 0 | 0 | 0 | 1 | 0 | 0 | 0 | 0 | 0 | 0 | 0 | 0 | 2 | 2 | 0 | 0 | 0 | 11 | 0 | 0 | 0 | 0 | 0 | 0 | | 0 | 0 | 1 | 0 |
| 15q11.2-q12 (Dup) | 0 | 0 | 0 | 0 | 0 | 0 | 0 | 0 | 0 | 0 | 0 | 0 | 0 | 3 | 0 | 0 | 0 | 0 | 0 | 0 | 0 | 0 | 0 | 0 | 0 | 0 | | 0 | 0 | 0 | 0 |
| 15q11.2-q13 (Del) | 0 | 0 | 0 | 0 | 0 | 0 | 0 | 0 | 0 | 0 | 0 | 0 | 0 | 0 | 0 | 0 | 0 | 0 | 0 | 0 | 0 | 1 | 0 | 0 | 0 | 0 | | 0 | 0 | 0 | 0 |
| 15q11.2-q13 (Dup) | 0 | 0 | 0 | 0 | 0 | 0 | 0 | 0 | 0 | 0 | 0 | 0 | 0 | 0 | 0 | 0 | 0 | 0 | 0 | 0 | 0 | 1 | 0 | 0 | 0 | 0 | | 0 | 0 | 0 | 0 |
| 15q11.2-q13.3 (Dup) | 0 | 0 | 0 | 0 | 0 | 0 | 0 | 0 | 0 | 0 | 0 | 0 | 0 | 0 | 3 | 0 | 0 | 0 | 2 | 0 | 0 | 0 | 0 | 0 | 0 | 0 | | 0 | 0 | 0 | 0 |
| 15q11-q12 (Dup) | 0 | 0 | 0 | 0 | 0 | 0 | 0 | 0 | 0 | 0 | 0 | 0 | 0 | 0 | 0 | 0 | 0 | 0 | 0 | 0 | 0 | 0 | 1 | 0 | 0 | 0 | | 0 | 0 | 0 | 0 |
| 15q11-q13 (Dup) | 0 | 0 | 0 | 0 | 0 | 0 | 0 | 0 | 0 | 0 | 3 | 0 | 0 | 0 | 0 | 0 | 0 | 0 | 0 | 0 | 0 | 0 | 0 | 0 | 0 | 0 | | 0 | 0 | 0 | 1 |
| 15q11-q13.33 (Dup) | 0 | 0 | 0 | 0 | 0 | 0 | 0 | 0 | 0 | 0 | 0 | 0 | 0 | 0 | 0 | 0 | 0 | 0 | 0 | 0 | 0 | 0 | 0 | 0 | 0 | 0 | | 0 | 1 | 0 | 0 |
| 15q12 (Dup) | 0 | 0 | 0 | 0 | 0 | 0 | 0 | 0 | 0 | 0 | 0 | 0 | 0 | 0 | 0 | 0 | 0 | 0 | 0 | 0 | 0 | 0 | 0 | 1 | 0 | 0 | | 0 | 0 | 0 | 0 |
| 15q13 (Del) | 0 | 0 | 0 | 0 | 0 | 0 | 0 | 0 | 0 | 0 | 0 | 0 | 0 | 0 | 0 | 0 | 0 | 0 | 0 | 0 | 0 | 0 | 0 | 1 | 0 | 0 | | 0 | 0 | 0 | 0 |
| 15q13.1 (Dup) | 0 | 0 | 0 | 0 | 0 | 0 | 0 | 0 | 0 | 1 | 0 | 0 | 0 | 0 | 0 | 0 | 0 | 0 | 0 | 0 | 0 | 0 | 0 | 1 | 0 | 0 | | 0 | 0 | 0 | 0 |
| 15q13.1-q13.2 (Del) | 0 | 0 | 0 | 0 | 0 | 0 | 0 | 0 | 0 | 0 | 0 | 0 | 0 | 0 | 2 | 0 | 0 | 0 | 0 | 0 | 0 | 0 | 0 | 0 | 0 | 0 | | 0 | 0 | 0 | 0 |
| 15q13.1-q13.2 (Dup) | 0 | 0 | 0 | 0 | 0 | 0 | 0 | 0 | 0 | 0 | 0 | 0 | 0 | 0 | 0 | 1 | 0 | 0 | 0 | 0 | 0 | 0 | 0 | 1 | 0 | 0 | | 0 | 0 | 0 | 0 |
| 15q13.2 (Del) | 0 | 0 | 0 | 0 | 1 | 0 | 0 | 0 | 0 | 0 | 0 | 0 | 0 | 0 | 0 | 0 | 0 | 0 | 0 | 0 | 0 | 0 | 0 | 2 | 0 | 0 | | 0 | 0 | 0 | 0 |
| 15q13.2-q13.3 (Del) | 0 | 0 | 0 | 0 | 0 | 0 | 0 | 0 | 0 | 0 | 0 | 0 | 0 | 0 | 0 | 2 | 0 | 0 | 0 | 2 | 0 | 1 | 0 | 0 | 0 | 0 | | 0 | 0 | 0 | 0 |
| 15q13.2-q13.3 (Dup) | 0 | 0 | 0 | 1 | 0 | 0 | 0 | 0 | 0 | 0 | 0 | 0 | 0 | 0 | 0 | 0 | 0 | 0 | 0 | 0 | 0 | 1 | 0 | 0 | 0 | 0 | | 0 | 0 | 0 | 0 |
| 15q13.3 (Del) | 1 | 0 | 0 | 0 | 1 | 1 | 0 | 0 | 1 | 0 | 0 | 0 | 0 | 5 | 0 | 0 | 0 | 0 | 0 | 0 | 0 | 0 | 0 | 1 | 0 | 7 | | 0 | 0 | 0 | 3 |
| 15q13.3 (Dup) | 0 | 0 | 0 | 0 | 0 | 1 | 0 | 0 | 0 | 0 | 0 | 1 | 0 | 1 | 0 | 0 | 0 | 0 | 0 | 21 | 0 | 0 | 0 | 0 | 0 | 1 | | 0 | 0 | 0 | 0 |
| 15q14 (Del) | 0 | 1 | 0 | 0 | 0 | 0 | 0 | 0 | 0 | 0 | 0 | 0 | 0 | 0 | 0 | 0 | 0 | 0 | 0 | 0 | 0 | 0 | 0 | 1 | 0 | 0 | | 0 | 0 | 0 | 0 |
| 15q14 (Dup) | 0 | 0 | 0 | 0 | 0 | 0 | 0 | 0 | 0 | 0 | 0 | 0 | 0 | 0 | 1 | 0 | 0 | 0 | 0 | 0 | 0 | 0 | 0 | 0 | 0 | 0 | | 0 | 0 | 0 | 0 |
| 15q15.2 (Dup) | 0 | 0 | 0 | 0 | 0 | 0 | 1 | 0 | 0 | 0 | 0 | 0 | 0 | 0 | 0 | 0 | 0 | 0 | 0 | 0 | 0 | 0 | 0 | 0 | 0 | 0 | | 0 | 0 | 0 | 0 |
| 15q15.3 (Del) | 0 | 0 | 0 | 0 | 0 | 0 | 0 | 0 | 0 | 0 | 0 | 0 | 0 | 0 | 0 | 0 | 0 | 0 | 0 | 0 | 0 | 0 | 0 | 1 | 0 | 0 | | 0 | 0 | 0 | 0 |
| 15q22.31 (Dup) | 0 | 0 | 0 | 0 | 0 | 0 | 0 | 0 | 0 | 0 | 0 | 0 | 0 | 0 | 0 | 0 | 0 | 0 | 0 | 0 | 0 | 0 | 0 | 1 | 0 | 0 | | 0 | 0 | 0 | 0 |
| 15q23 (Del) | 0 | 0 | 0 | 0 | 0 | 0 | 0 | 0 | 0 | 0 | 0 | 0 | 0 | 0 | 1 | 0 | 0 | 0 | 0 | 0 | 0 | 0 | 0 | 0 | 0 | 0 | | 0 | 0 | 0 | 0 |
| 15q24 (Del) | 0 | 0 | 0 | 0 | 0 | 0 | 0 | 0 | 0 | 0 | 0 | 0 | 0 | 0 | 0 | 0 | 0 | 0 | 0 | 0 | 0 | 0 | 0 | 0 | 0 | 1 | | 0 | 0 | 0 | 0 |
| 15q24.1 (Del) | 0 | 0 | 0 | 0 | 0 | 0 | 0 | 0 | 0 | 0 | 0 | 0 | 0 | 2 | 0 | 0 | 0 | 0 | 0 | 0 | 0 | 0 | 0 | 0 | 0 | 0 | | 0 | 0 | 0 | 0 |
| 15q24.2 (Del) | 0 | 0 | 0 | 0 | 0 | 0 | 0 | 0 | 0 | 0 | 0 | 0 | 0 | 1 | 0 | 0 | 0 | 0 | 0 | 0 | 0 | 0 | 0 | 0 | 0 | 0 | | 0 | 0 | 0 | 0 |
| 15q24.3 (Del) | 0 | 0 | 0 | 0 | 0 | 0 | 0 | 0 | 0 | 0 | 0 | 0 | 0 | 0 | 0 | 0 | 0 | 0 | 0 | 0 | 0 | 0 | 0 | 1 | 0 | 0 | | 0 | 0 | 0 | 0 |
| 15q25.1 (Dup) | 0 | 0 | 0 | 0 | 0 | 0 | 0 | 0 | 0 | 0 | 0 | 0 | 0 | 0 | 0 | 0 | 0 | 0 | 0 | 0 | 0 | 0 | 0 | 0 | 3 | 0 | | 0 | 0 | 0 | 0 |
| 15q25.2-q26.1 (Del) | 0 | 0 | 0 | 0 | 0 | 0 | 0 | 0 | 0 | 0 | 0 | 0 | 0 | 0 | 0 | 0 | 0 | 1 | 0 | 0 | 0 | 0 | 0 | 0 | 0 | 0 | | 0 | 0 | 0 | 0 |
| 15q26.1 (Del) | 0 | 0 | 0 | 0 | 0 | 0 | 0 | 0 | 0 | 0 | 0 | 0 | 0 | 0 | 0 | 0 | 0 | 0 | 0 | 0 | 0 | 0 | 0 | 1 | 0 | 0 | | 0 | 0 | 0 | 0 |
| 16p11.2 (Del) | 0 | 0 | 0 | 0 | 0 | 0 | 0 | 0 | 1 | 3 | 1 | 0 | 1 | 1 | 0 | 1 | 3 | 1 | 2 | 0 | 0 | 2 | 0 | 2 | 0 | 0 | | 0 | 1 | 0 | 1 |
| 16p11.2 (Dup) | 0 | 0 | 0 | 2 | 1 | 1 | 0 | 0 | 0 | 1 | 0 | 0 | 0 | 1 | 1 | 4 | 2 | 0 | 2 | 0 | 2 | 2 | 4 | 2 | 0 | 0 | | 0 | 0 | 0 | 0 |
| 16p12.1 (Del) | 0 | 0 | 0 | 0 | 0 | 0 | 0 | 0 | 0 | 0 | 0 | 0 | 0 | 0 | 0 | 0 | 0 | 0 | 0 | 0 | 0 | 1 | 0 | 4 | 0 | 0 | | 0 | 0 | 0 | 1 |
| 16p12.1 (Dup) | 0 | 0 | 0 | 0 | 0 | 0 | 0 | 0 | 0 | 0 | 0 | 0 | 0 | 0 | 0 | 1 | 0 | 0 | 0 | 0 | 0 | 0 | 0 | 0 | 0 | 0 | | 0 | 0 | 0 | 0 |
| 16p12.2 (Del) | 0 | 0 | 0 | 0 | 0 | 0 | 0 | 0 | 0 | 0 | 0 | 0 | 0 | 0 | 0 | 0 | 0 | 0 | 1 | 0 | 0 | 0 | 0 | 0 | 0 | 3 | | 0 | 0 | 0 | 0 |
| 16p12.2 (Dup) | 0 | 0 | 0 | 0 | 0 | 0 | 0 | 0 | 0 | 0 | 0 | 0 | 0 | 0 | 0 | 0 | 0 | 0 | 1 | 0 | 0 | 0 | 0 | 0 | 0 | 2 | | 0 | 0 | 0 | 0 |
| 16p12.3 (Del) | 0 | 0 | 0 | 0 | 0 | 0 | 0 | 0 | 0 | 0 | 0 | 0 | 0 | 0 | 0 | 0 | 0 | 0 | 0 | 0 | 0 | 0 | 0 | 1 | 0 | 0 | | 0 | 0 | 0 | 0 |
| 16p12.3 (Dup) | 0 | 0 | 0 | 0 | 0 | 0 | 0 | 0 | 0 | 1 | 0 | 0 | 0 | 0 | 0 | 0 | 0 | 0 | 0 | 0 | 0 | 0 | 0 | 0 | 0 | 0 | | 0 | 0 | 0 | 0 |
| 16p13.1 (Del) | 0 | 0 | 2 | 0 | 0 | 0 | 0 | 0 | 0 | 0 | 0 | 0 | 0 | 0 | 0 | 0 | 0 | 0 | 0 | 0 | 0 | 0 | 0 | 0 | 0 | 0 | | 0 | 0 | 0 | 0 |
| 16p13.11 (Del) | 0 | 0 | 0 | 0 | 0 | 0 | 0 | 0 | 0 | 0 | 1 | 0 | 0 | 1 | 0 | 1 | 0 | 0 | 0 | 0 | 0 | 1 | 0 | 0 | 0 | 0 | | 0 | 0 | 0 | 0 |
| 16p13.11 (Dup) | 0 | 0 | 0 | 0 | 0 | 0 | 0 | 0 | 0 | 0 | 0 | 0 | 0 | 0 | 0 | 1 | 0 | 2 | 0 | 0 | 0 | 0 | 0 | 1 | 0 | 3 | | 0 | 0 | 0 | 0 |
| 16p13.12 (Dup) | 0 | 0 | 0 | 1 | 0 | 0 | 0 | 0 | 0 | 0 | 0 | 0 | 0 | 0 | 0 | 0 | 0 | 0 | 0 | 0 | 0 | 0 | 0 | 0 | 0 | 0 | | 0 | 0 | 0 | 0 |
| 16p13.2 (Dup) | 0 | 0 | 0 | 0 | 1 | 0 | 0 | 0 | 0 | 0 | 0 | 0 | 0 | 0 | 0 | 0 | 0 | 0 | 0 | 0 | 0 | 0 | 0 | 1 | 0 | 0 | | 0 | 0 | 0 | 0 |
| 16p13.3 (Del) | 0 | 0 | 0 | 0 | 0 | 0 | 0 | 0 | 0 | 0 | 0 | 0 | 0 | 0 | 0 | 0 | 0 | 0 | 0 | 0 | 0 | 0 | 0 | 0 | 2 | 0 | | 0 | 1 | 1 | 0 |
| 16p13.3 (Dup) | 5 | 0 | 0 | 1 | 0 | 1 | 0 | 0 | 0 | 0 | 0 | 0 | 2 | 0 | 0 | 0 | 0 | 0 | 0 | 0 | 0 | 0 | 0 | 0 | 0 | 0 | | 0 | 0 | 0 | 0 |
| 16q12.1 (Dup) | 0 | 0 | 0 | 0 | 0 | 0 | 0 | 0 | 1 | 0 | 0 | 0 | 0 | 0 | 0 | 0 | 0 | 0 | 0 | 0 | 0 | 0 | 0 | 0 | 0 | 0 | | 0 | 0 | 0 | 0 |
| 16q21 (Del) | 0 | 0 | 0 | 0 | 0 | 0 | 1 | 0 | 0 | 0 | 0 | 0 | 0 | 0 | 0 | 0 | 0 | 0 | 0 | 0 | 0 | 0 | 0 | 1 | 0 | 0 | | 0 | 0 | 0 | 0 |
| 16q21 (Dup) | 2 | 0 | 0 | 0 | 0 | 0 | 0 | 0 | 0 | 0 | 0 | 0 | 0 | 0 | 0 | 0 | 0 | 0 | 0 | 0 | 0 | 0 | 0 | 0 | 0 | 0 | | 0 | 0 | 0 | 0 |
| 16q22.1 (Del) | 0 | 0 | 0 | 0 | 1 | 0 | 0 | 0 | 0 | 0 | 0 | 0 | 0 | 0 | 0 | 0 | 0 | 0 | 0 | 0 | 0 | 0 | 0 | 0 | 0 | 0 | | 0 | 0 | 0 | 0 |
| 16q23.1 (Del) | 0 | 0 | 0 | 0 | 0 | 0 | 1 | 0 | 0 | 0 | 0 | 0 | 0 | 0 | 0 | 0 | 0 | 0 | 0 | 0 | 0 | 0 | 0 | 1 | 0 | 0 | | 0 | 0 | 0 | 0 |
| 16q23.1 (Dup) | 0 | 0 | 1 | 0 | 0 | 0 | 0 | 0 | 0 | 0 | 0 | 0 | 0 | 0 | 0 | 0 | 0 | 0 | 0 | 0 | 0 | 0 | 0 | 0 | 0 | 0 | | 0 | 0 | 0 | 0 |
| 16q23.2-q24.1 (Del) | 0 | 0 | 0 | 0 | 1 | 0 | 0 | 0 | 0 | 0 | 0 | 0 | 0 | 0 | 0 | 0 | 0 | 0 | 0 | 0 | 0 | 0 | 0 | 0 | 0 | 0 | | 0 | 0 | 0 | 0 |
| 16q23.3 (Del) | 0 | 0 | 0 | 0 | 0 | 0 | 1 | 0 | 1 | 0 | 0 | 0 | 0 | 0 | 0 | 0 | 0 | 0 | 0 | 0 | 0 | 0 | 0 | 0 | 0 | 0 | | 0 | 0 | 0 | 0 |
| 16q24.1 (Del) | 0 | 0 | 0 | 0 | 0 | 0 | 0 | 0 | 0 | 0 | 0 | 0 | 0 | 0 | 0 | 0 | 0 | 0 | 0 | 0 | 0 | 0 | 0 | 1 | 0 | 0 | | 0 | 0 | 0 | 0 |
| 17p11.2 (Del) | 0 | 0 | 0 | 0 | 0 | 0 | 0 | 0 | 0 | 0 | 0 | 0 | 0 | 0 | 0 | 1 | 0 | 0 | 0 | 0 | 0 | 1 | 0 | 0 | 0 | 0 | | 0 | 0 | 0 | 0 |
| 17p11.2 (Dup) | 0 | 0 | 0 | 1 | 0 | 0 | 0 | 0 | 0 | 1 | 0 | 0 | 0 | 0 | 0 | 1 | 0 | 0 | 0 | 0 | 0 | 1 | 0 | 0 | 0 | 1 | | 0 | 0 | 0 | 0 |
| 17p12 (Del) | 0 | 0 | 0 | 0 | 0 | 0 | 0 | 0 | 0 | 0 | 2 | 0 | 0 | 0 | 1 | 1 | 0 | 0 | 0 | 0 | 0 | 0 | 0 | 0 | 0 | 0 | | 0 | 0 | 0 | 0 |
| 17p12 (Dup) | 0 | 0 | 0 | 0 | 0 | 0 | 1 | 0 | 1 | 0 | 0 | 0 | 0 | 0 | 0 | 0 | 0 | 0 | 0 | 0 | 0 | 0 | 0 | 0 | 0 | 0 | | 0 | 0 | 0 | 0 |
| 17p13.1 (Del) | 0 | 0 | 0 | 0 | 0 | 0 | 1 | 0 | 0 | 0 | 0 | 0 | 0 | 0 | 0 | 0 | 0 | 0 | 0 | 0 | 0 | 0 | 0 | 0 | 2 | 0 | | 0 | 0 | 0 | 0 |
| 17p13.1 (Dup) | 0 | 0 | 0 | 1 | 0 | 0 | 0 | 0 | 0 | 0 | 0 | 0 | 0 | 0 | 0 | 0 | 0 | 0 | 0 | 0 | 0 | 0 | 0 | 0 | 0 | 0 | | 0 | 0 | 0 | 0 |
| 17p13.2 (Del) | 0 | 0 | 0 | 1 | 0 | 0 | 0 | 0 | 0 | 0 | 0 | 0 | 0 | 0 | 0 | 0 | 0 | 0 | 0 | 0 | 0 | 0 | 0 | 0 | 0 | 0 | | 0 | 0 | 0 | 0 |
| 17p13.2 (Dup) | 0 | 0 | 0 | 0 | 0 | 0 | 0 | 0 | 0 | 0 | 0 | 0 | 1 | 0 | 0 | 0 | 0 | 0 | 0 | 0 | 0 | 0 | 0 | 0 | 0 | 0 | | 0 | 0 | 0 | 0 |
| 17p13.3 (Del) | 0 | 0 | 0 | 0 | 0 | 1 | 0 | 0 | 0 | 0 | 0 | 0 | 0 | 0 | 0 | 0 | 0 | 0 | 0 | 0 | 0 | 0 | 0 | 0 | 0 | 0 | | 0 | 0 | 0 | 0 |
| 17p13.3 (Dup) | 0 | 0 | 0 | 1 | 0 | 1 | 0 | 0 | 0 | 1 | 0 | 0 | 1 | 0 | 0 | 0 | 0 | 0 | 0 | 0 | 0 | 0 | 0 | 0 | 2 | 0 | | 0 | 0 | 0 | 1 |
| 17q11.2 (Del) | 0 | 0 | 0 | 0 | 0 | 0 | 0 | 0 | 0 | 0 | 0 | 0 | 0 | 0 | 0 | 0 | 0 | 0 | 0 | 0 | 0 | 1 | 0 | 0 | 0 | 0 | | 0 | 0 | 0 | 0 |
| 17q11.2 (Dup) | 0 | 0 | 0 | 0 | 0 | 0 | 0 | 0 | 0 | 0 | 0 | 0 | 0 | 0 | 0 | 0 | 0 | 0 | 0 | 0 | 0 | 0 | 0 | 0 | 0 | 0 | | 0 | 0 | 0 | 1 |
| 17q12 (Del) | 0 | 0 | 0 | 0 | 1 | 0 | 0 | 0 | 0 | 0 | 0 | 0 | 0 | 1 | 0 | 2 | 0 | 0 | 0 | 0 | 0 | 1 | 0 | 0 | 0 | 0 | | 0 | 0 | 0 | 0 |
| 17q12 (Dup) | 0 | 0 | 0 | 0 | 0 | 0 | 0 | 0 | 0 | 0 | 0 | 0 | 0 | 2 | 0 | 1 | 1 | 0 | 0 | 0 | 0 | 1 | 0 | 1 | 0 | 1 | | 0 | 0 | 0 | 0 |
| 17q21.31 (Del) | 0 | 0 | 0 | 0 | 0 | 0 | 0 | 0 | 0 | 0 | 0 | 0 | 0 | 0 | 0 | 0 | 0 | 0 | 0 | 0 | 0 | 1 | 0 | 0 | 0 | 0 | | 0 | 0 | 0 | 0 |
| 17q21.31 (Dup) | 0 | 0 | 0 | 0 | 1 | 0 | 0 | 0 | 1 | 0 | 0 | 0 | 0 | 0 | 0 | 0 | 0 | 0 | 0 | 0 | 0 | 0 | 0 | 0 | 0 | 0 | | 0 | 0 | 0 | 0 |
| 17q21.32 (Del) | 0 | 0 | 0 | 0 | 1 | 0 | 0 | 0 | 0 | 0 | 0 | 0 | 0 | 0 | 0 | 0 | 0 | 0 | 0 | 0 | 0 | 0 | 0 | 0 | 0 | 0 | | 0 | 0 | 0 | 0 |
| 17q24.2 (Dup) | 0 | 0 | 0 | 0 | 0 | 0 | 0 | 0 | 0 | 0 | 0 | 0 | 0 | 0 | 0 | 0 | 0 | 0 | 0 | 0 | 0 | 0 | 0 | 1 | 0 | 0 | | 0 | 0 | 0 | 0 |
| 17q25.3 (Del) | 0 | 0 | 0 | 0 | 2 | 0 | 0 | 0 | 0 | 0 | 0 | 0 | 0 | 0 | 0 | 0 | 0 | 0 | 0 | 0 | 0 | 0 | 0 | 1 | 0 | 0 | | 0 | 0 | 0 | 0 |
| 17q25.3 (Dup) | 0 | 0 | 0 | 0 | 0 | 2 | 0 | 0 | 0 | 0 | 0 | 0 | 0 | 0 | 0 | 0 | 0 | 0 | 0 | 0 | 0 | 0 | 0 | 2 | 0 | 0 | | 0 | 0 | 0 | 0 |
| 18p11.21 (Del) | 0 | 0 | 0 | 0 | 0 | 0 | 0 | 0 | 0 | 1 | 0 | 0 | 0 | 0 | 0 | 0 | 0 | 0 | 0 | 0 | 0 | 0 | 0 | 1 | 0 | 0 | | 0 | 0 | 0 | 0 |
| 18p11.22 (Dup) | 0 | 0 | 0 | 0 | 0 | 0 | 0 | 0 | 0 | 1 | 0 | 0 | 0 | 0 | 0 | 0 | 0 | 0 | 0 | 0 | 0 | 0 | 0 | 0 | 0 | 0 | | 0 | 0 | 0 | 0 |
| 18p11.23 (Dup) | 0 | 0 | 0 | 0 | 0 | 0 | 0 | 0 | 0 | 1 | 0 | 0 | 0 | 0 | 0 | 0 | 0 | 0 | 0 | 0 | 0 | 0 | 0 | 0 | 0 | 0 | | 0 | 0 | 0 | 0 |
| 18p11.31-p11.23 (Dup) | 0 | 0 | 0 | 0 | 0 | 1 | 0 | 0 | 0 | 0 | 0 | 0 | 0 | 0 | 0 | 0 | 0 | 0 | 0 | 0 | 0 | 0 | 0 | 0 | 0 | 0 | | 0 | 0 | 0 | 0 |
| 18p11.32 (Del) | 0 | 0 | 0 | 0 | 0 | 0 | 0 | 0 | 0 | 0 | 0 | 0 | 0 | 0 | 0 | 1 | 0 | 0 | 0 | 0 | 0 | 0 | 0 | 0 | 0 | 0 | | 0 | 0 | 0 | 0 |
| 18p11.32 (Dup) | 0 | 0 | 0 | 0 | 0 | 1 | 0 | 0 | 0 | 0 | 0 | 0 | 0 | 0 | 0 | 0 | 0 | 0 | 0 | 0 | 0 | 0 | 0 | 0 | 0 | 0 | | 0 | 0 | 0 | 0 |
| 18p11.32-p11.21 (Dup) | 0 | 0 | 0 | 0 | 0 | 0 | 0 | 0 | 0 | 0 | 0 | 0 | 0 | 0 | 0 | 0 | 0 | 0 | 0 | 0 | 0 | 0 | 0 | 0 | 0 | 1 | | 0 | 0 | 0 | 0 |
| 18p11.32-p11.23 (Del) | 0 | 0 | 0 | 0 | 0 | 0 | 0 | 0 | 0 | 0 | 0 | 0 | 0 | 0 | 0 | 0 | 0 | 0 | 0 | 0 | 0 | 0 | 0 | 0 | 0 | 2 | | 0 | 0 | 0 | 0 |
| 18q11.2 (Del) | 0 | 0 | 0 | 0 | 0 | 0 | 1 | 0 | 0 | 0 | 0 | 0 | 0 | 0 | 0 | 0 | 0 | 0 | 0 | 0 | 0 | 0 | 0 | 0 | 0 | 0 | | 0 | 0 | 0 | 0 |
| 18q12.2 (Del) | 0 | 0 | 0 | 0 | 0 | 0 | 0 | 0 | 0 | 0 | 0 | 0 | 0 | 0 | 0 | 0 | 0 | 0 | 0 | 0 | 0 | 0 | 0 | 2 | 0 | 0 | | 0 | 0 | 0 | 0 |
| 18q12.3 (Del) | 0 | 0 | 0 | 0 | 0 | 0 | 0 | 0 | 0 | 0 | 0 | 0 | 0 | 0 | 0 | 0 | 0 | 0 | 0 | 0 | 0 | 0 | 0 | 0 | 0 | 0 | | 8 | 0 | 0 | 0 |
| 18q12.3 (Dup) | 0 | 0 | 0 | 0 | 0 | 0 | 0 | 0 | 0 | 0 | 0 | 0 | 0 | 0 | 0 | 0 | 0 | 0 | 0 | 0 | 0 | 0 | 0 | 0 | 0 | 0 | | 8 | 0 | 0 | 0 |
| 18q21.31 (Dup) | 0 | 1 | 0 | 0 | 0 | 0 | 0 | 0 | 0 | 0 | 0 | 0 | 0 | 0 | 0 | 0 | 0 | 0 | 0 | 0 | 0 | 0 | 0 | 0 | 0 | 0 | | 0 | 0 | 0 | 0 |
| 18q22.2 (Del) | 0 | 0 | 0 | 1 | 0 | 0 | 0 | 0 | 0 | 0 | 0 | 0 | 0 | 0 | 0 | 0 | 0 | 0 | 0 | 0 | 0 | 0 | 0 | 0 | 0 | 0 | | 0 | 0 | 0 | 0 |
| 18q23 (Del) | 0 | 0 | 0 | 0 | 0 | 0 | 1 | 0 | 0 | 0 | 0 | 0 | 0 | 0 | 0 | 0 | 0 | 0 | 0 | 0 | 0 | 0 | 0 | 0 | 0 | 0 | | 0 | 0 | 0 | 0 |
| 18q23 (Dup) | 0 | 0 | 0 | 0 | 0 | 0 | 0 | 0 | 2 | 0 | 0 | 0 | 0 | 0 | 0 | 0 | 0 | 0 | 0 | 0 | 0 | 0 | 0 | 0 | 0 | 0 | | 0 | 0 | 0 | 0 |
| 18q23.2 (Del) | 0 | 0 | 1 | 0 | 0 | 0 | 0 | 0 | 0 | 0 | 0 | 0 | 0 | 0 | 0 | 0 | 0 | 0 | 0 | 0 | 0 | 0 | 0 | 0 | 0 | 0 | | 0 | 0 | 0 | 0 |
| 19p12 (Dup) | 0 | 0 | 0 | 0 | 0 | 0 | 1 | 0 | 0 | 0 | 0 | 0 | 0 | 0 | 0 | 0 | 0 | 0 | 0 | 0 | 0 | 0 | 0 | 0 | 0 | 0 | | 0 | 0 | 0 | 0 |
| 19p13.11 (Dup) | 0 | 0 | 0 | 0 | 0 | 0 | 0 | 0 | 0 | 0 | 0 | 0 | 0 | 0 | 0 | 0 | 0 | 0 | 0 | 0 | 0 | 0 | 0 | 0 | 2 | 0 | | 0 | 0 | 0 | 0 |
| 19p13.2 (Dup) | 0 | 0 | 0 | 0 | 0 | 0 | 0 | 0 | 1 | 0 | 0 | 0 | 0 | 0 | 0 | 1 | 0 | 0 | 0 | 0 | 0 | 0 | 0 | 0 | 0 | 0 | | 0 | 0 | 0 | 0 |
| 19p13.3 (Del) | 0 | 0 | 0 | 0 | 0 | 0 | 0 | 0 | 0 | 0 | 0 | 0 | 0 | 0 | 0 | 0 | 0 | 0 | 0 | 0 | 0 | 0 | 0 | 1 | 0 | 0 | | 0 | 0 | 0 | 0 |
| 19q13.32 (Del) | 0 | 0 | 0 | 0 | 0 | 0 | 0 | 0 | 0 | 0 | 0 | 0 | 0 | 0 | 0 | 0 | 0 | 0 | 0 | 0 | 0 | 0 | 0 | 1 | 0 | 0 | | 0 | 0 | 0 | 0 |
| 19q13.32 (Dup) | 0 | 0 | 0 | 0 | 0 | 0 | 0 | 0 | 0 | 0 | 0 | 0 | 0 | 0 | 0 | 0 | 0 | 0 | 0 | 0 | 0 | 0 | 0 | 1 | 3 | 0 | | 0 | 0 | 0 | 0 |
| 19q13.41 (Del) | 0 | 0 | 0 | 0 | 1 | 0 | 0 | 0 | 0 | 0 | 0 | 0 | 0 | 0 | 0 | 0 | 0 | 0 | 0 | 0 | 0 | 0 | 0 | 0 | 0 | 0 | | 0 | 0 | 0 | 0 |
| 19q13.42 (Dup) | 0 | 0 | 0 | 0 | 0 | 0 | 0 | 0 | 0 | 0 | 0 | 0 | 0 | 0 | 0 | 0 | 0 | 0 | 0 | 0 | 0 | 0 | 0 | 1 | 0 | 0 | | 0 | 0 | 0 | 0 |
| 19q13.42-q13.43 (Dup) | 0 | 0 | 0 | 0 | 0 | 1 | 0 | 0 | 0 | 0 | 0 | 0 | 0 | 0 | 0 | 0 | 0 | 0 | 0 | 0 | 0 | 0 | 0 | 0 | 0 | 0 | | 0 | 0 | 0 | 0 |
| 20p12.1 (Del) | 0 | 0 | 0 | 0 | 0 | 0 | 0 | 0 | 1 | 0 | 0 | 1 | 0 | 0 | 0 | 0 | 0 | 0 | 0 | 0 | 0 | 0 | 0 | 7 | 0 | 0 | | 0 | 0 | 0 | 0 |
| 20p12.3 (Del) | 0 | 0 | 0 | 0 | 0 | 0 | 0 | 0 | 0 | 0 | 0 | 0 | 0 | 0 | 0 | 0 | 0 | 0 | 0 | 0 | 1 | 0 | 0 | 1 | 0 | 0 | | 0 | 0 | 0 | 0 |
| 20p13 (Del) | 0 | 0 | 0 | 0 | 0 | 0 | 0 | 0 | 0 | 0 | 0 | 0 | 0 | 0 | 0 | 0 | 0 | 0 | 0 | 0 | 0 | 0 | 0 | 0 | 0 | 0 | | 0 | 2 | 0 | 0 |
| 20p13.1 (Del) | 0 | 0 | 1 | 0 | 0 | 0 | 0 | 0 | 0 | 0 | 0 | 0 | 0 | 0 | 0 | 0 | 0 | 0 | 0 | 0 | 0 | 0 | 0 | 0 | 0 | 0 | | 0 | 0 | 0 | 0 |
| 20p13.3 (Dup) | 0 | 0 | 0 | 0 | 0 | 0 | 0 | 0 | 0 | 1 | 0 | 0 | 0 | 0 | 0 | 0 | 0 | 0 | 0 | 0 | 0 | 0 | 0 | 0 | 0 | 0 | | 0 | 0 | 0 | 0 |
| 20p13-p12.3 (Dup) | 0 | 0 | 0 | 0 | 0 | 0 | 0 | 0 | 0 | 0 | 0 | 0 | 0 | 0 | 0 | 0 | 0 | 0 | 0 | 0 | 0 | 0 | 0 | 0 | 0 | 0 | | 0 | 1 | 0 | 0 |
| 20q11.22 (Dup) | 0 | 0 | 0 | 0 | 0 | 0 | 0 | 0 | 0 | 0 | 0 | 0 | 0 | 0 | 0 | 0 | 0 | 0 | 0 | 8 | 0 | 0 | 0 | 0 | 0 | 0 | | 0 | 0 | 0 | 0 |
| 20q13.31-q13.33 (Dup) | 0 | 0 | 0 | 0 | 0 | 0 | 0 | 0 | 0 | 0 | 0 | 0 | 0 | 0 | 1 | 0 | 0 | 0 | 0 | 0 | 0 | 0 | 0 | 0 | 0 | 0 | | 0 | 0 | 0 | 0 |
| 20q13.32-q13.33 (Dup) | 0 | 0 | 0 | 0 | 0 | 1 | 0 | 0 | 0 | 0 | 0 | 0 | 0 | 0 | 0 | 0 | 0 | 0 | 0 | 0 | 0 | 0 | 0 | 0 | 0 | 0 | | 0 | 0 | 0 | 0 |
| 21q11.2-q22.3 (Dup) | 0 | 0 | 1 | 0 | 0 | 0 | 0 | 0 | 0 | 0 | 0 | 0 | 0 | 0 | 0 | 0 | 0 | 0 | 0 | 0 | 0 | 0 | 0 | 0 | 0 | 0 | | 0 | 0 | 0 | 0 |
| 21q21.1 (Dup) | 0 | 0 | 0 | 0 | 0 | 0 | 0 | 0 | 0 | 0 | 0 | 0 | 0 | 0 | 0 | 0 | 0 | 0 | 0 | 0 | 0 | 0 | 0 | 1 | 0 | 0 | | 0 | 0 | 0 | 0 |
| 21q21.3-q21.11 (Del) | 0 | 0 | 0 | 0 | 0 | 0 | 1 | 0 | 0 | 0 | 0 | 0 | 0 | 0 | 0 | 0 | 0 | 0 | 0 | 0 | 0 | 0 | 0 | 0 | 0 | 0 | | 0 | 0 | 0 | 0 |
| 21q22.2-q22.3 (Del) | 0 | 0 | 0 | 0 | 0 | 0 | 0 | 0 | 0 | 0 | 0 | 0 | 0 | 0 | 0 | 1 | 0 | 0 | 0 | 0 | 0 | 0 | 0 | 0 | 0 | 0 | | 0 | 0 | 0 | 0 |
| 21q22.3 (Del) | 0 | 0 | 0 | 0 | 0 | 0 | 0 | 0 | 1 | 0 | 0 | 0 | 0 | 0 | 0 | 0 | 0 | 0 | 0 | 0 | 0 | 0 | 0 | 0 | 0 | 0 | | 0 | 0 | 0 | 0 |
| 21q22.3 (Dup) | 0 | 0 | 0 | 0 | 0 | 0 | 0 | 0 | 0 | 0 | 0 | 0 | 0 | 0 | 0 | 1 | 0 | 0 | 0 | 0 | 2 | 0 | 0 | 0 | 0 | 0 | | 0 | 0 | 0 | 0 |
| 22q11.1 (Del) | 0 | 0 | 0 | 0 | 2 | 0 | 0 | 0 | 0 | 0 | 0 | 0 | 0 | 0 | 0 | 0 | 0 | 0 | 0 | 0 | 0 | 0 | 0 | 1 | 0 | 0 | | 0 | 0 | 0 | 0 |
| 22q11.1 (Dup) | 0 | 0 | 0 | 0 | 0 | 1 | 0 | 0 | 0 | 0 | 0 | 0 | 0 | 0 | 0 | 0 | 0 | 0 | 0 | 0 | 0 | 0 | 0 | 0 | 0 | 0 | | 0 | 0 | 0 | 0 |
| 22q11.2 (Del) | 0 | 0 | 0 | 0 | 0 | 0 | 0 | 0 | 0 | 1 | 3 | 0 | 0 | 0 | 0 | 0 | 0 | 0 | 1 | 0 | 0 | 2 | 0 | 0 | 0 | 0 | | 0 | 0 | 0 | 1 |
| 22q11.2 (Dup) | 0 | 0 | 0 | 0 | 0 | 0 | 0 | 0 | 0 | 0 | 0 | 0 | 0 | 0 | 0 | 0 | 0 | 0 | 3 | 0 | 0 | 2 | 0 | 0 | 0 | 0 | | 0 | 0 | 0 | 0 |
| 22q11.21 (Del) | 0 | 0 | 0 | 0 | 0 | 1 | 0 | 0 | 1 | 0 | 0 | 0 | 0 | 3 | 0 | 5 | 0 | 0 | 0 | 0 | 0 | 0 | 0 | 2 | 0 | 0 | | 0 | 0 | 0 | 0 |
| 22q11.21 (Dup) | 0 | 0 | 0 | 1 | 0 | 2 | 1 | 0 | 0 | 0 | 0 | 0 | 0 | 0 | 0 | 0 | 0 | 0 | 0 | 0 | 1 | 0 | 0 | 6 | 0 | 0 | | 0 | 0 | 0 | 0 |
| 22q11.21-q11.22 (Del) | 0 | 0 | 0 | 1 | 0 | 1 | 0 | 0 | 0 | 0 | 0 | 0 | 0 | 0 | 0 | 0 | 0 | 0 | 0 | 0 | 0 | 0 | 0 | 0 | 0 | 0 | | 0 | 0 | 0 | 0 |
| 22q12.1 (Dup) | 0 | 0 | 0 | 0 | 0 | 0 | 0 | 0 | 0 | 0 | 0 | 0 | 0 | 0 | 0 | 0 | 0 | 0 | 0 | 0 | 0 | 0 | 0 | 1 | 0 | 0 | | 0 | 0 | 0 | 0 |
| 22q13 (Del) | 0 | 0 | 0 | 1 | 0 | 0 | 0 | 0 | 0 | 0 | 0 | 0 | 0 | 0 | 0 | 0 | 0 | 0 | 0 | 0 | 0 | 0 | 0 | 0 | 0 | 0 | | 0 | 0 | 0 | 0 |
| 22q13.1 (Del) | 0 | 0 | 0 | 0 | 1 | 0 | 0 | 0 | 0 | 0 | 0 | 0 | 0 | 0 | 0 | 0 | 0 | 0 | 0 | 0 | 0 | 0 | 0 | 0 | 0 | 0 | | 0 | 0 | 1 | 0 |
| 22q13.1-q13.33 (Dup) | 0 | 0 | 1 | 0 | 0 | 0 | 0 | 0 | 0 | 0 | 0 | 0 | 0 | 0 | 0 | 0 | 0 | 0 | 0 | 0 | 0 | 0 | 0 | 0 | 0 | 0 | | 0 | 0 | 0 | 0 |
| 22q13.31 (Del) | 0 | 0 | 0 | 0 | 0 | 0 | 0 | 0 | 0 | 0 | 0 | 0 | 0 | 0 | 0 | 0 | 0 | 0 | 1 | 0 | 0 | 0 | 0 | 0 | 0 | 0 | | 0 | 0 | 0 | 0 |
| 22q13.31 (Dup) | 0 | 0 | 0 | 0 | 0 | 0 | 0 | 0 | 0 | 0 | 0 | 0 | 0 | 1 | 0 | 0 | 0 | 0 | 1 | 0 | 0 | 0 | 0 | 0 | 0 | 0 | | 0 | 0 | 0 | 0 |
| 22q13.31-q13.33 (Del) | 0 | 0 | 0 | 0 | 0 | 0 | 0 | 0 | 0 | 1 | 0 | 0 | 0 | 0 | 1 | 0 | 0 | 0 | 0 | 0 | 0 | 0 | 0 | 0 | 0 | 0 | | 0 | 1 | 0 | 0 |
| 22q13.31-q13.33 (Dup) | 6 | 0 | 0 | 0 | 0 | 0 | 0 | 0 | 0 | 0 | 0 | 0 | 0 | 0 | 0 | 0 | 0 | 0 | 0 | 0 | 0 | 0 | 0 | 0 | 0 | 0 | | 0 | 0 | 0 | 0 |
| 22q13.32 (Del) | 0 | 0 | 0 | 0 | 0 | 0 | 0 | 0 | 0 | 0 | 0 | 0 | 0 | 1 | 0 | 0 | 0 | 0 | 0 | 0 | 0 | 0 | 0 | 0 | 0 | 0 | | 0 | 0 | 0 | 0 |
| 22q13.32 (Dup) | 0 | 0 | 0 | 0 | 0 | 0 | 0 | 0 | 0 | 0 | 0 | 0 | 0 | 1 | 0 | 0 | 0 | 0 | 0 | 0 | 0 | 0 | 0 | 0 | 0 | 0 | | 0 | 0 | 0 | 0 |
| 22q13.32-q13.33 (Dup) | 0 | 0 | 0 | 0 | 0 | 1 | 0 | 0 | 0 | 0 | 0 | 0 | 0 | 0 | 0 | 0 | 0 | 0 | 0 | 0 | 0 | 0 | 0 | 0 | 0 | 0 | | 0 | 0 | 0 | 0 |
| 22q13.33 (Del) | 0 | 0 | 1 | 0 | 0 | 2 | 0 | 0 | 0 | 1 | 0 | 0 | 0 | 0 | 0 | 0 | 0 | 0 | 0 | 0 | 0 | 0 | 0 | 0 | 0 | 4 | | 0 | 0 | 0 | 0 |
| 22q13.33 (Dup) | 0 | 0 | 0 | 0 | 0 | 0 | 0 | 0 | 0 | 0 | 0 | 0 | 0 | 0 | 0 | 0 | 0 | 0 | 0 | 0 | 0 | 0 | 0 | 1 | 0 | 0 | | 0 | 0 | 0 | 0 |
| Xp11.21-11.1 (Dup) | 0 | 0 | 0 | 0 | 0 | 1 | 0 | 0 | 0 | 0 | 0 | 0 | 0 | 0 | 0 | 0 | 0 | 0 | 0 | 0 | 0 | 0 | 0 | 0 | 0 | 0 | | 0 | 0 | 0 | 0 |
| Xp11.22 (Dup) | 0 | 0 | 0 | 0 | 0 | 0 | 0 | 0 | 0 | 0 | 0 | 0 | 0 | 0 | 0 | 0 | 0 | 0 | 0 | 0 | 1 | 0 | 0 | 0 | 0 | 0 | | 0 | 0 | 0 | 0 |
| Xp11.3 (Dup) | 0 | 0 | 0 | 0 | 0 | 0 | 0 | 0 | 0 | 0 | 0 | 0 | 0 | 0 | 0 | 0 | 0 | 0 | 0 | 0 | 0 | 0 | 0 | 1 | 0 | 0 | | 0 | 0 | 0 | 0 |
| Xp11.4 (Dup) | 0 | 0 | 0 | 0 | 0 | 0 | 0 | 0 | 1 | 1 | 0 | 0 | 0 | 0 | 0 | 0 | 0 | 0 | 0 | 0 | 0 | 0 | 0 | 1 | 0 | 0 | | 0 | 0 | 0 | 0 |
| Xp21.1 (Del) | 0 | 0 | 0 | 0 | 0 | 0 | 0 | 0 | 0 | 0 | 0 | 0 | 2 | 0 | 0 | 0 | 0 | 0 | 0 | 0 | 0 | 0 | 0 | 1 | 2 | 0 | | 0 | 0 | 0 | 0 |
| Xp21.1 (Dup) | 0 | 0 | 0 | 0 | 0 | 0 | 0 | 0 | 0 | 0 | 0 | 0 | 0 | 0 | 0 | 0 | 0 | 0 | 0 | 0 | 0 | 0 | 0 | 2 | 0 | 0 | | 0 | 0 | 0 | 0 |
| Xp21.2 (Del) | 0 | 0 | 0 | 0 | 0 | 0 | 0 | 0 | 0 | 0 | 0 | 0 | 0 | 0 | 0 | 0 | 0 | 0 | 0 | 0 | 0 | 0 | 0 | 1 | 0 | 0 | | 0 | 0 | 0 | 0 |
| Xp21.3 (Del) | 0 | 0 | 0 | 0 | 0 | 0 | 0 | 0 | 0 | 0 | 0 | 0 | 0 | 0 | 0 | 0 | 0 | 0 | 0 | 0 | 0 | 0 | 0 | 1 | 0 | 0 | | 0 | 0 | 0 | 0 |
| Xp21.3-p21.2 (Dup) | 0 | 0 | 0 | 0 | 0 | 0 | 0 | 0 | 0 | 0 | 0 | 0 | 0 | 0 | 0 | 0 | 0 | 0 | 0 | 0 | 0 | 0 | 0 | 1 | 0 | 0 | | 0 | 0 | 0 | 0 |
| Xp22.11 (Del) | 0 | 0 | 0 | 0 | 0 | 0 | 0 | 0 | 0 | 0 | 0 | 0 | 0 | 0 | 0 | 0 | 0 | 0 | 0 | 0 | 0 | 0 | 0 | 6 | 0 | 0 | | 0 | 0 | 0 | 0 |
| Xp22.11-p21.3 (Dup) | 0 | 0 | 0 | 0 | 0 | 0 | 0 | 0 | 1 | 0 | 0 | 0 | 0 | 0 | 0 | 0 | 0 | 0 | 0 | 0 | 0 | 0 | 0 | 0 | 0 | 0 | | 0 | 0 | 0 | 0 |
| Xp22.12 (Dup) | 0 | 0 | 0 | 0 | 0 | 0 | 0 | 0 | 0 | 0 | 0 | 0 | 0 | 0 | 0 | 0 | 0 | 0 | 0 | 0 | 0 | 0 | 0 | 1 | 0 | 0 | | 0 | 0 | 0 | 0 |
| Xp22.2 (Del) | 0 | 0 | 0 | 0 | 0 | 0 | 0 | 0 | 0 | 0 | 0 | 0 | 0 | 0 | 0 | 0 | 0 | 0 | 0 | 0 | 0 | 0 | 0 | 1 | 0 | 0 | | 0 | 0 | 1 | 0 |
| Xp22.2 (Dup) | 0 | 0 | 0 | 0 | 0 | 0 | 1 | 0 | 0 | 0 | 0 | 0 | 0 | 0 | 0 | 0 | 0 | 0 | 0 | 0 | 0 | 0 | 0 | 1 | 2 | 0 | | 0 | 0 | 0 | 0 |
| Xp22.31 (Del) | 0 | 0 | 0 | 0 | 0 | 2 | 0 | 0 | 0 | 0 | 0 | 0 | 0 | 0 | 0 | 0 | 0 | 0 | 0 | 0 | 1 | 0 | 0 | 0 | 0 | 0 | | 0 | 0 | 0 | 1 |
| Xp22.31 (Dup) | 0 | 0 | 0 | 0 | 0 | 2 | 0 | 0 | 0 | 0 | 0 | 0 | 0 | 0 | 0 | 0 | 0 | 0 | 0 | 0 | 0 | 0 | 0 | 0 | 0 | 0 | | 0 | 0 | 0 | 0 |
| Xp22.32-22.31 (Del) | 0 | 0 | 0 | 0 | 0 | 0 | 0 | 0 | 0 | 0 | 0 | 0 | 0 | 0 | 1 | 0 | 0 | 0 | 0 | 0 | 0 | 0 | 0 | 0 | 0 | 0 | | 0 | 0 | 0 | 0 |
| Xp22.33 (Del) | 0 | 0 | 0 | 0 | 0 | 0 | 0 | 0 | 0 | 0 | 0 | 0 | 0 | 0 | 0 | 0 | 0 | 1 | 0 | 0 | 0 | 0 | 0 | 0 | 0 | 0 | | 0 | 0 | 0 | 0 |
| Xp22.33 (Dup) | 0 | 2 | 0 | 0 | 0 | 0 | 0 | 0 | 0 | 0 | 0 | 0 | 0 | 0 | 0 | 0 | 0 | 0 | 0 | 0 | 1 | 0 | 0 | 0 | 0 | 0 | | 0 | 0 | 0 | 0 |
| Xp22.33-22.31 (Del) | 0 | 0 | 0 | 0 | 0 | 0 | 0 | 0 | 0 | 0 | 0 | 0 | 0 | 0 | 1 | 0 | 0 | 0 | 0 | 0 | 0 | 0 | 0 | 0 | 0 | 0 | | 0 | 0 | 0 | 0 |
| Xp22.33-p22.11 (Dup) | 0 | 0 | 0 | 0 | 0 | 0 | 0 | 0 | 0 | 0 | 0 | 0 | 0 | 0 | 0 | 1 | 0 | 0 | 0 | 0 | 0 | 0 | 0 | 0 | 0 | 0 | | 0 | 0 | 0 | 0 |
| Xq12 (Del) | 0 | 0 | 0 | 0 | 1 | 0 | 0 | 0 | 0 | 0 | 0 | 0 | 0 | 0 | 0 | 0 | 0 | 0 | 0 | 0 | 0 | 0 | 0 | 0 | 0 | 0 | | 0 | 0 | 0 | 0 |
| Xq12-q13.1 (Dup) | 1 | 0 | 0 | 0 | 0 | 1 | 0 | 0 | 0 | 0 | 0 | 0 | 0 | 0 | 0 | 0 | 0 | 0 | 0 | 0 | 0 | 0 | 0 | 0 | 0 | 0 | | 0 | 0 | 0 | 0 |
| Xq13.2 (Del) | 0 | 0 | 0 | 0 | 0 | 0 | 0 | 0 | 0 | 0 | 0 | 0 | 1 | 0 | 0 | 0 | 0 | 0 | 0 | 0 | 0 | 0 | 0 | 0 | 0 | 0 | | 0 | 0 | 0 | 0 |
| Xq21.1 (Dup) | 0 | 0 | 0 | 0 | 0 | 0 | 0 | 0 | 0 | 0 | 0 | 0 | 0 | 0 | 0 | 0 | 0 | 0 | 0 | 0 | 0 | 0 | 0 | 1 | 0 | 0 | | 0 | 0 | 0 | 0 |
| Xq21.33 (Dup) | 0 | 0 | 0 | 0 | 0 | 1 | 0 | 0 | 0 | 0 | 0 | 0 | 0 | 0 | 0 | 0 | 0 | 0 | 0 | 0 | 0 | 0 | 0 | 1 | 0 | 0 | | 0 | 0 | 0 | 0 |
| Xq22.3 (Dup) | 0 | 0 | 0 | 0 | 0 | 0 | 0 | 0 | 0 | 0 | 0 | 0 | 0 | 0 | 0 | 0 | 0 | 0 | 0 | 0 | 0 | 0 | 0 | 1 | 0 | 0 | | 0 | 0 | 0 | 0 |
| Xq26.2 (Dup) | 0 | 0 | 0 | 1 | 0 | 0 | 0 | 0 | 0 | 0 | 0 | 0 | 0 | 0 | 0 | 0 | 0 | 0 | 0 | 0 | 0 | 0 | 0 | 0 | 0 | 0 | | 0 | 0 | 0 | 0 |
| Xq27.1 (Dup) | 0 | 0 | 0 | 0 | 0 | 0 | 0 | 0 | 0 | 0 | 0 | 0 | 0 | 0 | 0 | 0 | 0 | 0 | 0 | 0 | 0 | 0 | 0 | 1 | 0 | 0 | | 0 | 0 | 0 | 0 |
| Xq28 (Del) | 0 | 0 | 0 | 0 | 2 | 0 | 0 | 0 | 0 | 0 | 0 | 0 | 0 | 0 | 0 | 0 | 0 | 0 | 0 | 0 | 0 | 0 | 0 | 4 | 0 | 0 | | 0 | 0 | 0 | 0 |
| Xq28 (Dup) | 0 | 0 | 1 | 1 | 0 | 0 | 1 | 0 | 0 | 0 | 0 | 0 | 0 | 0 | 0 | 0 | 0 | 0 | 0 | 0 | 0 | 0 | 0 | 1 | 0 | 0 | | 0 | 0 | 0 | 0 |
| Yp11.32 (Dup) | 0 | 0 | 0 | 0 | 0 | 0 | 0 | 0 | 0 | 0 | 0 | 0 | 0 | 0 | 0 | 0 | 0 | 0 | 0 | 0 | 1 | 0 | 0 | 0 | 0 | 0 | | 0 | 0 | 0 | 0 |

We present first author and country of sample/nation of each study. Qiu (China) as highlighted, represents our study. The others are previous sudies. The number is ASD patients with corresponding CNV.

AlAyadhi (Saudi Arabia): High-resolution SNP genotyping platform identified recurrent and novel CNVs in autism multiplex families. Neuroscience. 2016 Dec 17;339:561-570.

Bitar  (Lebanon): Identification of rare copy number variations reveals PJA2, APCS, SYNPO, and TAC1 as novel candidate genes in Autism Spectrum Disorders. Mol Genet Genomic Med. 2019 Aug;7(8):e786.

Bremer  (Sweden): Copy number variation characteristics in subpopulations of patients with autism spectrum disorders. Am J Med Genet B Neuropsychiatr Genet. 2011 Mar;156(2):115-24.

Celestino-Soper (USA): Use of array CGH to detect exonic copy number variants throughout the genome in autism families detects a novel deletion in TMLHE. Hum Mol Genet. 2011 Nov 15;20(22):4360-70.

Chen (China): High resolution analysis of rare copy number variants in patients with autism spectrum disorder from Taiwan. Sci Rep. 2017 Sep 20;7(1):11919.

Costa (Canada): Copy number variations in a Brazilian cohort with autism spectrum disorders highlight the contribution of cell adhesion genes. Clin Genet. 2021 Oct 19.

Davis (USA): Novel copy number variants in children with autism and additional developmental anomalies. J Neurodev Disord. 2009 Dec;1(4):292-301.

Egger  (Austria): Identification of risk genes for autism spectrum disorder through copy number variation analysis in Austrian families. Neurogenetics. 2014 May;15(2):117-27.

Eriksson (Sweden): Rare copy number variants are common in young children with autism spectrum disorder. Acta Paediatr. 2015 Jun;104(6):610-8.

Fan (China): Rare Copy Number Variations in a Chinese Cohort of Autism Spectrum Disorder. Front Genet. 2018 Dec 18;9:665.

Görker (Turkey): Investigation of Copy Number Variation by arrayCGH in Turkish Children and Adolescents Diagnosed with Autism Spectrum Disorders. Noro Psikiyatr Ars. 2018 Apr 26;55(3):215-219.

Gazzellone (China): Copy number variation in Han Chinese individuals with autism spectrum disorder. J Neurodev Disord. 2014;6(1):34.

Girirajan (USA): Refinement and discovery of new hotspots of copy-number variation associated with autism spectrum disorder. Am J Hum Genet. 2013 Feb 7;92(2):221-37.

Guo (China): Genome-wide copy number variation analysis in a Chinese autism spectrum disorder cohort. Sci Rep. 2017 Mar 10;7:44155.

Kushima (Japan): Comparative Analyses of Copy-Number Variation in Autism Spectrum Disorder and Schizophrenia Reveal Etiological Overlap and Biological Insights. Cell Rep. 2018 Sep 11;24(11):2838-2856.

Leppa (USA): Rare Inherited and De Novo CNVs Reveal Complex Contributions to ASD Risk in Multiplex Families. Am J Hum Genet. 2016 Sep 1;99(3):540-554.

Mak (China): Use of clinical chromosomal microarray in Chinese patients with autism spectrum disorder-implications of a copy number variation involving DPP10. Mol Autism. 2017 Jun 26;8:31.

Marshall (Canada): Structural variation of chromosomes in autism spectrum disorder. Am J Hum Genet. 2008 Feb;82(2):477-88.

Matsunami (USA): Identification of rare recurrent copy number variants in high-risk autism families and their prevalence in a large ASD population. PLoS One. 2013;8(1):e52239.

Monteiro (Portugal): Identification of Copy Number Variation by Array-CGH in Portuguese Children and Adolescents Diagnosed with Autism Spectrum Disorders. Neuropediatrics. 2019 Dec;50(6):367-377.

Moreno-De-Luca (USA): Using large clinical data sets to infer pathogenicity for rare copy number variants in autism cohorts. Mol Psychiatry. 2013 Oct;18(10):1090-5.

Nava (Asia/Europe/South America/Sub-Saharan Africa/Comoros): Prospective diagnostic analysis of copy number variants using SNP microarrays in individuals with autism spectrum disorders. Eur J Hum Genet. 2014 Jan;22(1):71-8.

Pinto  (European ancestry): Functional impact of global rare copy number variation in autism spectrum disorders. Nature. 2010 Jul 15;466(7304):368-72.

Prasad  (Canada): A discovery resource of rare copy number variations in individuals with autism spectrum disorder. G3 (Bethesda). 2012 Dec;2(12):1665-85.

Rosenfeld (USA): Copy number variations associated with autism spectrum disorders contribute to a spectrum of neurodevelopmental disorders. Genet Med. 2010 Nov;12(11):694-702.

Sakamoto (Japan): Copy number variations in Japanese children with autism spectrum disorder. Psychiatr Genet. 2021 Jun 1;31(3):79-87.

Sebat (USA): Strong association of de novo copy number mutations with autism. Science. 2007 Apr 20;316(5823):445-9.

Sorte  (Norway): Copy number variation findings among 50 children and adolescents with autism spectrum disorder. Psychiatr Genet. 2013 Apr;23(2):61-9.

Yep (Australia): Analysis of common genetic variation and rare CNVs in the Australian Autism Biobank. Mol Autism. 2021 Feb 10;12(1):12.


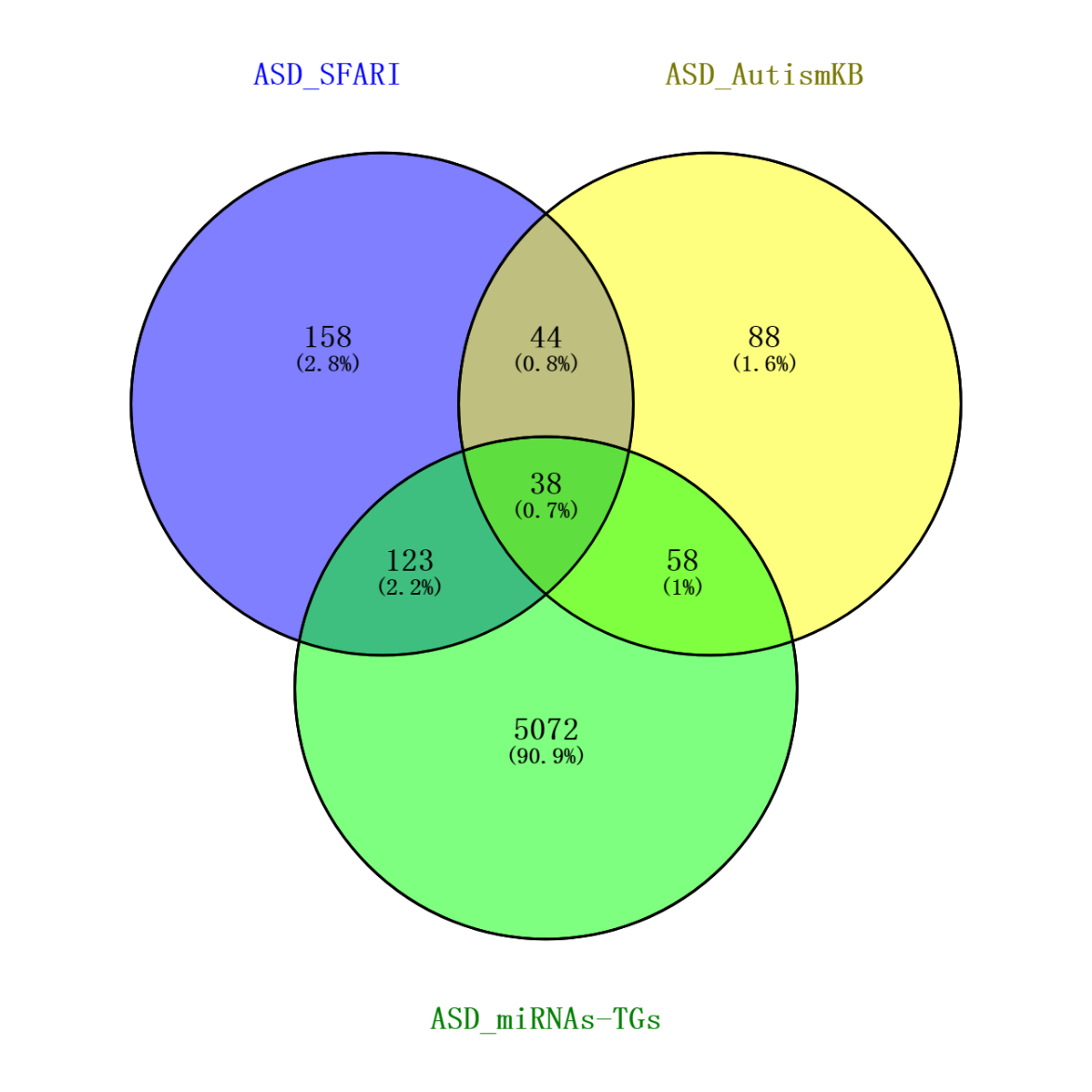


Supplementary Fig. 1. Venn diagram based on ASD_SFARI, ASD_AutismKB, and CNVs-encoded-miRNAs-targeted genes

Note: We denote CNVs-encoded-miRNAs-targeted genes for ASD as “ASD_ miRNAs-TGs”, the 363 high confidence and strong candidate autism risk genes in SFARI as “ASD_SFARI”, and the 228 high confidence autism related genes in AutismKB as “ASD_AutismKB”.
